# Supplementary material for: Learning to detect AI texts and learning the limits
Source: PLoS One. 2025 Oct 15;20(10):e0333007. doi: 10.1371/journal.pone.0333007 (PMC12527182; doi:10.1371/journal.pone.0333007)
Supplement: S1 File — Detailed description of the experiment and all methods and techniques to obtain and analyze the data, descriptive statistics of the data. (PDF) [file pone.0333007.s001.pdf]

Protocol:
Humans can learn to detect AI-generated texts, or at least learn when they can't

Jiří Milička

1 Introduction

This document describes the data collection process for the article *Humans can learn to detect AI-generated texts, or at least learn when they can't.* Our goal is to ensure transparency and reproducibility of our research methods, allowing for validation and replication of our findings.

For inquiries about the study, please contact the appropriate authors: Jiří Milička for technical and theoretical questions; Anna Marklová, Eva Pospíšilová, and Ondřej Drobil for experiment design matters.

All data, scripts, programs, and datasets from this study are available as Supplementary Material and released under the Creative Commons Attribution-ShareAlike 4.0 International License (CC BY-SA 4.0): https://creativecommons.org/licenses/by-sa/4.0/.

1.1 Ethics Committee Approval

This study was approved by the Ethics Committee of the Faculty of Arts at Charles University, Prague. Details are available in Appendix A and Appendix B.The Informed Consent document that participants agreed to is included in Appendix C.

As of the time of writing, no ethics committee approval is required for experimenting on Large Language Models (LLMs). However, we have taken great care to treat the LLMs with the same level of respect and consideration as we would humans.

2 Hypotheses

Our original hypotheses are as follows:

1. Czech speakers, when presented with two texts, one written by a human and one AI generated, will be able to guess which is which with higher than chance accuracy.

2. Individuals who receive immediate feedback after each trial in the aforementioned task will demonstrate higher accuracy than those who do not receive feedback.

3. Individuals who receive immediate feedback after each trial in the aforementioned task will demonstrate stronger positive relationship between confidence level and accuracy than those who do not receive feedback.

4. The accuracy of guessing whether a text was written by a human or AI will be influenced by the stylometric qualities of the texts. The question of which qualities influence the accuracy is exploratory.

5. Human-written and AI-generated texts will differ in percieved readability measured during the experiment. The question of how percieved readability influence the authorship assessment correctness is exploratory.

6. Individuals with a more positive attitude toward AI will be more successful in determining authorship.

7. Individuals who interact with AI more frequently will be more successful in determining authorship.

3 Experiment Design

The study was conducted online through a dedicated application, allowing participants to complete it in their own environment. After consenting to the experiment and acknowledging the Informed Consent document (see Appendix C), participants were randomly assigned to one of two groups. The first group received feedback after each trial, enabling them to learn from their mistakes, while the second group received feedback only at the end.

Participants provided basic demographic information (age, gender, education), reported any reading difficulties, their frequency of AI interaction, and completed a battery of questions assessing their attitudes toward AI.

Following a brief instruction on how to respond to questions, participants completed 20 trials. Of these, 17 were texts randomly selected from the corpus, and 3 were attention-check texts (positioned at trials 3, 13, and 18) designed to verify participants' attentiveness. These attention checks were excluded from analysis and used only to filter out inattentive participants from the dataset.

3.1 Experimental Application

The application frontend was developed using HTML, JavaScript, and CSS. Interested parties can view it, including its source code, at `https://hypertext.cz/experimentcz`. We make this application available under the Creative Commons Attribution-ShareAlike 4.0 International License (CC BY-SA 4.0): https://creativecommons.org/licenses/by-sa/4.0/.

The backend cannot be made available as it is part of a larger system. However, given the simple architecture, recreating it should not be difficult. The application first loads a file containing all possible questions from the server, after which all operations, including randomization and administration of individual trials, occur client-side. The only other server interaction happens at the end, when participants confirm that their experimental data may be sent to the server. This design allows participants to terminate the experiment at any time without any data being stored. The only two server interactions are the initial request for the complete dataset and the final request to save the resulting dataset, both conducted through standard HTTP requests.

4 Language Selection

We chose not to conduct the experiment in English as it is unrepresentative of the majority of the world's population. LLMs are predominantly anglophone-centric, with English serving as the primary dataset for both initial training and subsequent instruction tuning or RLHF (Reinforcement Learning from Human Feedback). We selected Czech as our experimental language to examine how people handle texts generated in a medium-sized language. Additionally, Czech is the native language of all team members, allowing us to operate within the Czech linguistic environment and obtain valuable feedback.

5 Corpus Selection and Preparation

The *Koditex* corpus proved ideal as a source of human-produced texts, as it was designed as a multi-genre collection with numerous samples from various text types. Corpus details are available at `koditex`.

Koditex served as the source of prompts for our generated corpus. Each text in Koditex was divided into two parts, with the first 500 words paired with the system prompt: "Please continue the Czech text in the same language, manner and style, ensuring it contains at least five thousand words. The text does not need to be factually correct, but please make sure it fits stylistically."

Texts were generated using the `gpt-4o` model with temperature = 0 (namely GPT-4o-2024-05-13).

This newly generated corpus is available separately as a subcorpus of *Pseudokoditex* at `https://wiki.korpus.cz/doku.php/en:cnk:koditex`, along with scripts for generating new texts (part of another project).

The resulting text was trimmed to begin and end with complete sentences while maintaining approximately 100 words. It was also cleaned of various formatting characters with standardized quotation marks. The second part of the original human-written text underwent identical trimming and cleaning. This process created 672 pairs of topically and stylistically comparable text chunks. These modifications were performed using the script `scripts/sampling/sample_texts.py`, with results stored in `data/koditex_chat_gpt-4o__0.tsv`. From these pairs, one was randomly selected for each experimental trial.

From this file, we manually selected 3 text chunk pairs and attached control questions to determine if participants remained attentive throughout the experiment. These questions were intentionally simple, designed to test attention rather than comprehension ability. The questions are available in `data/control_koditex_chat_gpt-4o__0.tsv`.

5.1 Stylometric Vectors

Given the corpus's diversity in genre and style, we were interested in how a text's style affected participant responses, particularly when the style shifted. We employed Cvrček's stylometric analysis ([2]), based on Biber's analysis ([1]). First, numerous stylometric features were measured for each text, calculating the position of each text chunk (both human and AI-generated) in an eight-dimensional space. Since Cvrček's original model is based on factor analysis, each dimension has a specific interpretation, see table 1.

Table 1: Descriptions and explanations of GLS variables

Variable	Description	Explanation
GLS1	dynamic (+) × static (-)	verbal/clausal × nominal/phrasal constructions
GLS2	spontaneous (+) × prepared (-)	hit-and-miss redundant coding × carefully worded formulations
GLS3	higher (+) × lower (-) level of cohesion	propensity to use connecting devices and means of intratextual reference
GLS4	polythematic (+) × monothematic (-)	lexically rich × repetitive texts
GLS5	higher (+) × lower (-) amount of addressee coding	explicit references to communication partners
GLS6	general (+) × particular (-)	description of general qualities × discussion of particular referents
GLS7	prospective (+) × retrospective (-)	present and future tense, non-narrative × past tense, narrative
GLS8	attitudinal (+) × factual (-)	degree of explicit epistemic certainty, higher × lower amount of hedging

6 Experiment Settings and Demographics

6.1 Pilot

The pilot study was conducted during the Open House Day at the Faculty of Arts, Charles University, Prague (January 11, 2025), with additional participants recruited later. The authors approached high school students, their parents, and other Open House visitors, inviting them to participate in the experiment using their own devices, tablets, or laptops provided by the researchers. After each session, one of the authors conducted a brief interview with the participant to gather feedback on their experience and the technical aspects of the experiment.

Following the pilot, several minor details were adjusted, but the overall experimental design remained unchanged. Data from the pilot study were not used in the main analysis.

6.2 LABELS Participant Pool

The main sample was primarily recruited from the participant pool managed by the LABELS psycholinguistic laboratory (we express our gratitude to Jan Chromý for providing access to this pool and assisting with administration). The pool consists of volunteers interested in participating in linguistic experiments and students who were offered course credits for their participation. Data collection began on February 25, 2025.

The dataset also includes several participants from the general public who responded to Facebook posts by Jiří Milička (March 3, 2025) and Eva Pospíšilová (March 4, 2025), accounting for approximately 22 participants. Data collection concluded on March 14, 2025.

The complete sample comprised 291 participants. However, 33 participants were excluded for failing to answer all attention-check questions correctly (the experiment was demanding, and some students participating for course credits showed insufficient attention). An additional 3 participants were excluded

for not being native Czech speakers and one for low age (under 18 years old). The final sample consist of 254 participants.

7 Data Preparation

Data were first converted to a flat format (one trial per row, with each row containing trial conditions and all metadata and demographic information about the participant). This was accomplished using the script `flatten.py`, producing the file `data/LABELS_flat.tsv`.

This dataset was further processed using the script `process.py`, with results saved to `LABELS_processed.tsv`.

First, participants who did not correctly answer all attention-check questions were removed from the sample, as their attention throughout the experiment could not be guaranteed. These questions were deliberately simple; failure to answer them correctly indicated that participants had not read the texts rather than inability to comprehend them.

Next, participants who were not native Czech speakers were excluded.

Texts related to control questions (at positions 3, 13, and 18) were removed from the dataset as they were identical for all participants.

Perhaps most importantly, we added the target variables: "Is correct" (1 if the participant correctly identified which of the two texts was created by artificial intelligence, 0 otherwise) and "Correctness" (sum of correct answers per participant).

We also added columns with vectors related to stylometric text properties, prepared using Cvrček's script ([3]) (loaded from the `multidimcvrcek` folder) — GLS scores (GLS1-GLS8) for human- and AI-generated texts, plus:

- Differences (AI – Human)
- Absolute distances (|Human – AI|)
- Cosine distance between human and AI vectors
- Euclidean distance between human and AI vectors

Reaction times were calculated, specifically reaction time in milliseconds and its $\log_2$ transformation (the logarithmic variant was used in subsequent analyses).

For operational purposes, some columns were renamed:

- *Is learner* to *got_feedback*
- *Demographics: ai_creativity* to *Demographics: ai_lacks_creativity*
- *Demographics: ai_understanding* to *Demographics: ai_lacks_understanding*

Several variables were also reduced to decrease the number of factors for modeling (creating new columns while preserving originals): The merged categories and ordinal versions were specifically created to reduce the number of categories for mixed modeling analysis, while the original columns remain available for other analyses requiring the full granularity of the data.

- Gender: *Demographics: gender merged* — combines *other* and *prefer_not* into a single *other* category
- Reading disorder: *Demographics: reading_disorder_merged* — combines *false* and *unknown* into *false*, as people who had not been diagnosed tended to answer that they didn't know or that they didn't have a reading disorder.

We also added ordinal numbers to properly sort variables related to how frequently people interact with AI:

Demographics: ai_usage was recoded with ordered prefixes:

- *never* → *0_never*
- *less_than_monthly* → *1_less_monthly*
- *monthly* → *2_monthly*

- *several_monthly* → *3_several_monthly*
- *weekly* → *4_weekly*
- *several_weekly* → *5_several_weekly*
- *daily* → *6_daily*
- *unknown* → *0_unknown*

These numbers were then isolated in a separate column to create an ordinal variable. Similarly, an ordinal variable was created from *Education*, starting from high school (number 1) to doctorate (number 6). Completed and incomplete studies were combined (the vast majority of participants were students).

Finally, a column was created for the variable *Demographics: ai_negative_attitude*, which indicates how negative a participant's attitude toward AI was. This variable was derived from MDA of questionnaire responses, which will be discussed in the following section and was loaded from the file `PCA/participant_position.tsv`.

8 PCA of AI Attitude Questionnaire

Before beginning the experiment, participants received a set of questions about their attitudes toward artificial intelligence, which they answered on a Likert scale. As this increased the number of fixed effects, we decided to reduce dimensionality to the most important dimension.

While the Likert scale is not parametric, and more appropriate methods exist for processing such data (e.g., CATPCA), we opted for simple PCA to obtain the most straightforward interpretation of eigenvectors for individual questions.

The questions were as follows:

Demographics: ai_improve: AI can improve the quality of our daily life. *Demographics: ai_risk*: AI poses a risk to human safety. *Demographics: ai_quality*: Automatically generated texts can be as good as human-written texts. *Demographics: ai_lacks_creativity*: Automatically generated texts lack human creativity and personal style. *Demographics: ai_lacks_understanding*: AI is not capable of true understanding like humans. *Demographics: ai_grammar*: AI produces grammatically correct texts. *Demographics: ai_avoidance*: I prefer to avoid using AI when writing important texts. *Demographics: ai_dumbing*: I am concerned that people are becoming dumber because of AI. *Demographics: ai_fascination*: I am fascinated by what AI can do in text generation.

PCA was performed using the script `analysis/scripts/CATPCA.py`, which created both the aforementioned PCA (in the `scripts/analysis/PCA` folder) and CATPC (in the `scripts/analysis/PCA` folder, which is more difficult to interpret and therefore not discussed here, though you are welcome to examine it).

The first two PC dimensions have eigenvalues of 7.28 and 3.60, after which the values drop rapidly (the next has 2.51), so we focused on the first two dimensions. Figure 1 shows that participants are well distributed in the space without dramatic outliers. The eigenvectors in the same figure explain the first dimension as a scale between positive and negative attitudes toward AI. Participants positioned on the right of the scale tend to believe that AI is not very capable, are not enthusiastic about it, do not use it for important tasks, and simultaneously consider it radically different from the human mind, as they believe it cannot be creative and is incapable of true understanding of what it does.

The second dimension relates to concerns. Those who scored high on this dimension do not underestimate AI—on the contrary, they are fascinated by it and believe it can improve the quality of human life, but simultaneously fear its impacts.

9 List of Variables and Results

All demographic variables (i.e., variables relating to participants) were processed using the script `scripts/analysis/dependencies_demographics.py`, which created not only graphs showing the dependence of target variables on individual parameters but also distributions of all variables in the `scripts/analysis/Demographics` folder. All graphs include 95% confidence intervals derived from bootstrap resampling of individual participants. All other variables were similarly processed using `scripts/analysis/dependencies.py`, which created dependency graphs (but not distributions) in the `scripts/analysis/Dependencies` folder. Here too, all categorical and integer variables have 95% confidence intervals derived from

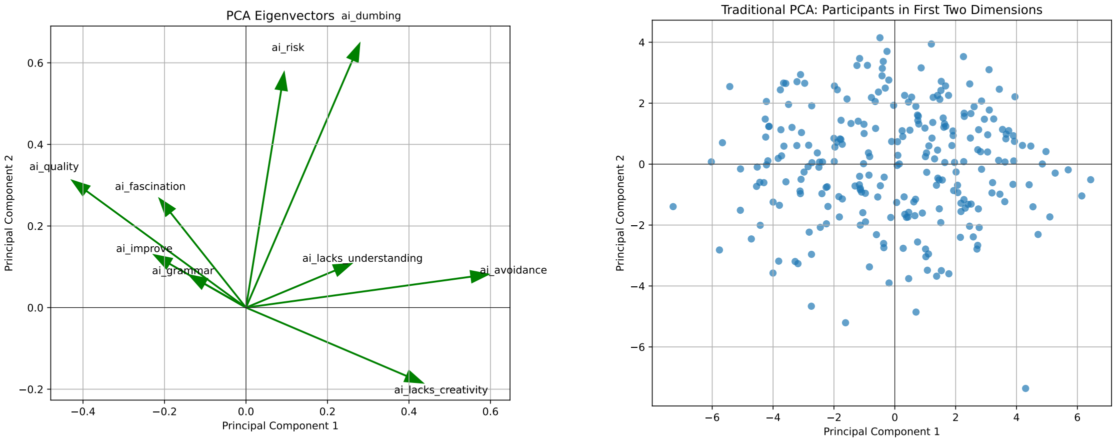

Figure 1: First two dimension

bootstrap resampling of individual participants (rather than individual trials, as these are not independent of each other) for all x-values with more than 5 occurrences. Additionally, thinner lines representing 100 bootstrap samples (*bootstrap replicates* or *bootstrap traces*) were included. For continuous variables, these lines were created by fitting LOWESS curves to the bootstrapped data. Outliers were removed from the figures using the 2 × IQR rule.

These scripts produced a large number of graphs (essentially for all variable combinations); here we present only those that are most relevant.

9.1 Dividing Participants into Feedback / no feedback groups (*got_feedback*)

At the beginning of the experiment, each participant was assigned with a 50% probability to a group that either received or did not receive feedback after each response. Participants with feedback immediately learned whether they answered correctly and could therefore learn how to recognize AI-generated texts. Since the influence of this variable is a central question of this study, all analyses separate results for participants with and without feedback.

9.2 Correctness of the Answer (*is_correct*)

Correctness of the Answer is a binary variable that indicates whether a participant correctly assigned which text was AI-generated and which was human-written in each trial (1 for correct, 0 for incorrect). The distribution of this variable can be seen in Figure 2, which is also included in the article itself.

This graph (like Figure 3) was created using the script `scripts/analysis/overall.py`, with graphs located in the folder `scripts/analysis/Overallridgeline`.

Here we observe a substantial difference between feedback and no-feedback groups, with the no-feedback group showing considerably poorer results at approximately 55%, with a confidence interval below 59%, while the feedback group achieved around 65%, with the lower confidence interval limit not falling below 62%.

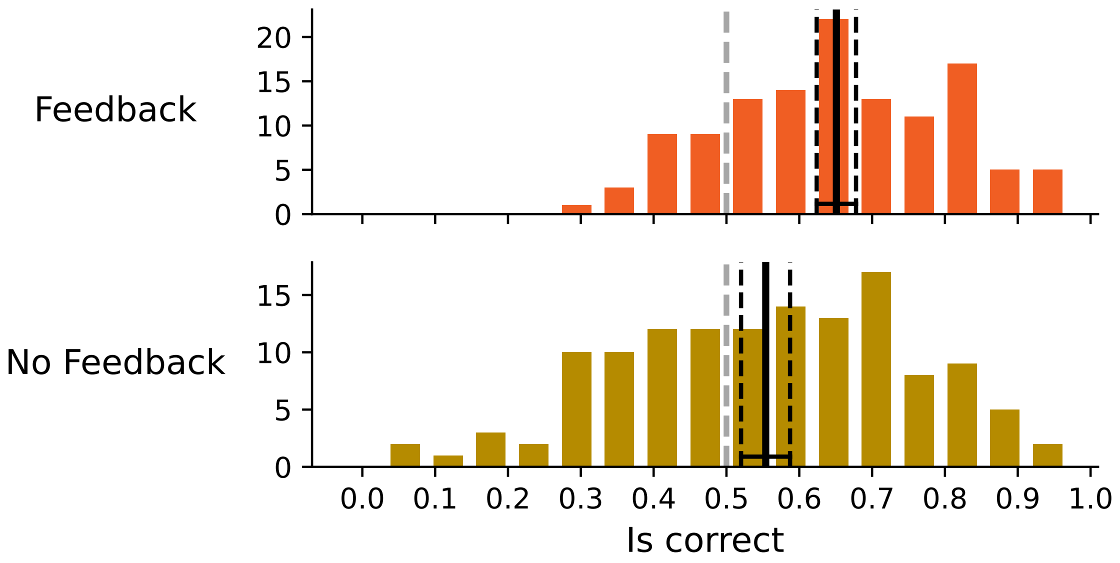

Figure 2: The distribution of the correctness of the answers.

9.3 Readability (*more_readable_is_human*)

For each pair of texts, participants were asked which text they found more readable. This variable indicates whether the participant thought the human-written text was the more readable one of the pair. The distribution of this variable can be seen in Figure 3.

As evident, participants systematically considered AI-written texts more readable (only approximately 30% of human-produced texts were considered more readable than AI-generated ones), with no significant difference between feedback and no-feedback groups.

Participants also more easily identified texts they considered more readable and this effect was even larger for no-feedback group (Figure 4).

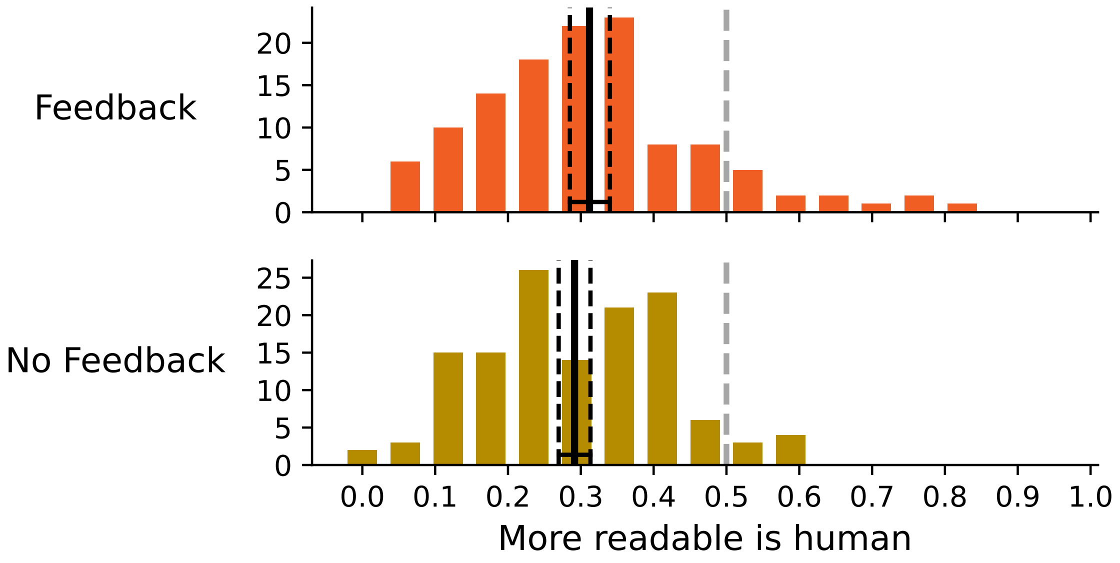

Figure 3: The distribution of the readability.

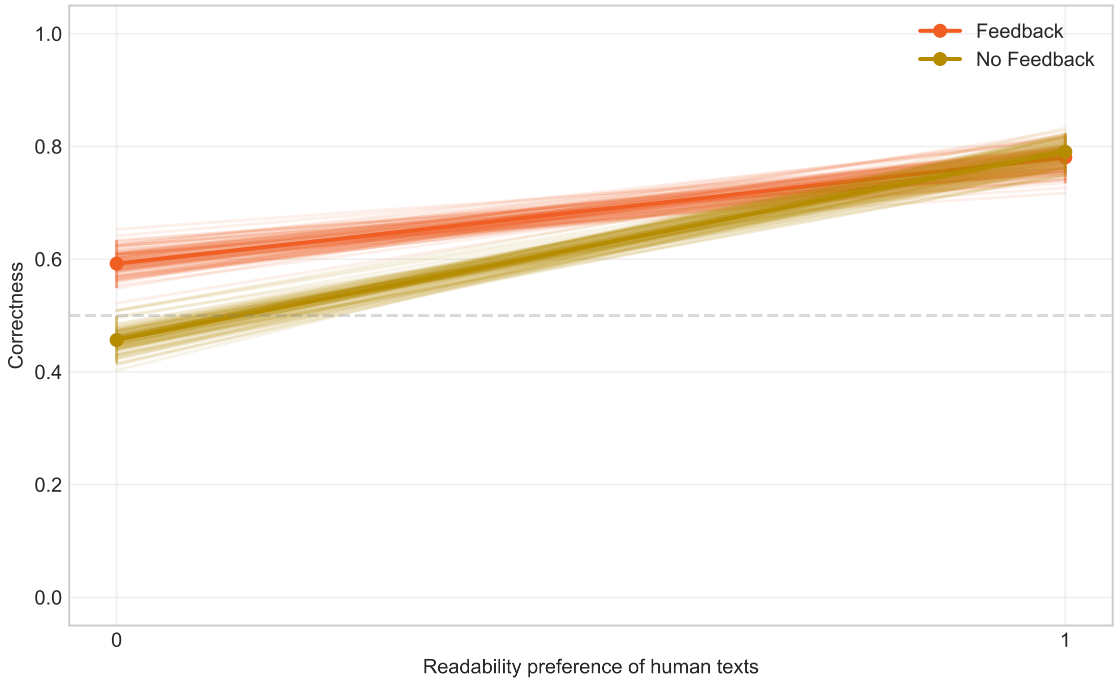

Figure 4: The distribution of the readability.

9.4 Confidence

For each pair of texts, participants had to express how confident they were in their answer on a scale of 1–7 (i.e., how certain they were that they correctly identified which text was human-written and which was AI-generated).

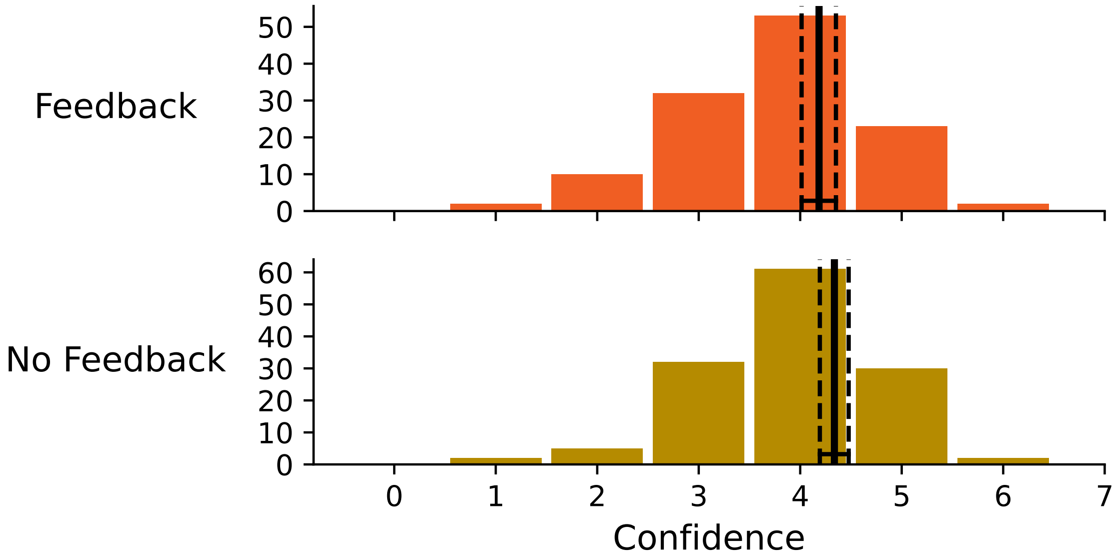

Figure 5: The distribution of the correctness of the answers.

As shown in Figure 6, at low confidence levels, there was little difference whether a participant received feedback or not. However, from approximately level 5 and above, where participants with feedback were confident, they indeed showed better correctness. The greatest difference appears at confidence level 7, where people without feedback were overconfident and made the most errors precisely when they were most certain (even below the chance level of 0.5), while those with feedback were justifiably confident,

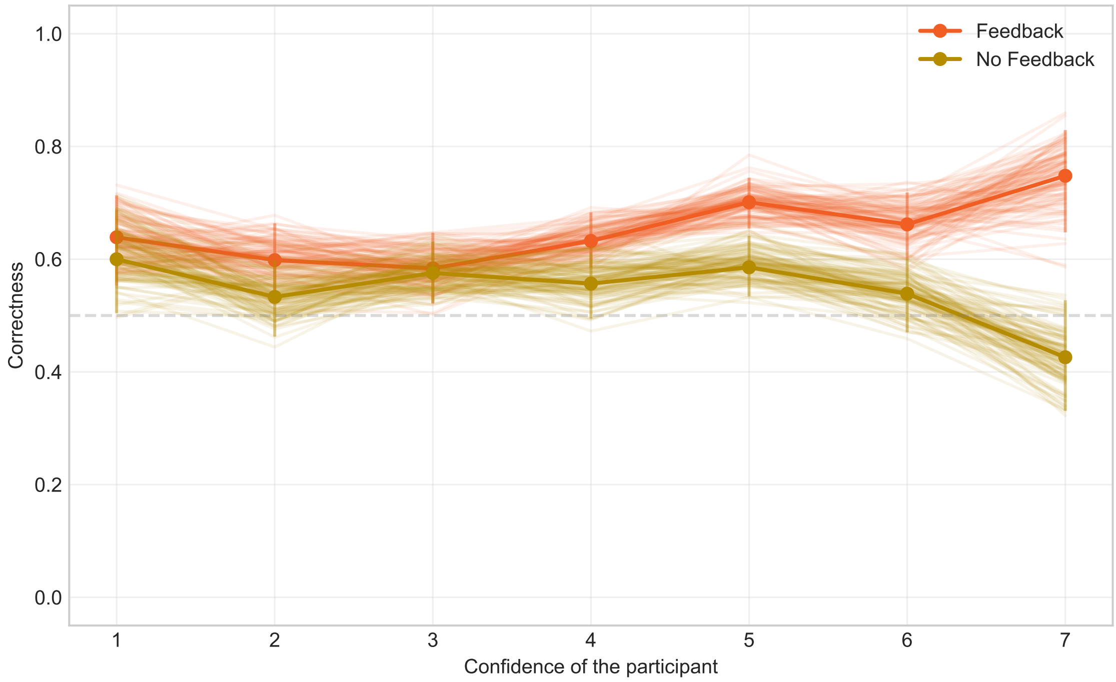

Figure 6: Dependence of Confidence of the answer on Correctness. Bootstrapped 95% confidence intervals.

achieving decisively better results.

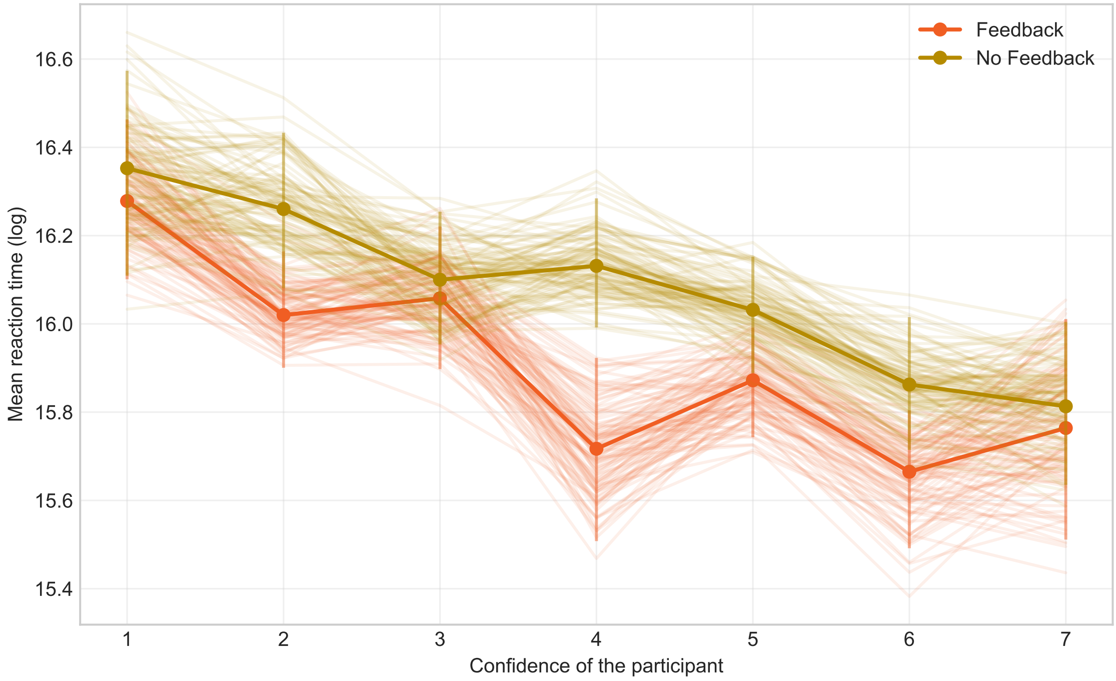

Figure 7: Dependence of Confidence of the answer on Reaction time (log). Bootstrapped 95% confidence intervals.

We can also examine the relationship with reaction time, where questions with greater confidence were answered more quickly (in both groups), which is not particularly surprising (Figure 7).

We can also consider confidence as a dependent variable, especially examining how it relates to trial order. We might expect some development, particularly in the feedback group, but the chart appears

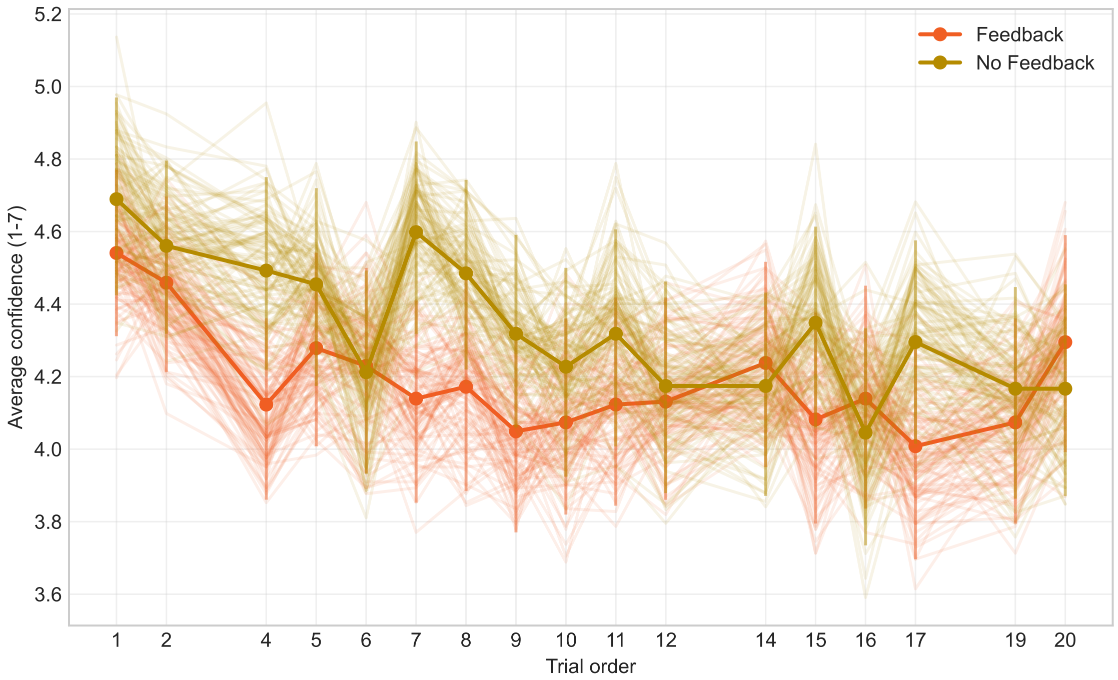

Figure 8: Dependence of Trial order of the answer on Confidence. Bootstrapped 95% confidence intervals.

rather chaotic. The only systematic observation is that people with feedback show lower confidence (as they know what they do not know), but the difference is not significant (Figure 8).

9.5 Trial Order (*id*)

Another variable that is extremely important for our main hypothesis. If we are asking whether the group that received feedback differs from the one that did not, we assume that those with feedback were able to learn. Their abilities should therefore improve over time (Figure 9).

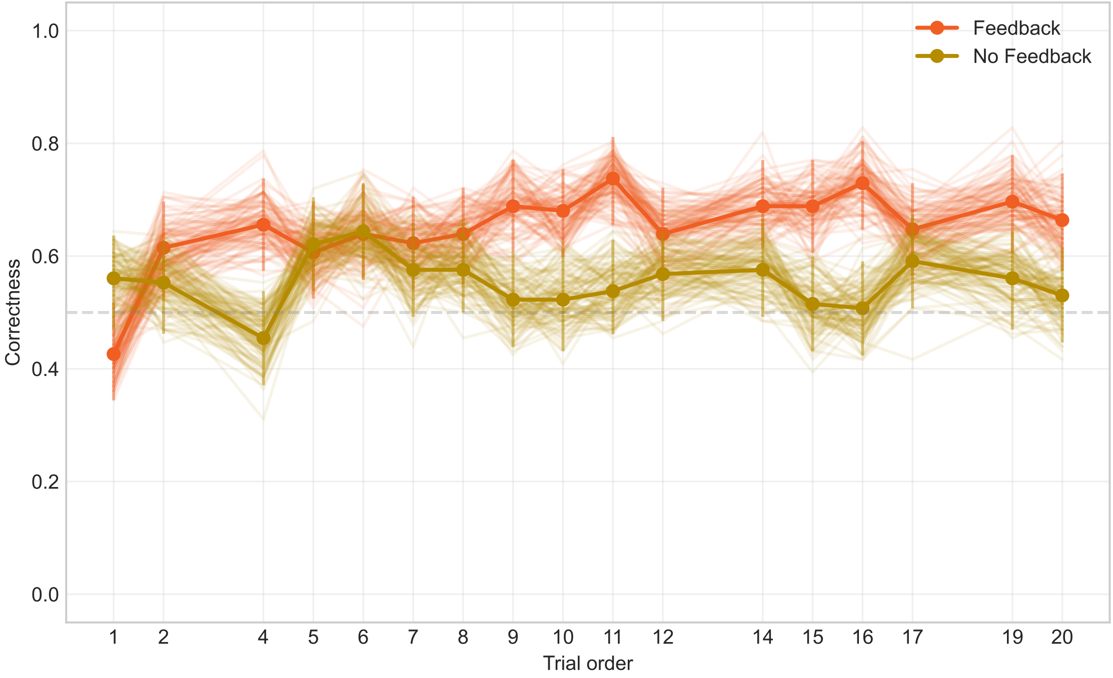

Figure 9: Dependence of Trial order of the answer on Correctness. Bootstrapped 95% confidence intervals.

9.6 Logarithm of Reaction Time (*Reaction_time_log*)

This is typically used as a target variable. In this context, we can examine how this variable differs depending on trial order, for instance, whether people spent more time on each task at the beginning than at the end when they might have been tired. The result can be seen in Figure 10.

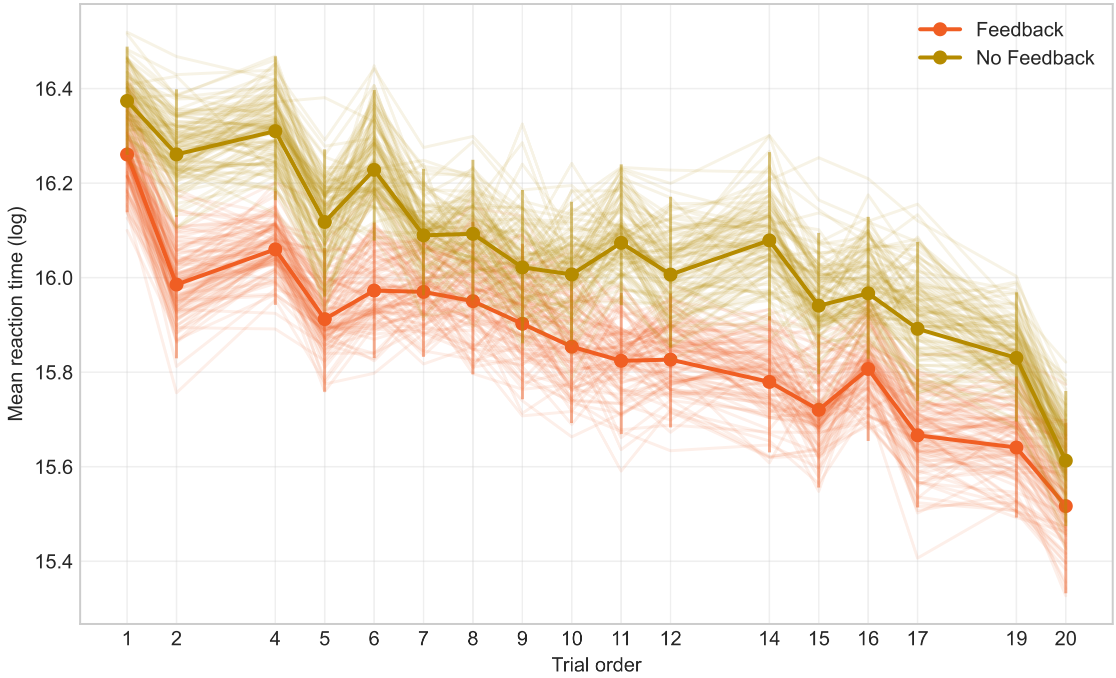

Figure 10: Dependence of Trial order of the answer on Reaction time (log). Bootstrapped 95% confidence intervals.

We can also consider it as an explanatory variable — that is, whether participants who spent more time reading and analyzing had more accurate answers than those who responded more intuitively. This is shown in Figure 11.

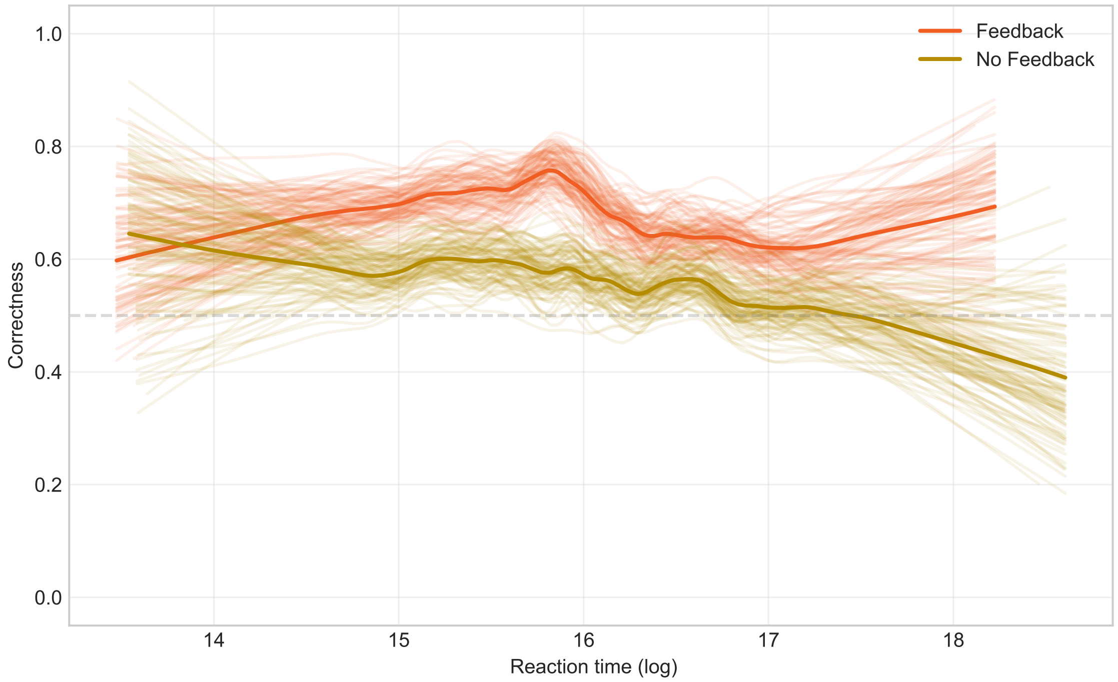

Figure 11: Dependence of Reaction time (log) of the answer on Correctness. Bootstrapped 95% confidence intervals.

9.7 Euclidean Distance of Stylometric Vectors (*GLS_Euclidean_distance*)

Another crucial variable—we utilize the fact that both the human-generated text chunk and the AI-generated text chunk are continuations of the same text, so they should be comparable. It should therefore hold that texts that are stylistically more similar should be harder to distinguish, as this indicates that the AI-generated text chunk is a more credible continuation of the given text. The results can be seen in Figure 12.

Let us now examine all dimensions one by one:

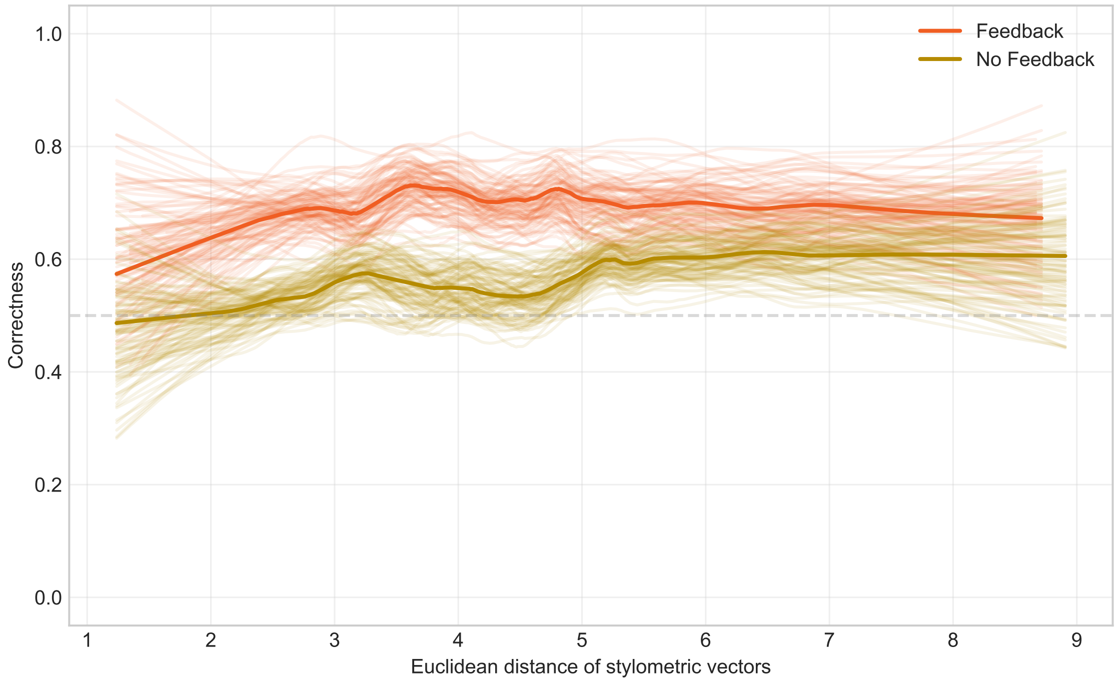

Figure 12: Dependence of Euclidean distance of stylometric vectors of the answer on Correctness. Bootstrapped 95% confidence intervals.

9.7.1 Dynamic (+) × Static (-) (*GLS1*)

This dimension expresses the tendency of the text to use verbal/clausal versus nominal/phrasal constructions.

The dependence of correctness on the position of the human-written text along this dimension is shown in Figure 13. The dependence of correctness on the position of the AI-generated text is in Figure 14. The dependence of correctness on the difference between them is in Figure 15, and the dependence on the distance between them (i.e., the absolute value of the difference) is in Figure 16.

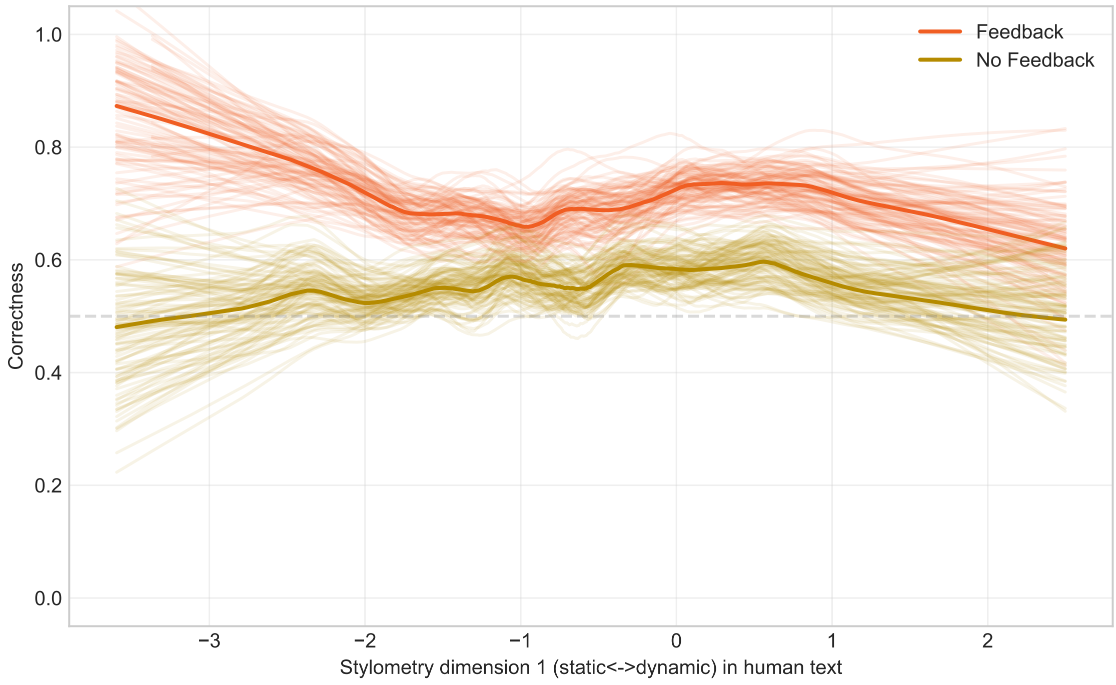

Figure 13: Dependence of Position of human text on 1st stylometric dimension of the answer on Correctness. Bootstrapped 95% confidence intervals.

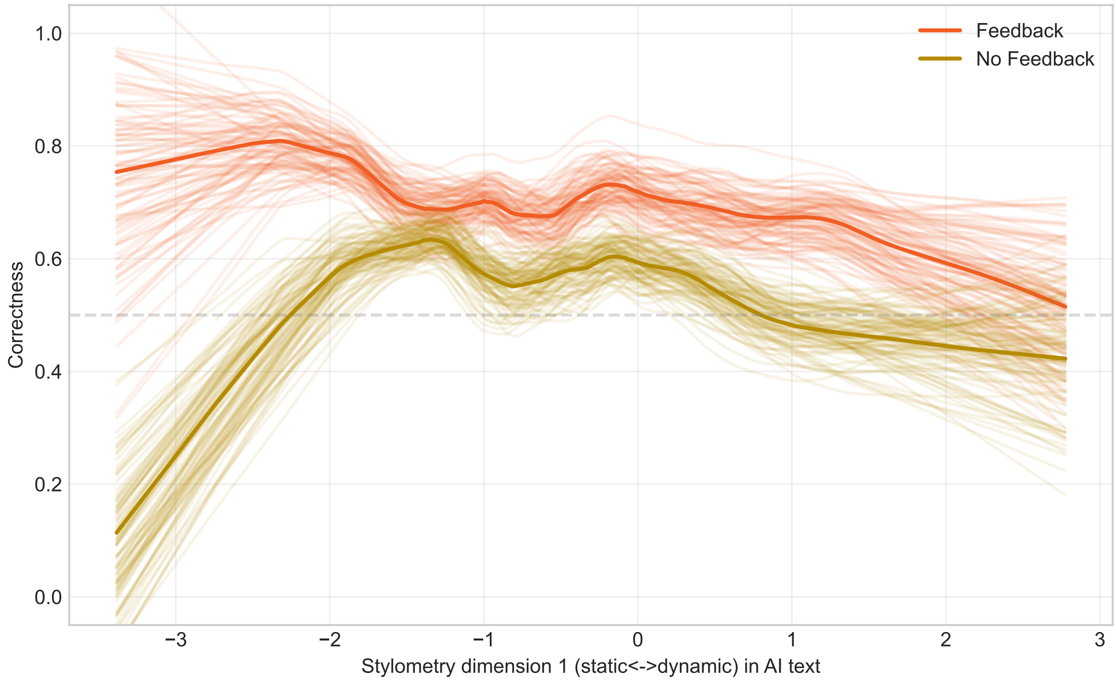

Figure 14: Dependence of Position of AI text on 1st stylometric dimension of the answer on Correctness. Bootstrapped 95% confidence intervals.

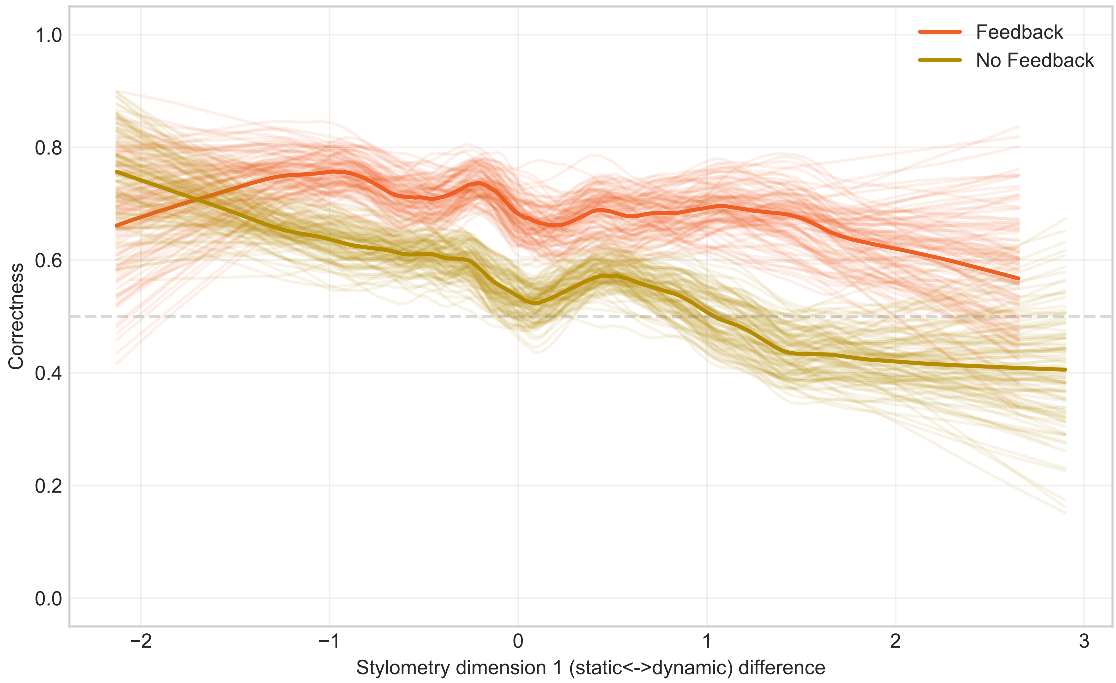

Figure 15: Dependence of Difference between AI and human text on 1st stylometric dimension of the answer on Correctness. Bootstrapped 95% confidence intervals.

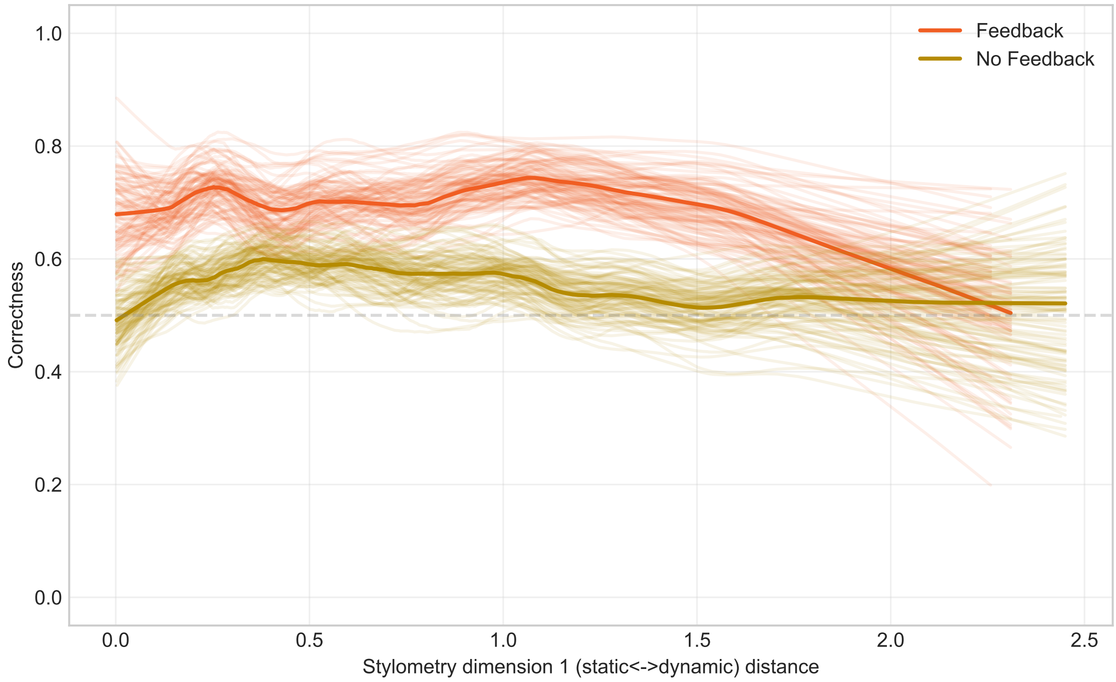

Figure 16: Dependence of Distance between AI and human text on 1st stylometric dimension of the answer on Correctness. Bootstrapped 95% confidence intervals.

9.7.2 Spontaneous (+) × Prepared (-) (*GLS2*)

This dimension expresses the tendency of the text to use hit-and-miss redundant coding versus carefully worded formulations.

The dependence of correctness on the position of the human-written text along this dimension is shown in Figure 17. The dependence of correctness on the position of the AI-generated text is in Figure 18. The dependence of correctness on the difference between them is in Figure 19, and the dependence on the distance between them (i.e., the absolute value of the difference) is in Figure 20.

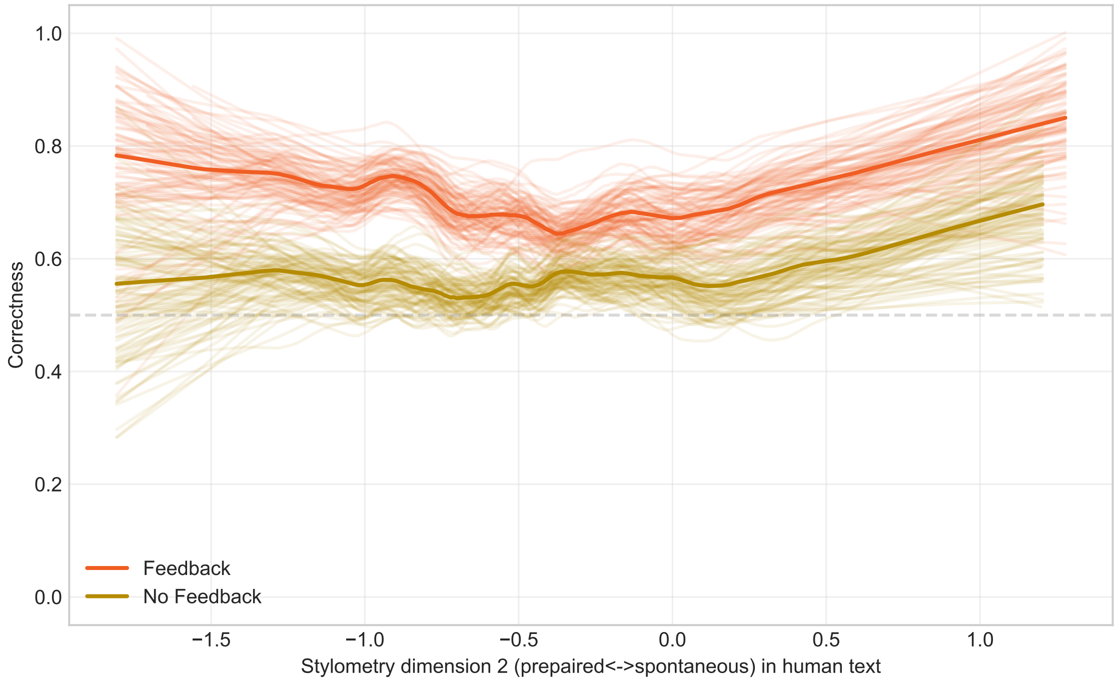

Figure 17: Dependence of Position of human text on 2nd stylometric dimension of the answer on Correctness. Bootstrapped 95% confidence intervals.

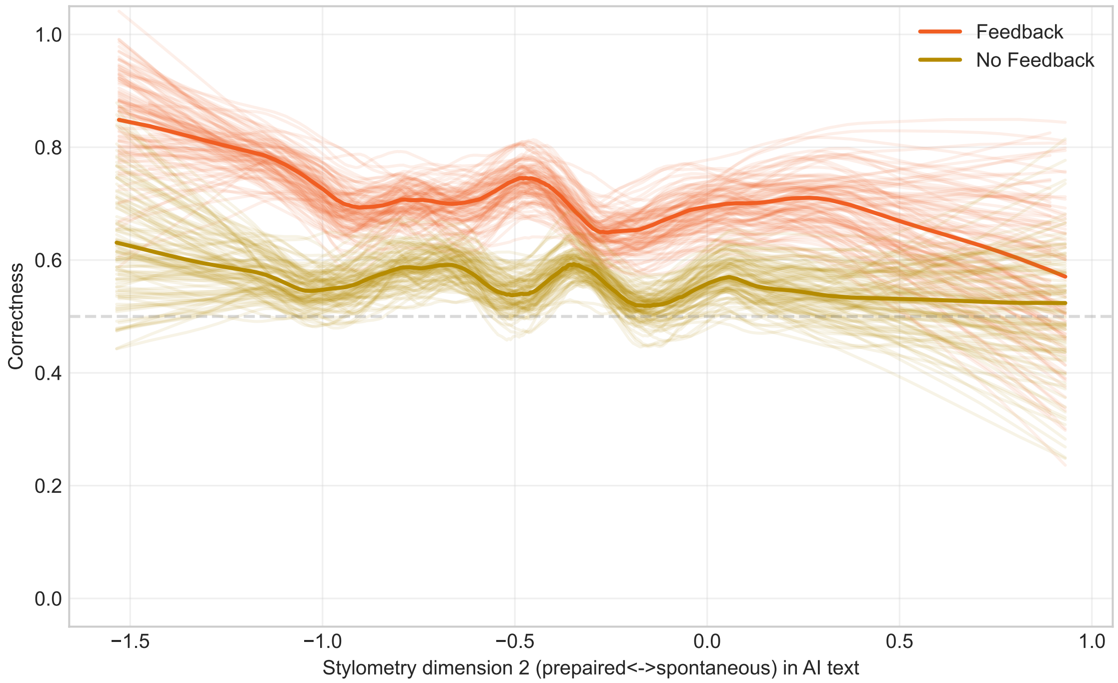

Figure 18: Dependence of Position of AI text on 2nd stylometric dimension of the answer on Correctness. Bootstrapped 95% confidence intervals.

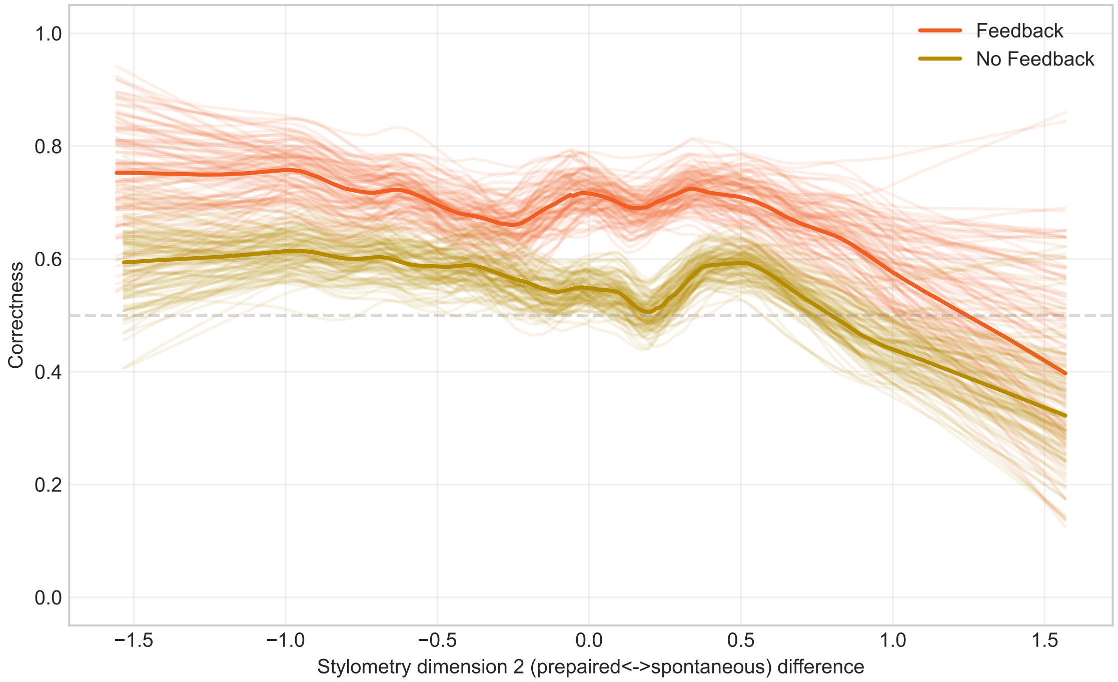

Figure 19: Dependence of Difference between AI and human text on 2nd stylometric dimension of the answer on Correctness. Bootstrapped 95% confidence intervals.

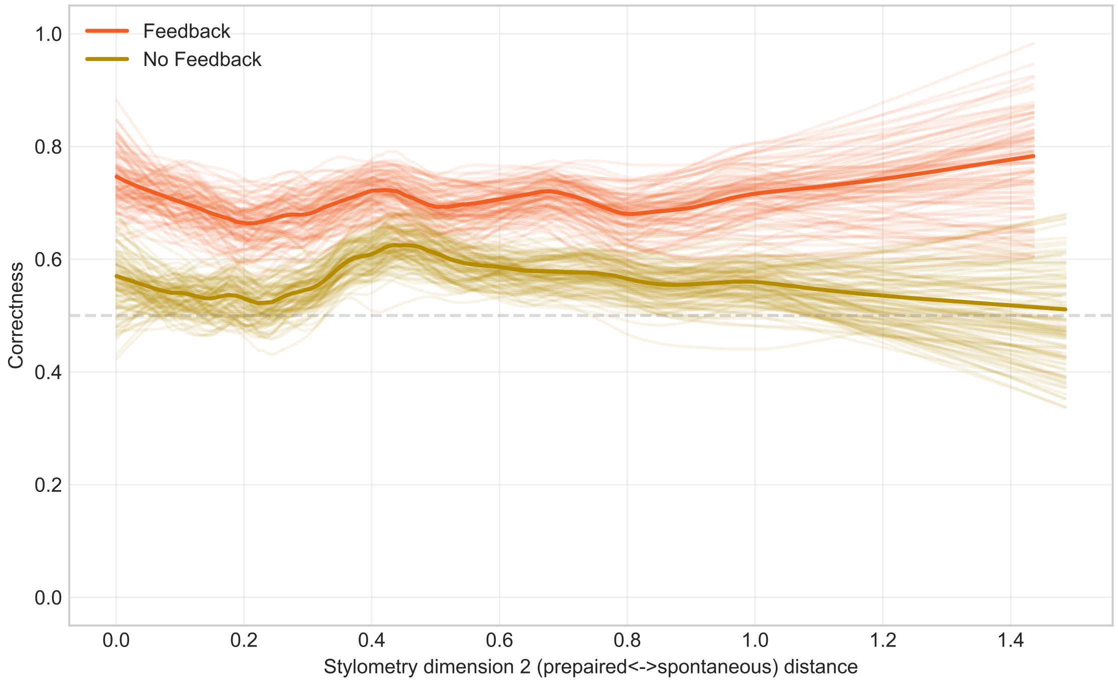

Figure 20: Dependence of Distance between AI and human text on 2nd stylometric dimension of the answer on Correctness. Bootstrapped 95% confidence intervals.

9.7.3 Higher (+) × Lower (-) Level of Cohesion (*GLS3*)

This dimension expresses the tendency of the text to use connecting devices and means of intratextual reference.

The dependence of correctness on the position of the human-written text along this dimension is shown in Figure 21. The dependence of correctness on the position of the AI-generated text is in Figure 22. The dependence of correctness on the difference between them is in Figure 23, and the dependence on the distance between them (i.e., the absolute value of the difference) is in Figure 24.

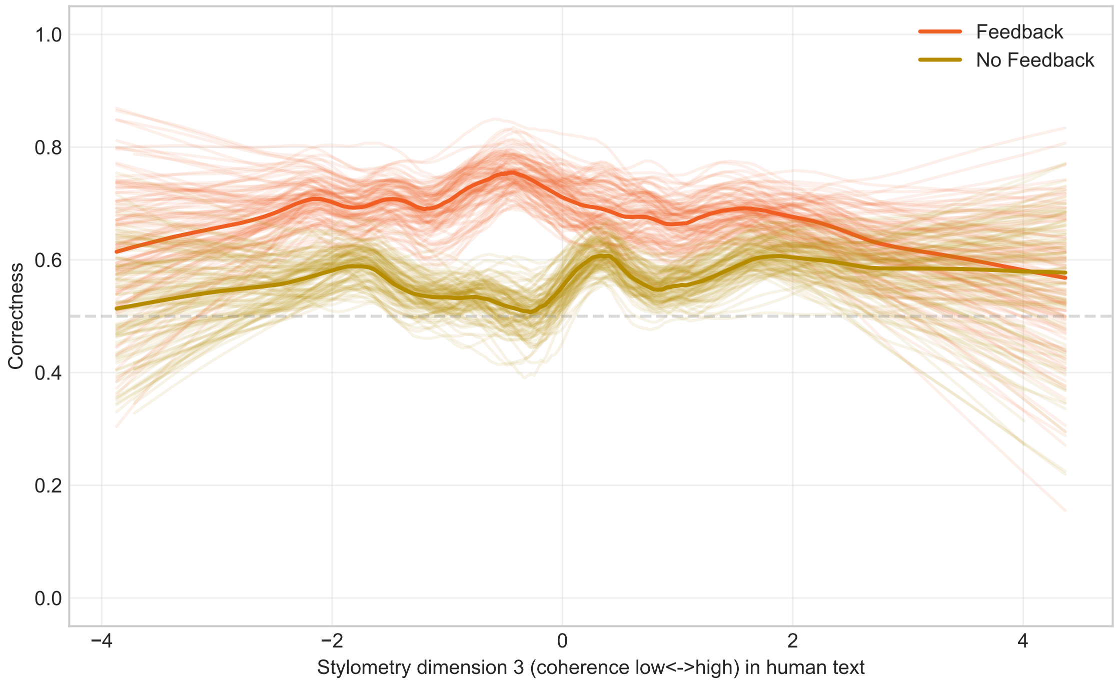

Figure 21: Dependence of Position of human text on 3rd stylometric dimension of the answer on Correctness. Bootstrapped 95% confidence intervals.

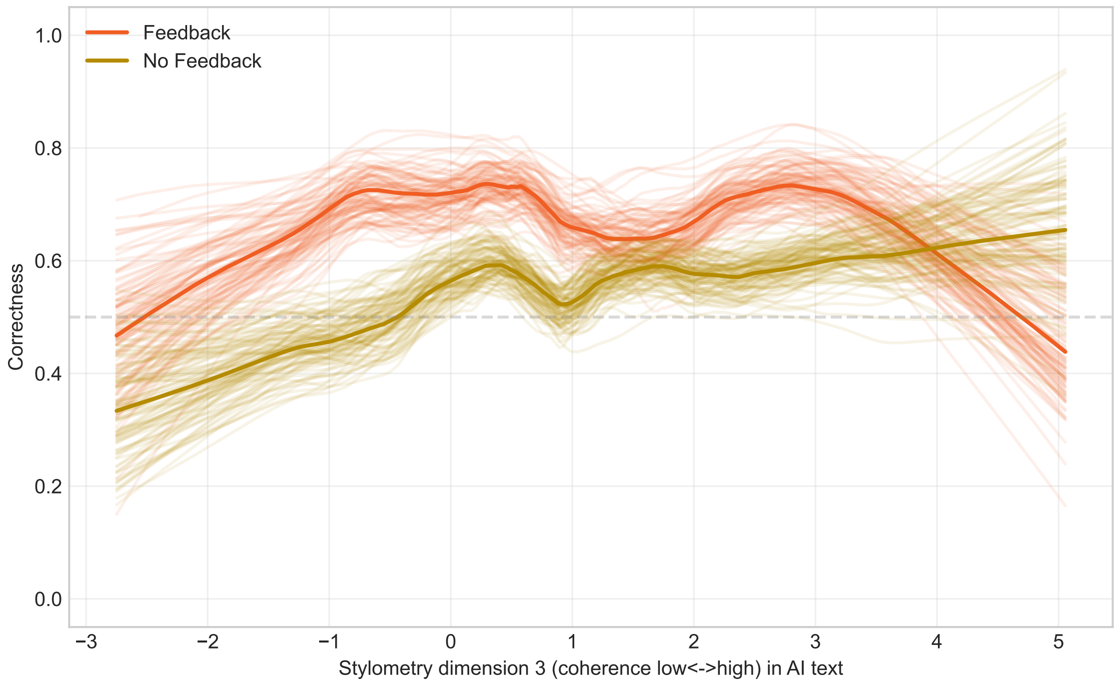

Figure 22: Dependence of Position of AI text on 3rd stylometric dimension of the answer on Correctness. Bootstrapped 95% confidence intervals.

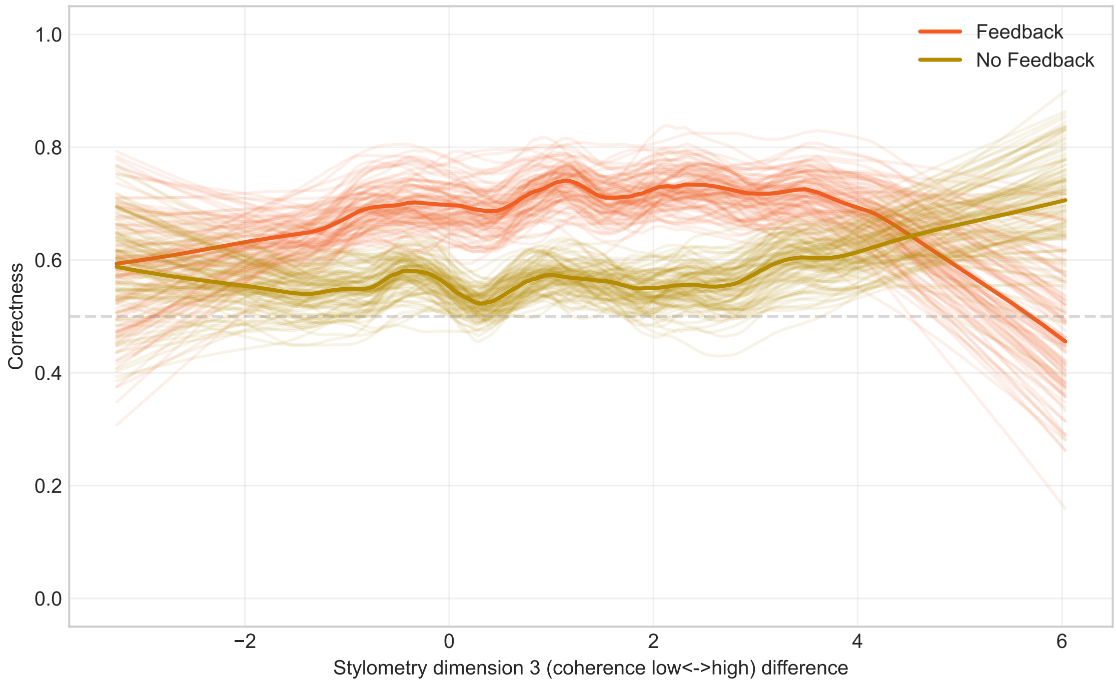

Figure 23: Dependence of Difference between AI and human text on 3rd stylometric dimension of the answer on Correctness. Bootstrapped 95% confidence intervals.

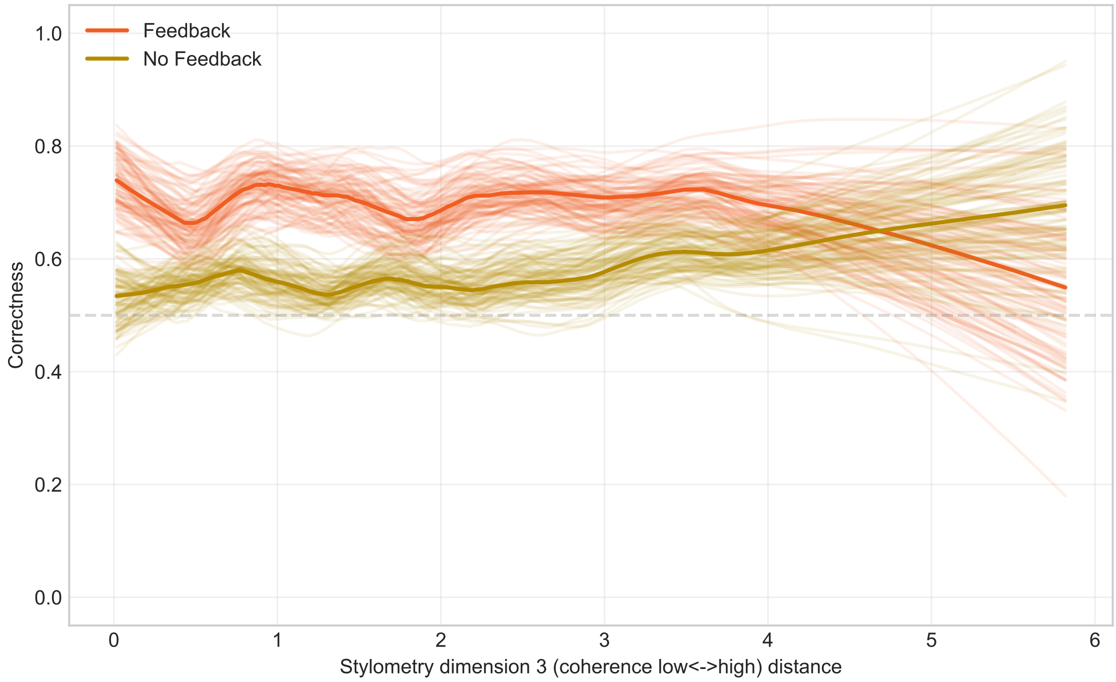

Figure 24: Dependence of Distance between AI and human text on 3rd stylometric dimension of the answer on Correctness. Bootstrapped 95% confidence intervals.

9.7.4 Polythematic (+) × Monothematic (-) (*GLS4*)

This dimension expresses the tendency of the text to be lexically rich versus repetitive.

The dependence of correctness on the position of the human-written text along this dimension is shown in Figure 25. The dependence of correctness on the position of the AI-generated text is in Figure 26. The dependence of correctness on the difference between them is in Figure 27, and the dependence on the distance between them (i.e., the absolute value of the difference) is in Figure 28.

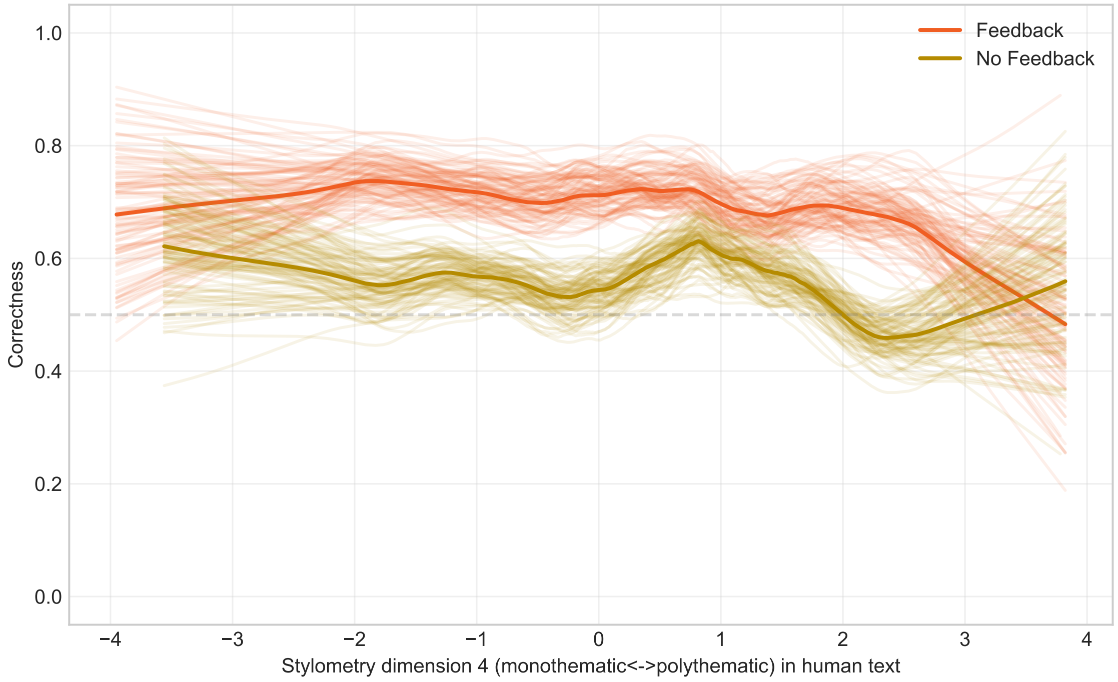

Figure 25: Dependence of Position of human text on 4th stylometric dimension of the answer on Correctness. Bootstrapped 95% confidence intervals.

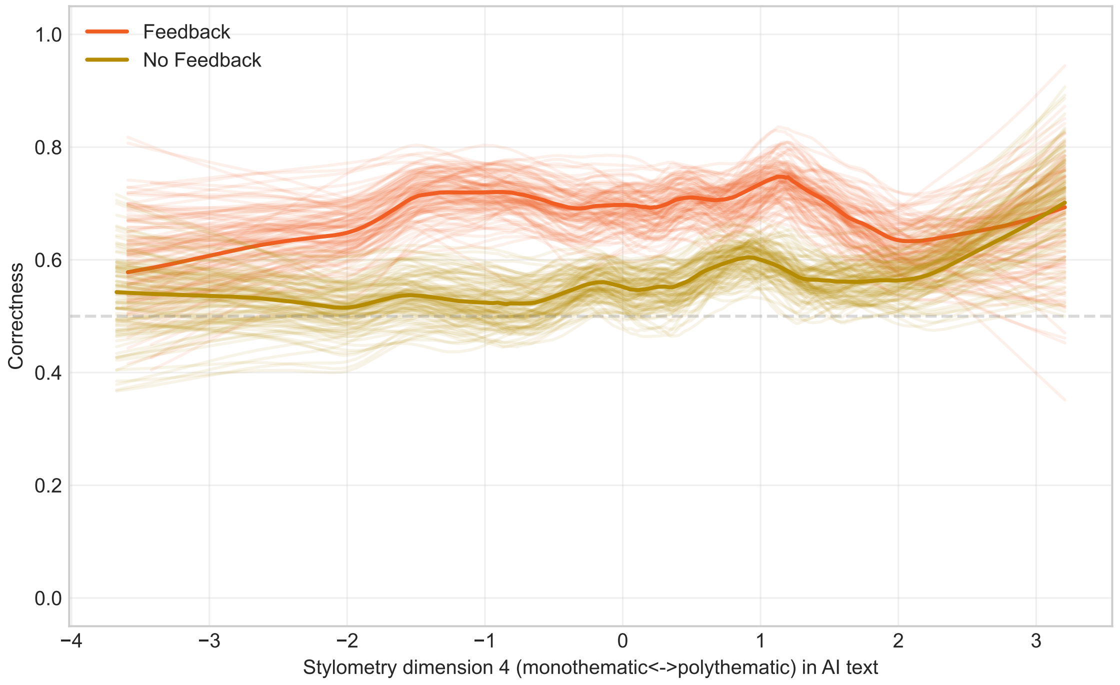

Figure 26: Dependence of Position of AI text on 4th stylometric dimension of the answer on Correctness. Bootstrapped 95% confidence intervals.

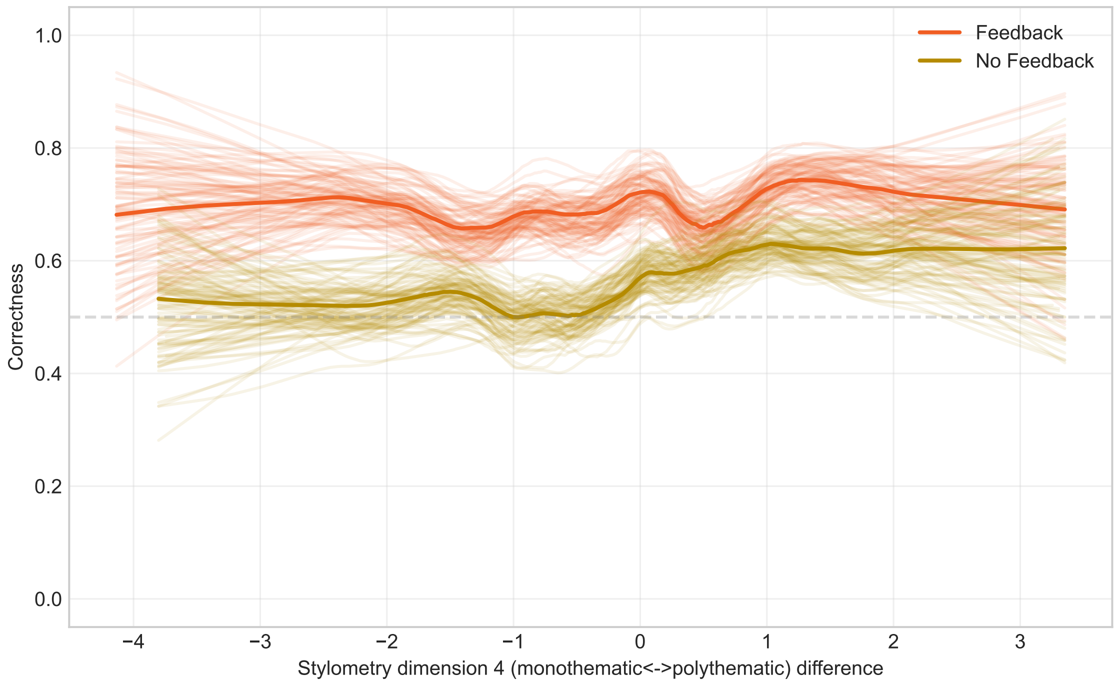

Figure 27: Dependence of Difference between AI and human text on 4th stylometric dimension of the answer on Correctness. Bootstrapped 95% confidence intervals.

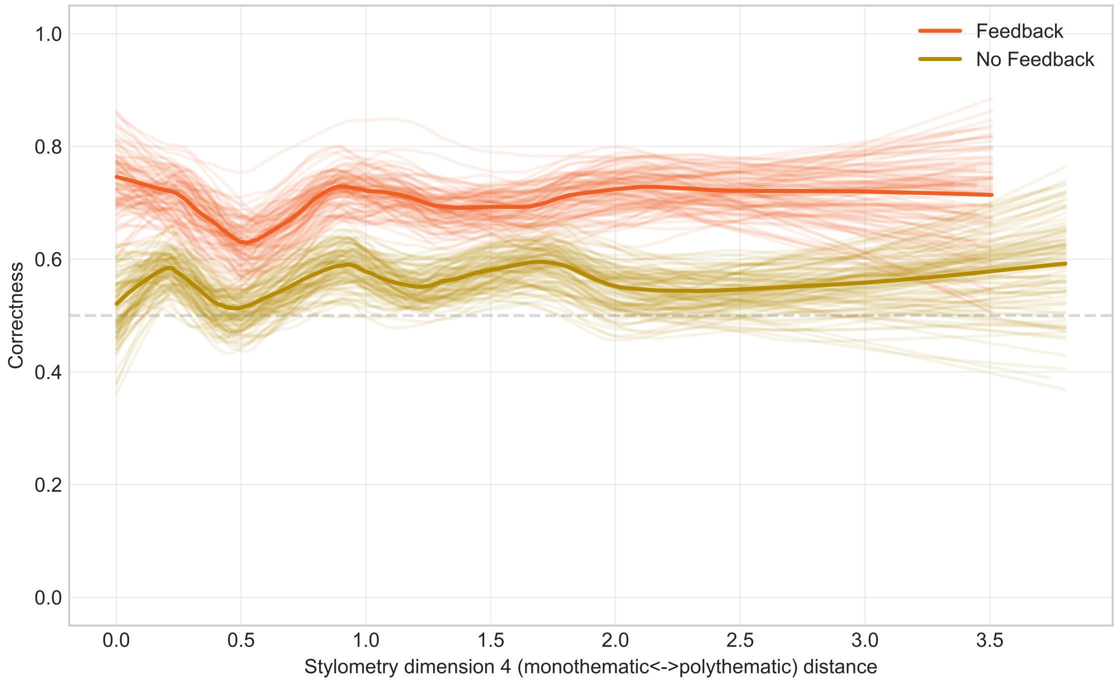

Figure 28: Dependence of Distance between AI and human text on 4th stylometric dimension of the answer on Correctness. Bootstrapped 95% confidence intervals.

9.7.5 Higher (+) × Lower (-) Amount of Addressee Coding (*GLS5*)

This dimension expresses the tendency of the text to include explicit references to communication partners.

The dependence of correctness on the position of the human-written text along this dimension is shown in Figure 29. The dependence of correctness on the position of the AI-generated text is in Figure 30. The dependence of correctness on the difference between them is in Figure 31, and the dependence on the distance between them (i.e., the absolute value of the difference) is in Figure 32.

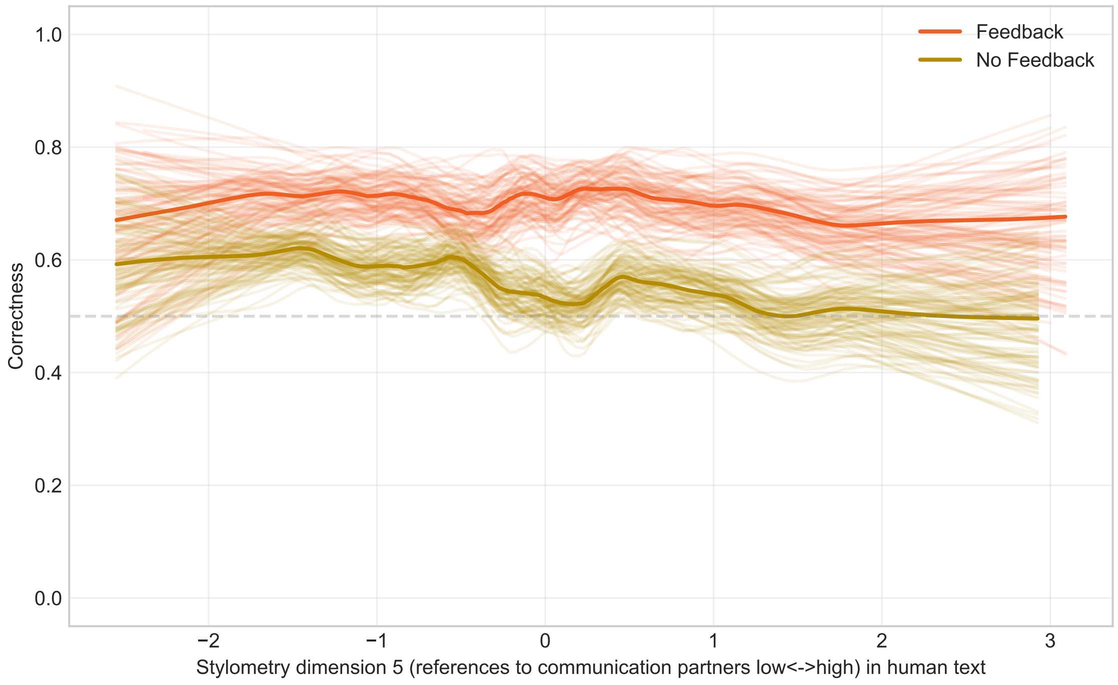

Figure 29: Dependence of Position of human text on 5th stylometric dimension of the answer on Correctness. Bootstrapped 95% confidence intervals.

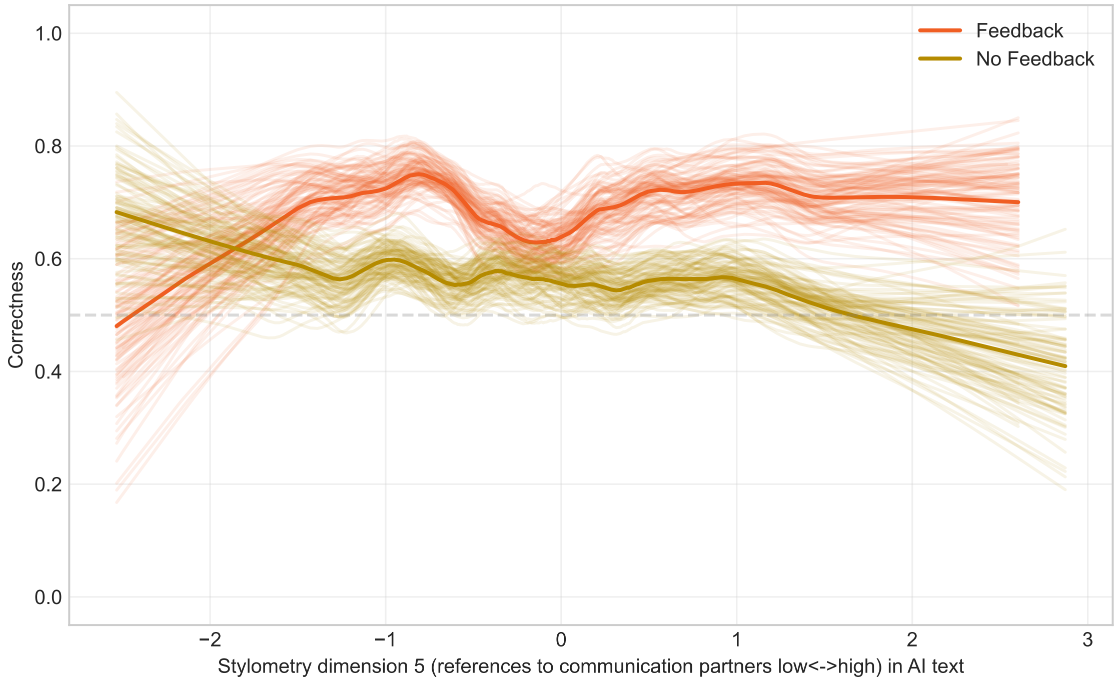

Figure 30: Dependence of Position of AI text on 5th stylometric dimension of the answer on Correctness. Bootstrapped 95% confidence intervals.

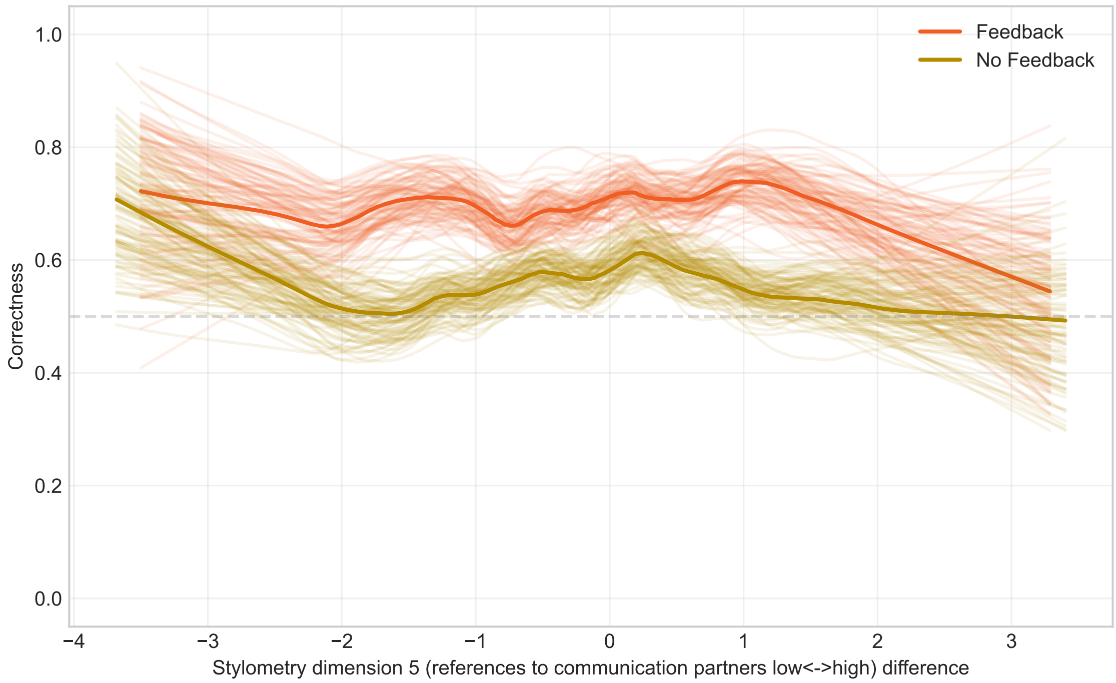

Figure 31: Dependence of Difference between AI and human text on 5th stylometric dimension of the answer on Correctness. Bootstrapped 95% confidence intervals.

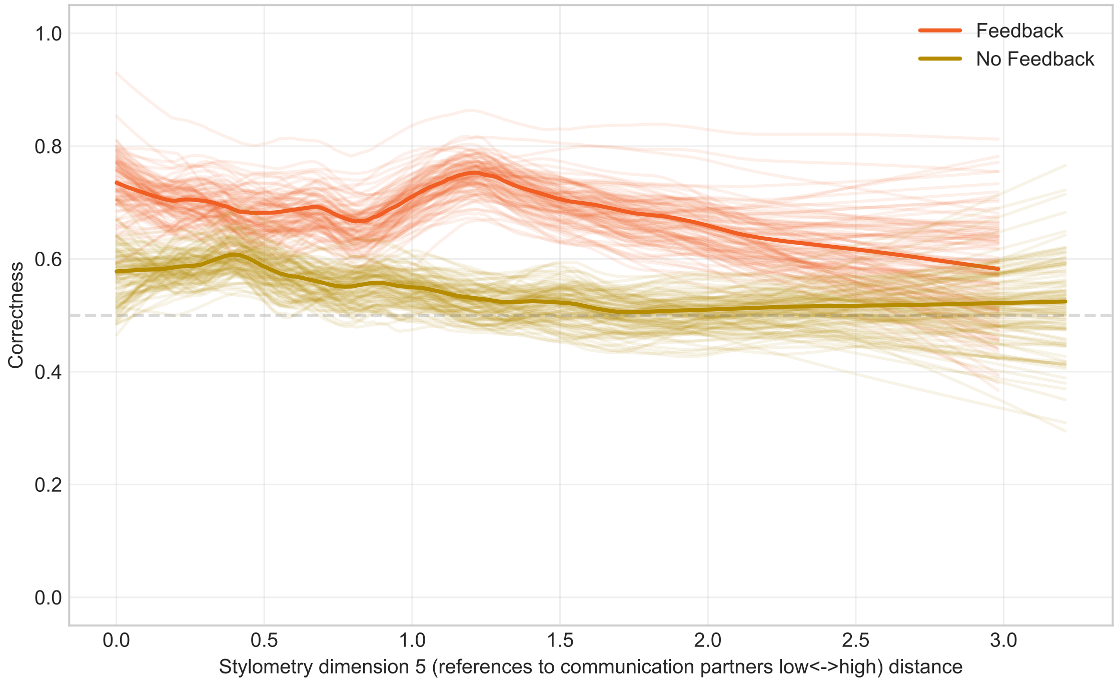

Figure 32: Dependence of Distance between AI and human text on 5th stylometric dimension of the answer on Correctness. Bootstrapped 95% confidence intervals.

9.7.6 General (+) × Particular (-) (*GLS6*)

This dimension expresses the tendency of the text to describe general qualities versus discussing particular referents.

The dependence of correctness on the position of the human-written text along this dimension is shown in Figure 33. The dependence of correctness on the position of the AI-generated text is in Figure 34. The dependence of correctness on the difference between them is in Figure 35, and the dependence on the distance between them (i.e., the absolute value of the difference) is in Figure 36.

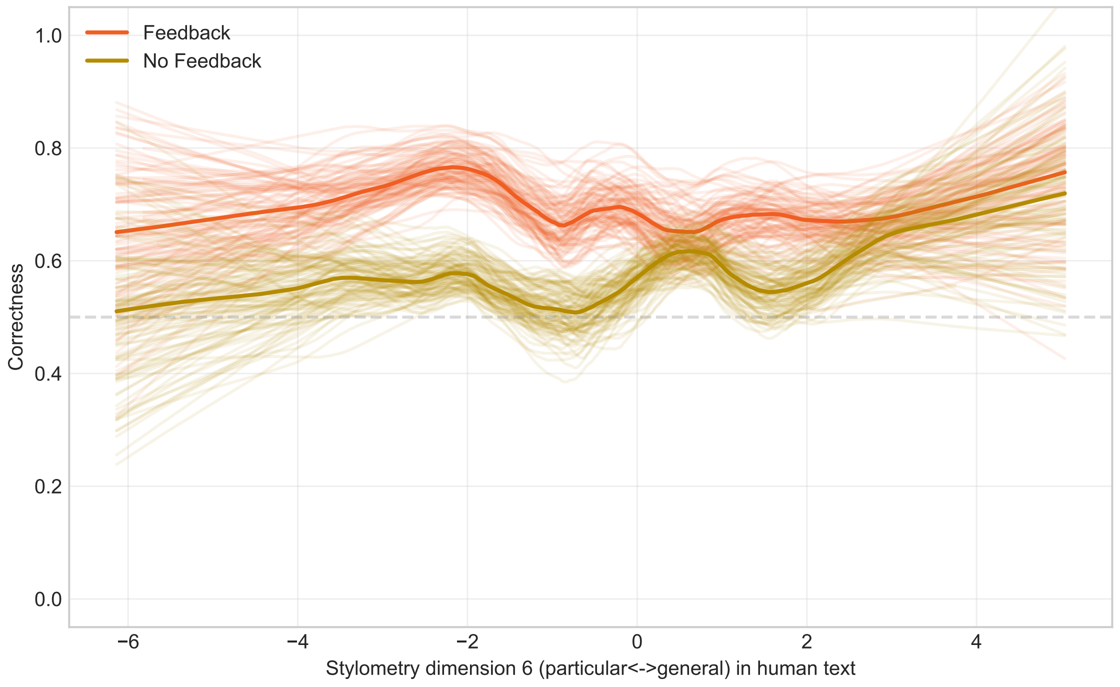

Figure 33: Dependence of Position of human text on 6th stylometric dimension of the answer on Correctness. Bootstrapped 95% confidence intervals.

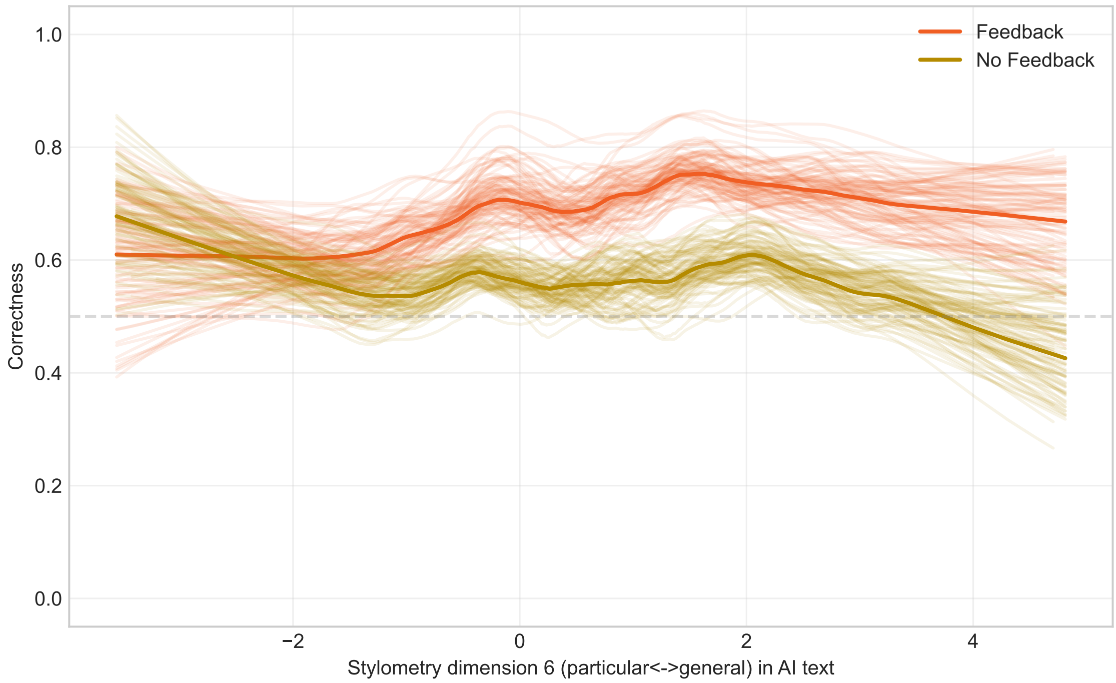

Figure 34: Dependence of Position of AI text on 6th stylometric dimension of the answer on Correctness. Bootstrapped 95% confidence intervals.

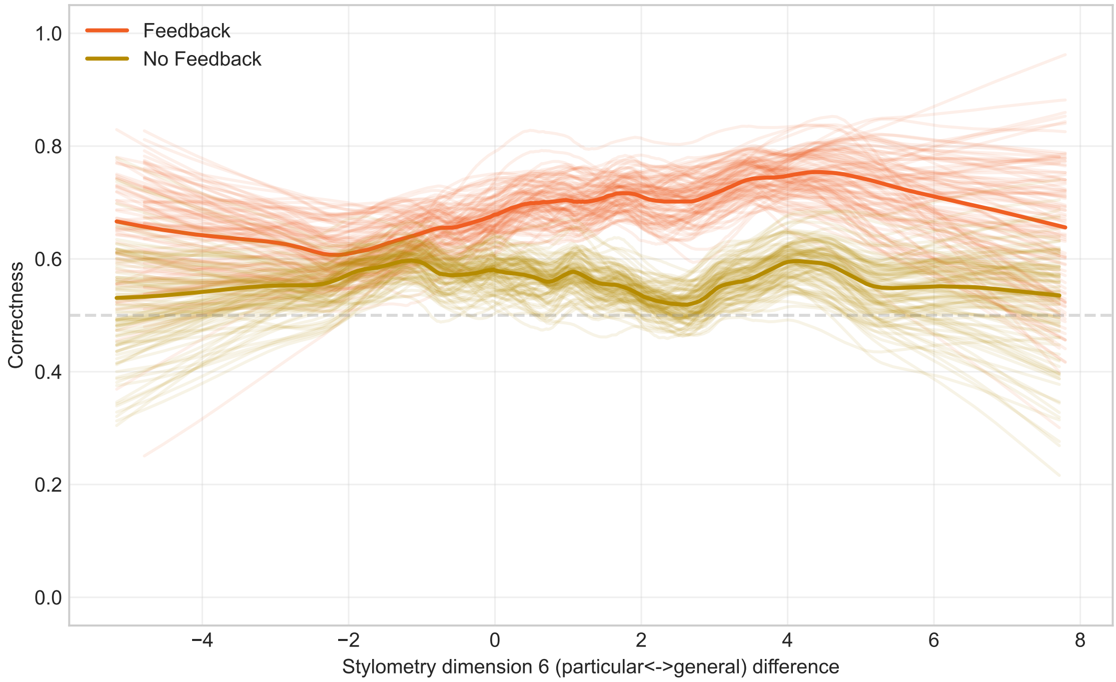

Figure 35: Dependence of Difference between AI and human text on 6th stylometric dimension of the answer on Correctness. Bootstrapped 95% confidence intervals.

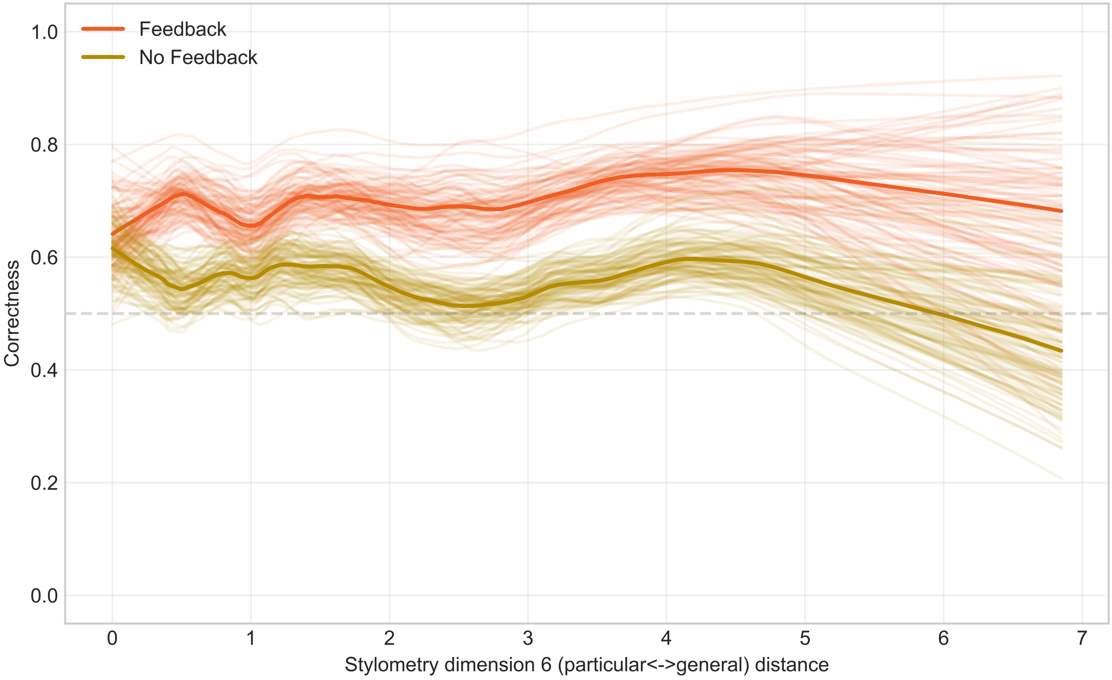

Figure 36: Dependence of Distance between AI and human text on 6th stylometric dimension of the answer on Correctness. Bootstrapped 95% confidence intervals.

9.7.7 Prospective (+) × Retrospective (-) (*GLS7*)

This dimension expresses the tendency of the text to use present and future tense (non-narrative) versus past tense (narrative).

The dependence of correctness on the position of the human-written text along this dimension is shown in Figure 37. The dependence of correctness on the position of the AI-generated text is in Figure 38. The dependence of correctness on the difference between them is in Figure 39, and the dependence on the distance between them (i.e., the absolute value of the difference) is in Figure 40.

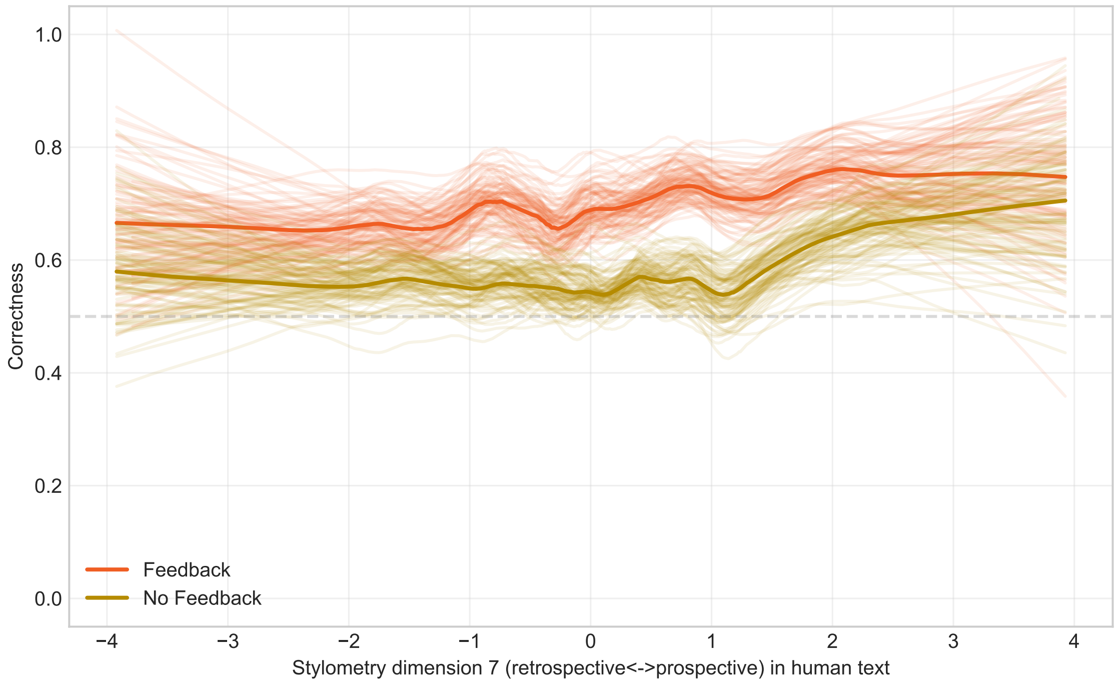

Figure 37: Dependence of Position of human text on 7th stylometric dimension of the answer on Correctness. Bootstrapped 95% confidence intervals.

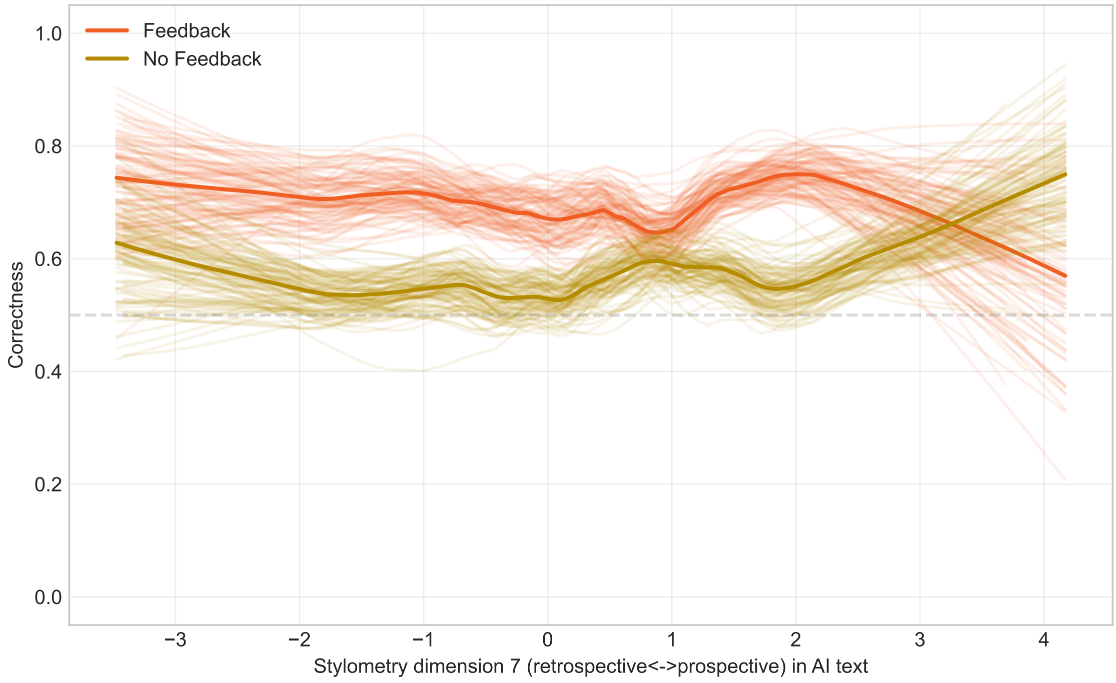

Figure 38: Dependence of Position of AI text on 7th stylometric dimension of the answer on Correctness. Bootstrapped 95% confidence intervals.

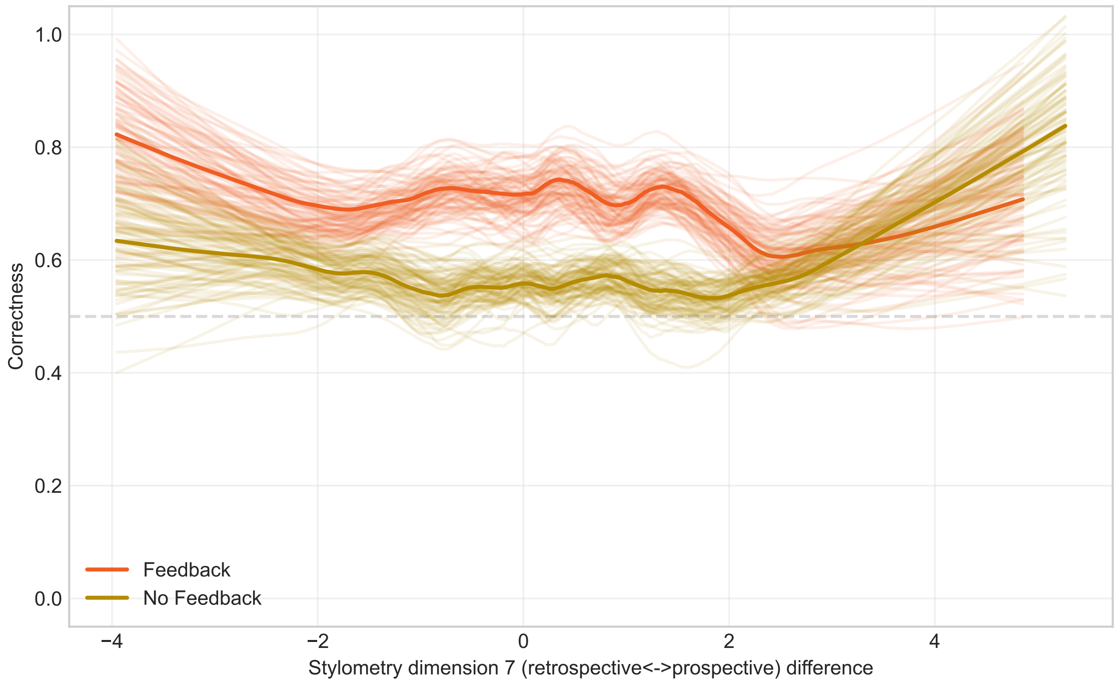

Figure 39: Dependence of Difference between AI and human text on 7th stylometric dimension of the answer on Correctness. Bootstrapped 95% confidence intervals.

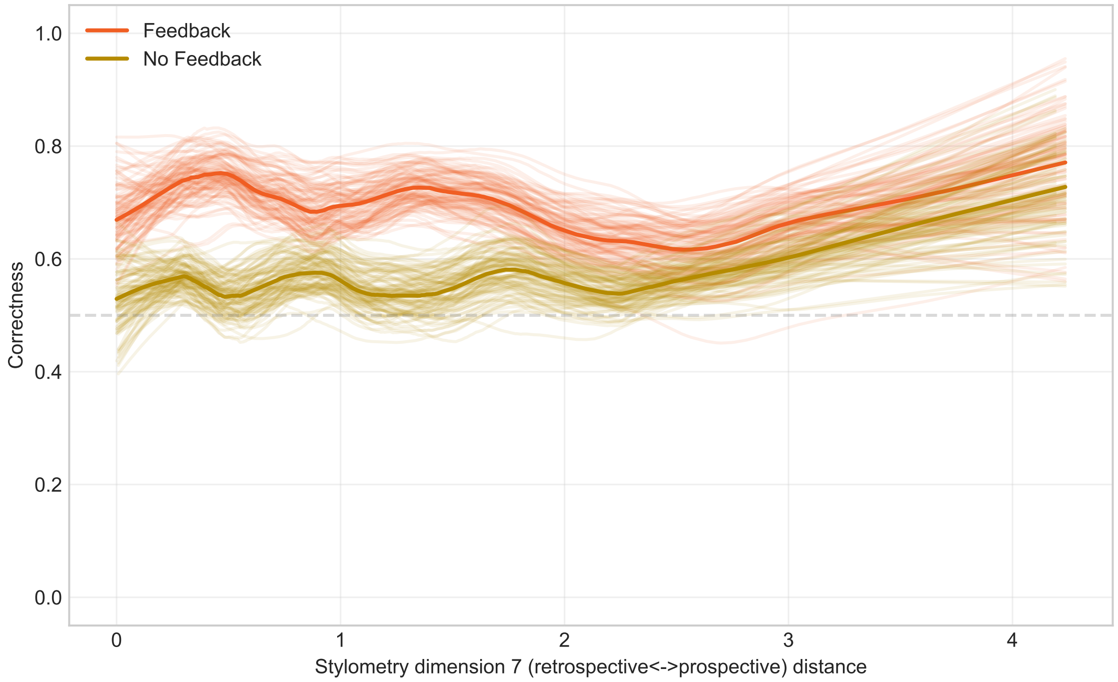

Figure 40: Dependence of Distance between AI and human text on 7th stylometric dimension of the answer on Correctness. Bootstrapped 95% confidence intervals.

9.7.8 Attitudinal (+) × Factual (-) (*GLS8*)

This dimension expresses the degree of explicit epistemic certainty and the amount of hedging—higher versus lower.

The dependence of correctness on the position of the human-written text along this dimension is shown in Figure 41. The dependence of correctness on the position of the AI-generated text is in Figure 42. The dependence of correctness on the difference between them is in Figure 43, and the dependence on the distance between them (i.e., the absolute value of the difference) is in Figure 44.

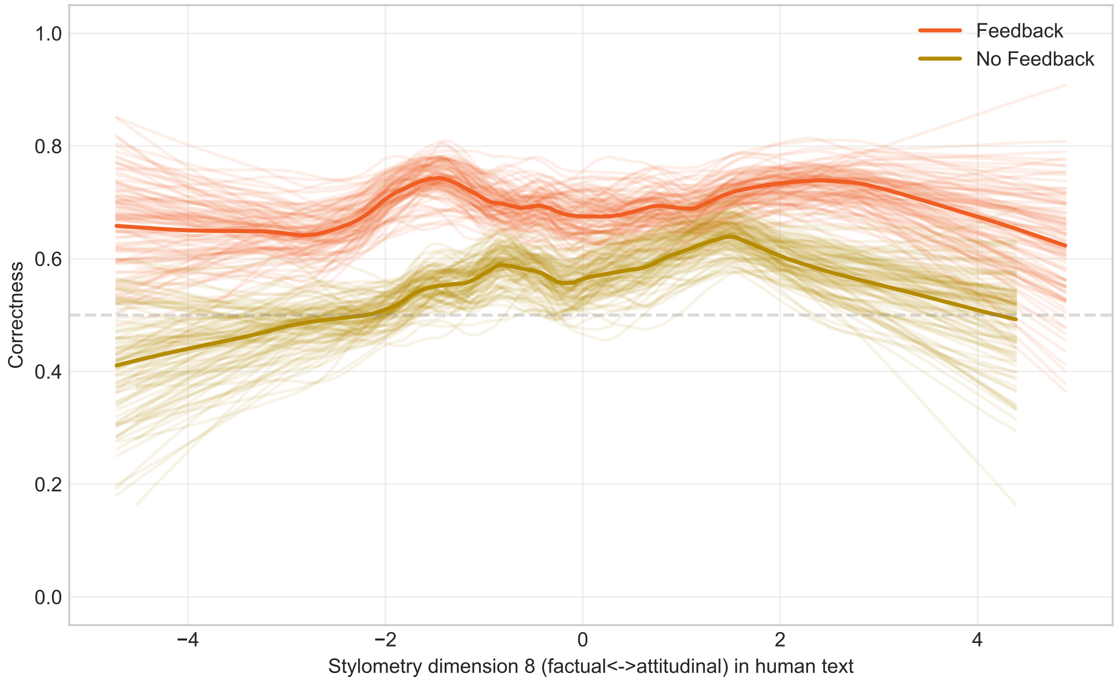

Figure 41: Dependence of Position of human text on 8th stylometric dimension of the answer on Correctness. Bootstrapped 95% confidence intervals.

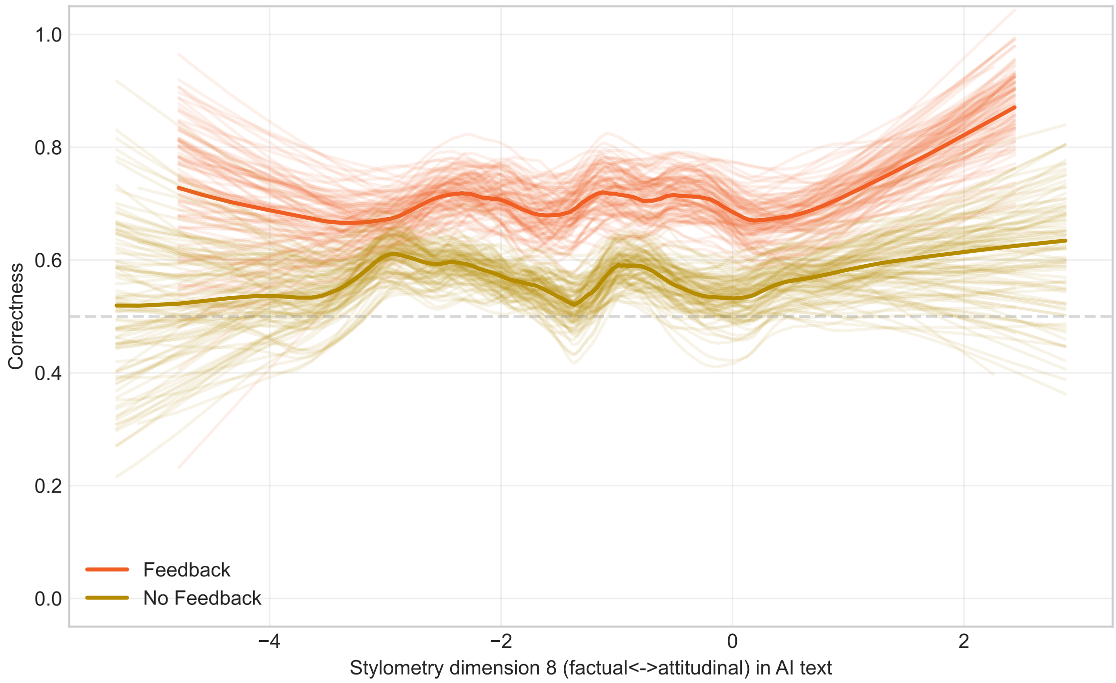

Figure 42: Dependence of Position of AI text on 8th stylometric dimension of the answer on Correctness. Bootstrapped 95% confidence intervals.

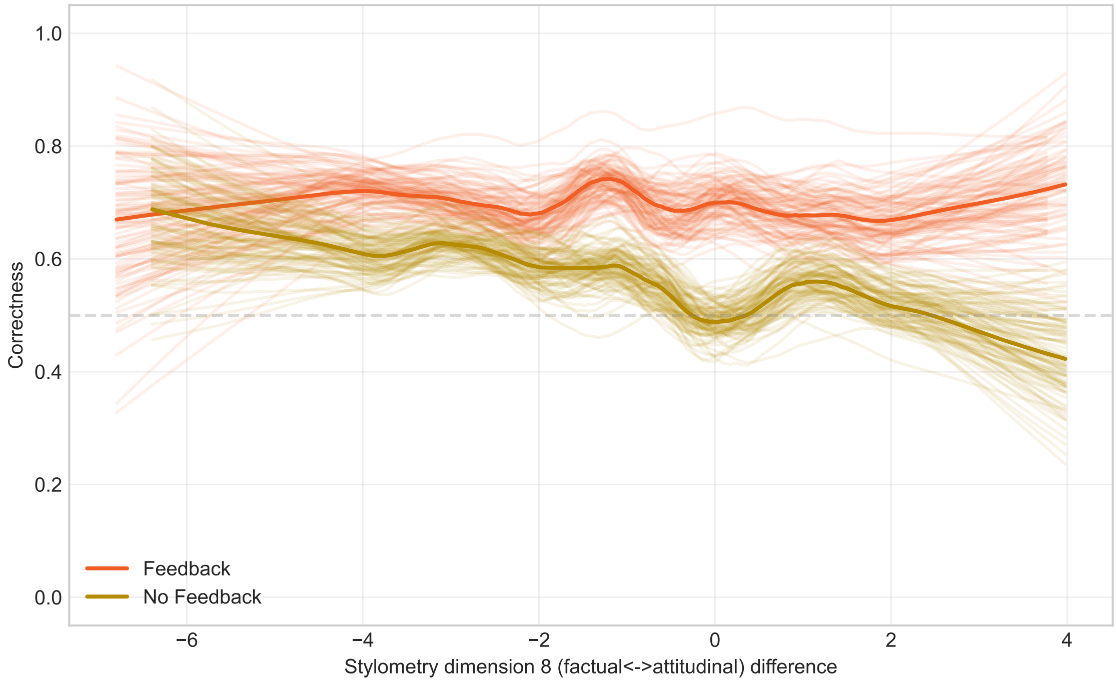

Figure 43: Dependence of Difference between AI and human text on 8th stylometric dimension of the answer on Correctness. Bootstrapped 95% confidence intervals.

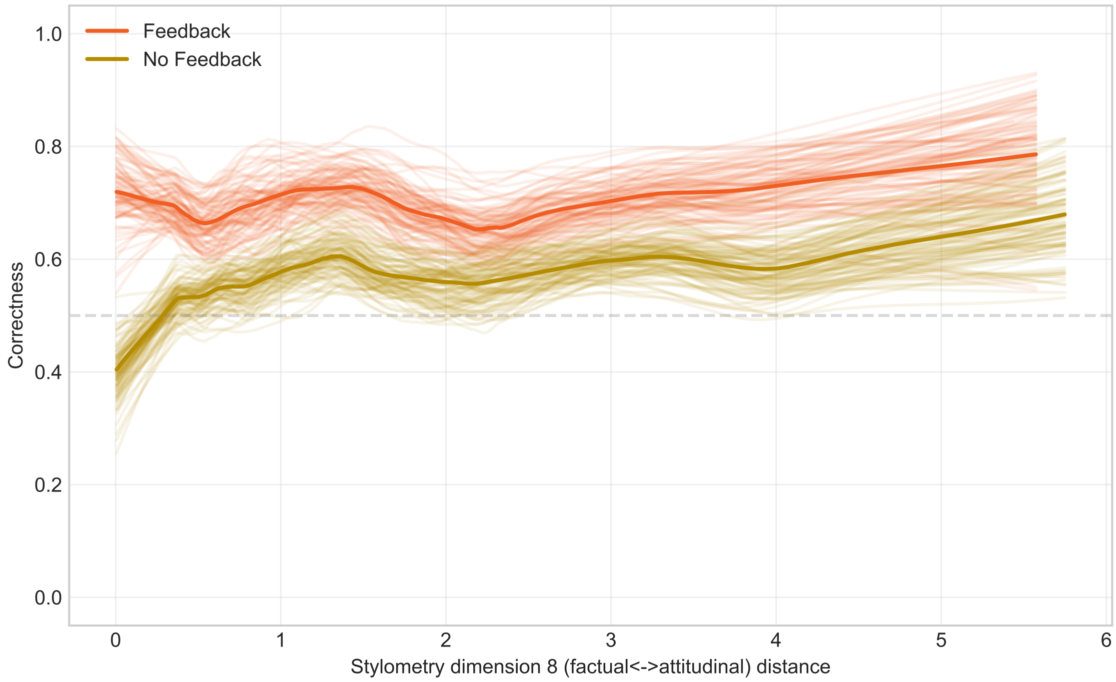

Figure 44: Dependence of Distance between AI and human text on 8th stylometric dimension of the answer on Correctness. Bootstrapped 95% confidence intervals.

9.8 Age

45

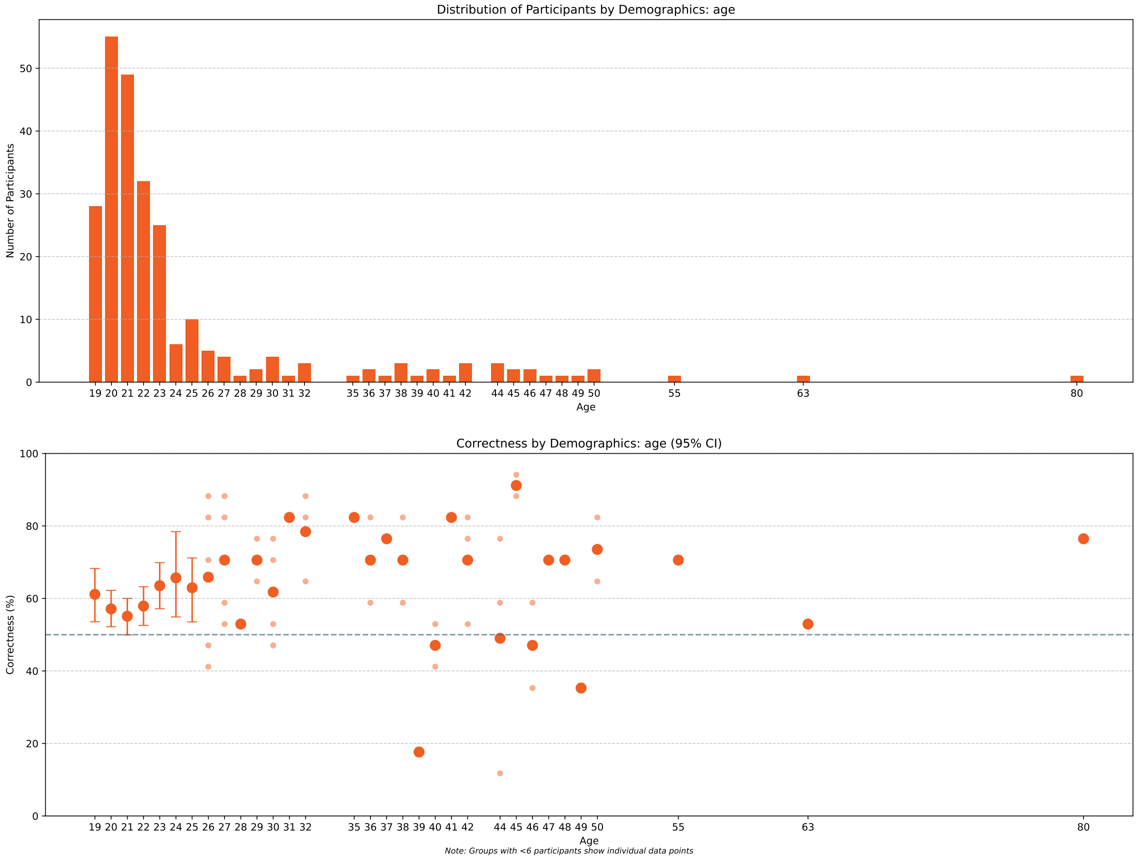

Figure 45: Histogram of Age and dependence of Correctness of the answer on Age. Bootstrapped 95% confidence intervals.

9.9 Gender

46

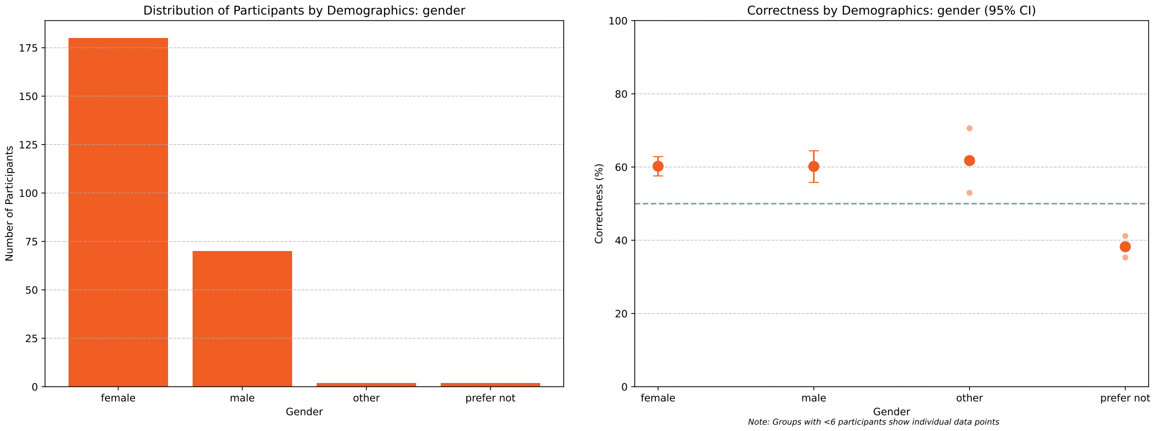

Figure 46: Histogram of Gender and dependence of Correctness of the answer on Gender. Bootstrapped 95% confidence intervals.

9.10 Education(*education_merged*)

47

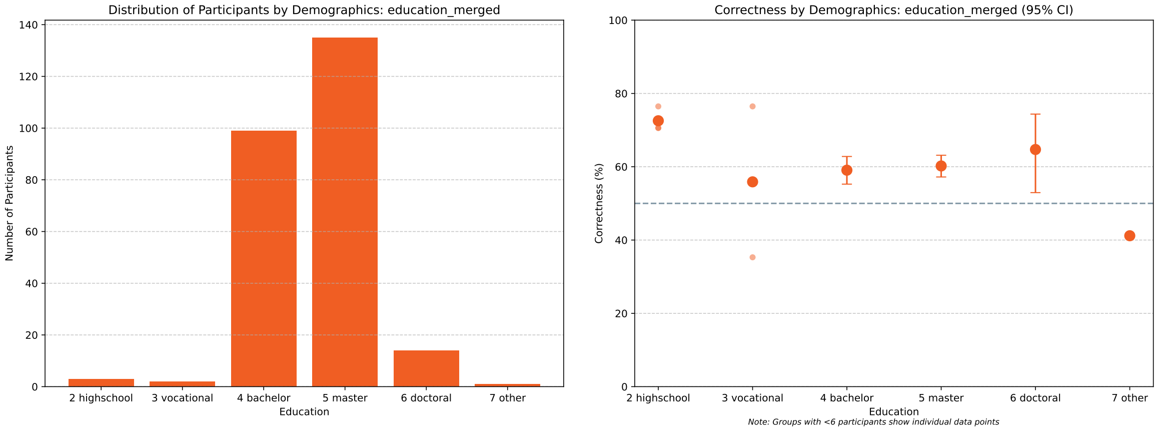

Figure 47: Histogram of Education and dependence of Correctness of the answer on Education. Bootstrapped 95% confidence intervals.

9.11 Field of Study (*field_merged*)

48

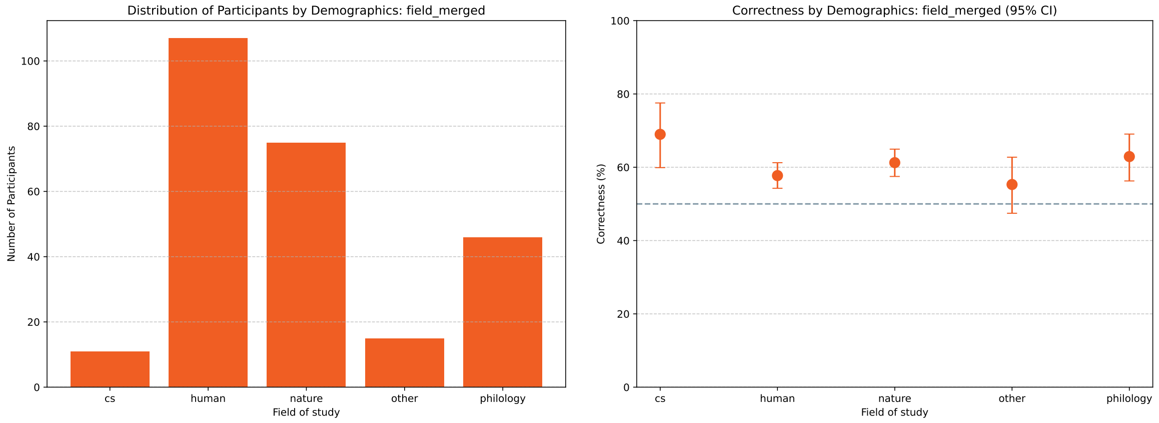

Figure 48: Histogram of Field of study and dependence of Correctness of the answer on Field of study. Bootstrapped 95% confidence intervals.

9.12 Participant Has Reading Disorder (*reading_disorder*)

49

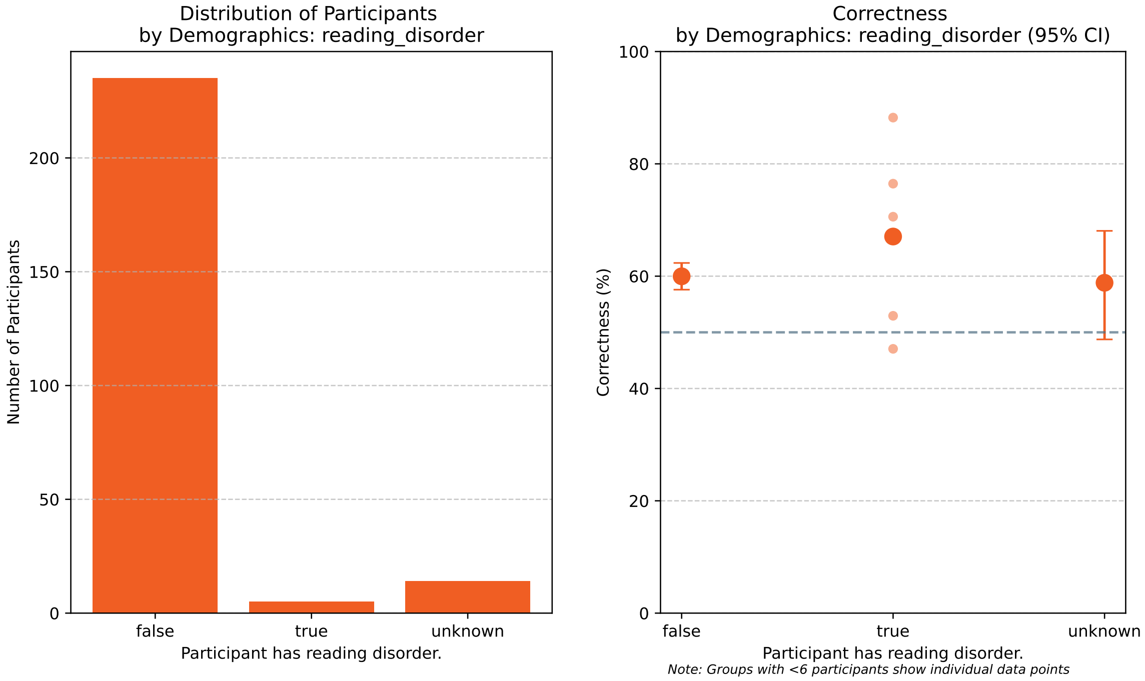

Figure 49: Histogram of Reading disorder and dependence of Correctness of the answer on Reading disorder. Bootstrapped 95% confidence intervals.

9.13 AI Can Improve the Quality of Our Daily Life (*ai_improve*)

50

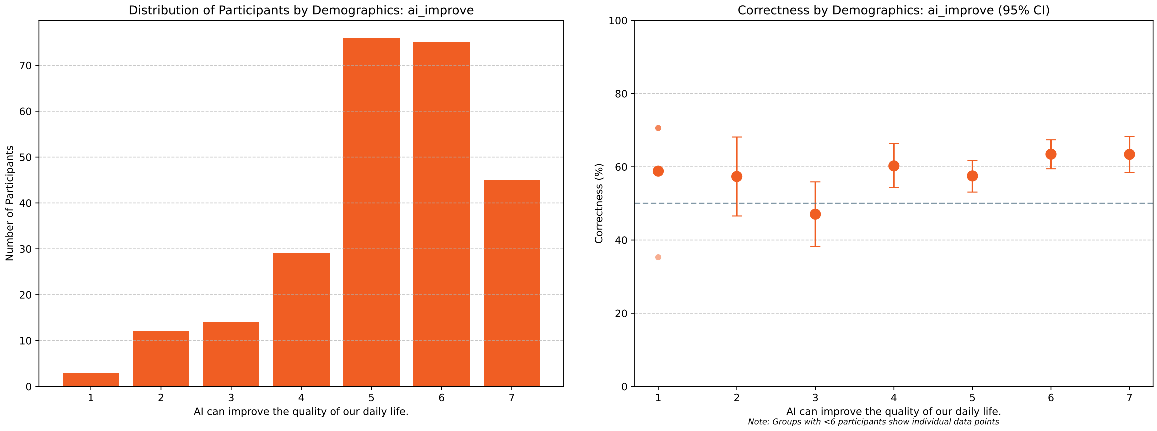

Figure 50: Histogram of *ai_improve* and dependence of Correctness of the answer on *ai_improve*. Bootstrapped 95% confidence intervals.

9.14 AI Poses a Risk to Human Safety (*ai_risk*)

51

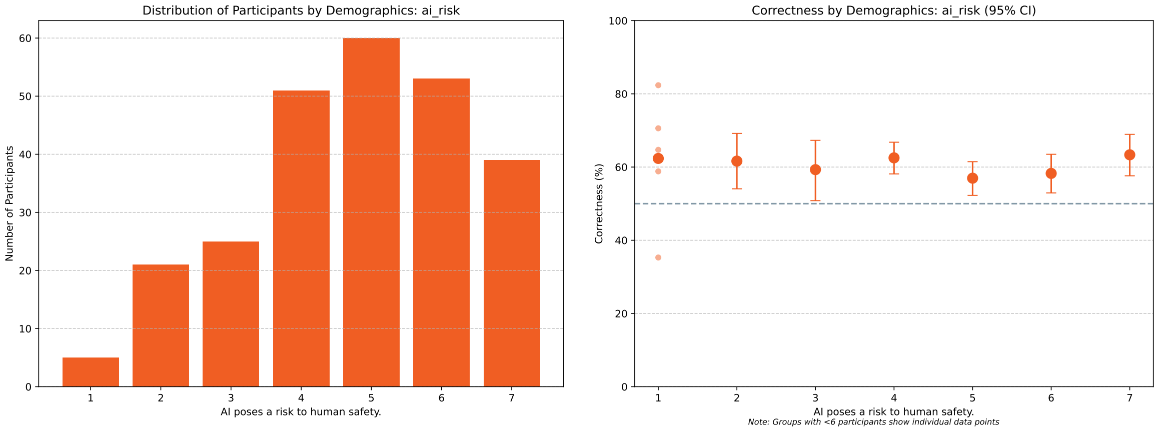

Figure 51: Histogram of *ai_risk* and dependence of Correctness of the answer on *ai_risk*. Bootstrapped 95% confidence intervals.

9.15 Automatically Generated Texts Can Be as Good as Human-Written Texts (*ai_quality*)

52

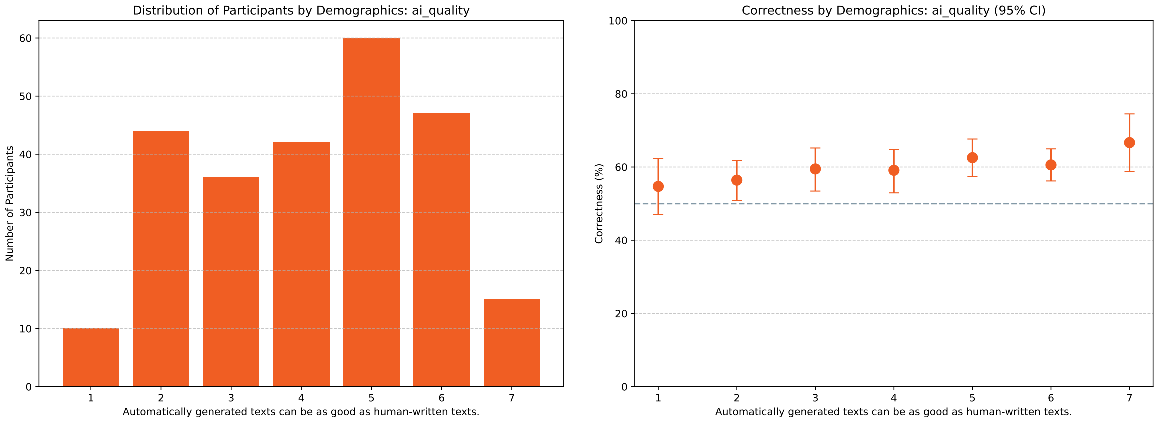

Figure 52: Histogram of *ai_quality* and dependence of Correctness of the answer on *ai_quality*. Bootstrapped 95% confidence intervals.

9.16 Automatically Generated Texts Lack Human Creativity and Personal Style (*ai_lacks_creativity*)

53

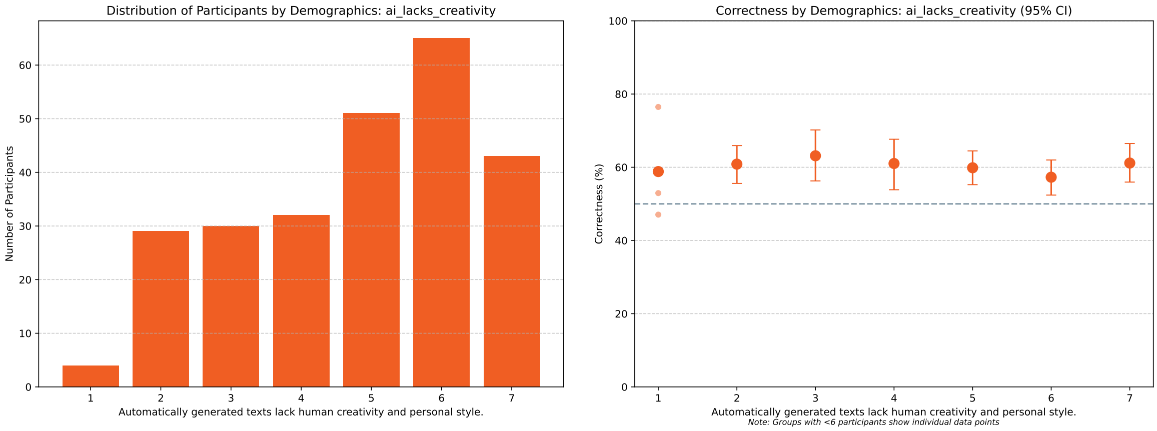

Figure 53: Histogram of *ai_lacks_creativity* and dependence of Correctness of the answer on *ai_lacks_creativity*. Bootstrapped 95% confidence intervals.

9.17 AI Is Not Capable of True Understanding Like Humans (*ai_lacks_understanding*)

54

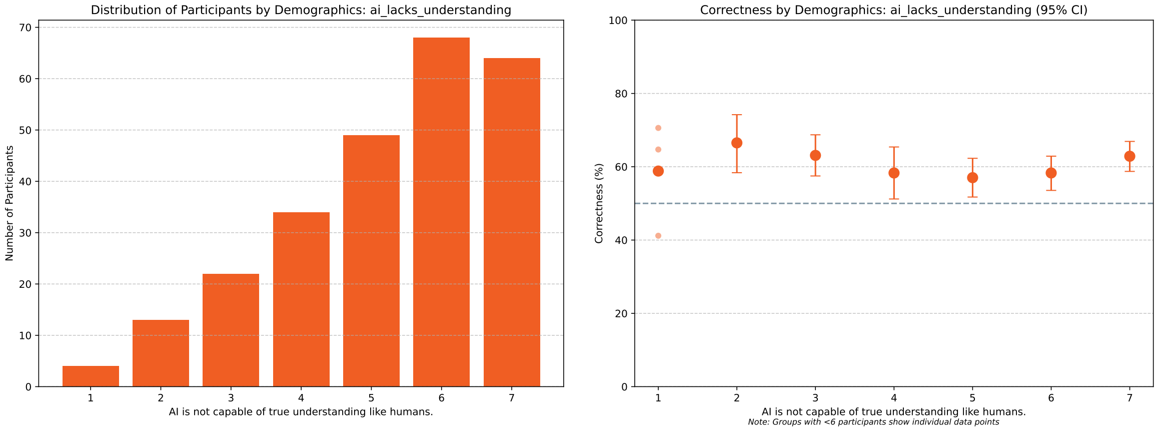

Figure 54: Histogram of *ai_lacks_understanding* and dependence of Correctness of the answer on *ai_lacks_understanding*. Bootstrapped 95% confidence intervals.

9.18 AI Produces Grammatically Correct Texts (*ai_grammar*)

55

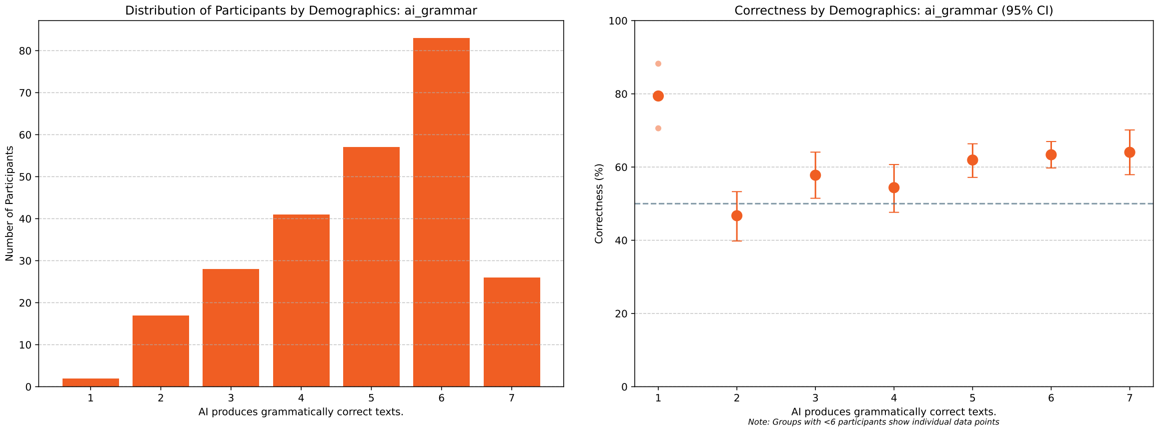

Figure 55: Histogram of *ai_grammar* and dependence of Correctness of the answer on *ai_grammar*. Bootstrapped 95% confidence intervals.

9.19 I Prefer to Avoid Using AI When Writing Important Texts (*ai_avoidance*)

56

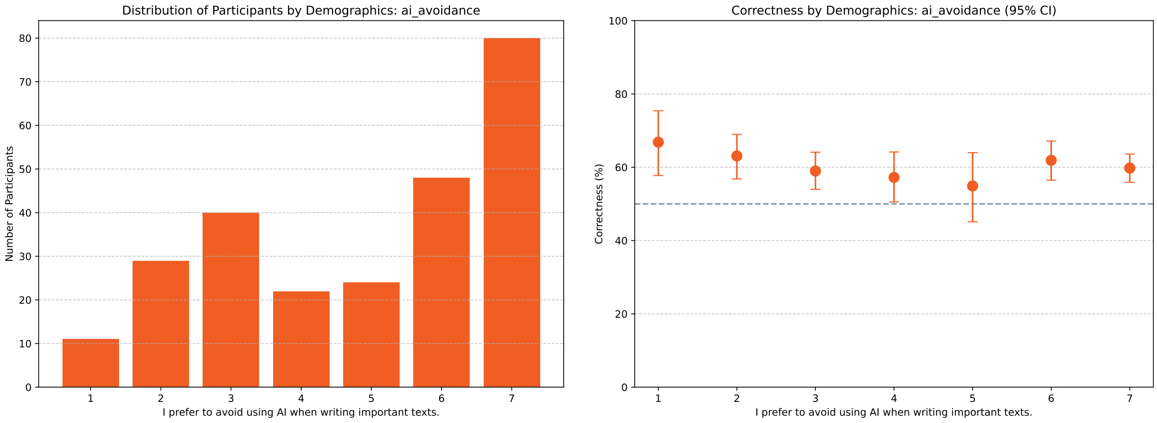

Figure 56: Histogram of *ai_avoidance* and dependence of Correctness of the answer on *ai_avoidance*. Bootstrapped 95% confidence intervals.

9.20 I Am Concerned That People Are Becoming Dumber Because of AI (*ai_dumbing*)

57

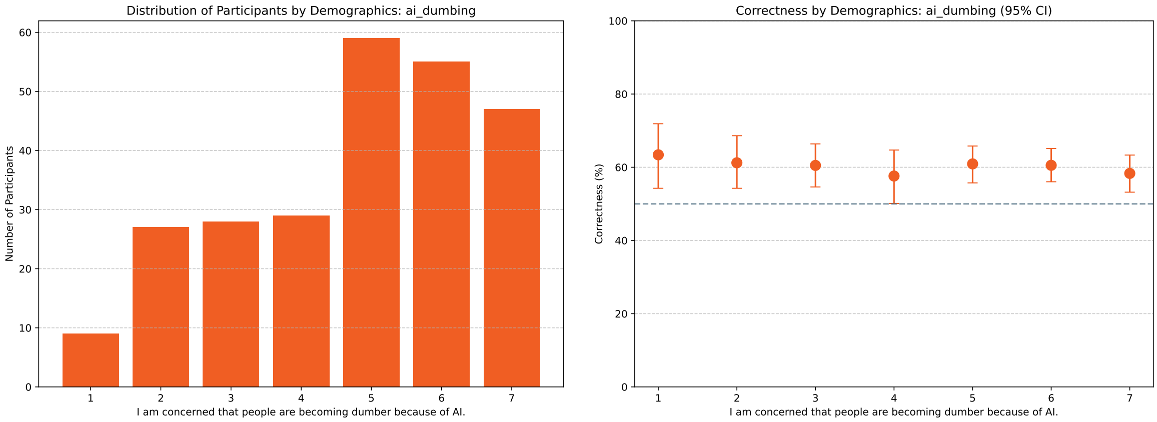

Figure 57: Histogram of *ai_dumbing* and dependence of Correctness of the answer on *ai_dumbing*. Bootstrapped 95% confidence intervals.

9.21 I Am Fascinated by What AI Can Do in Text Generation (*ai_fascination*)

58

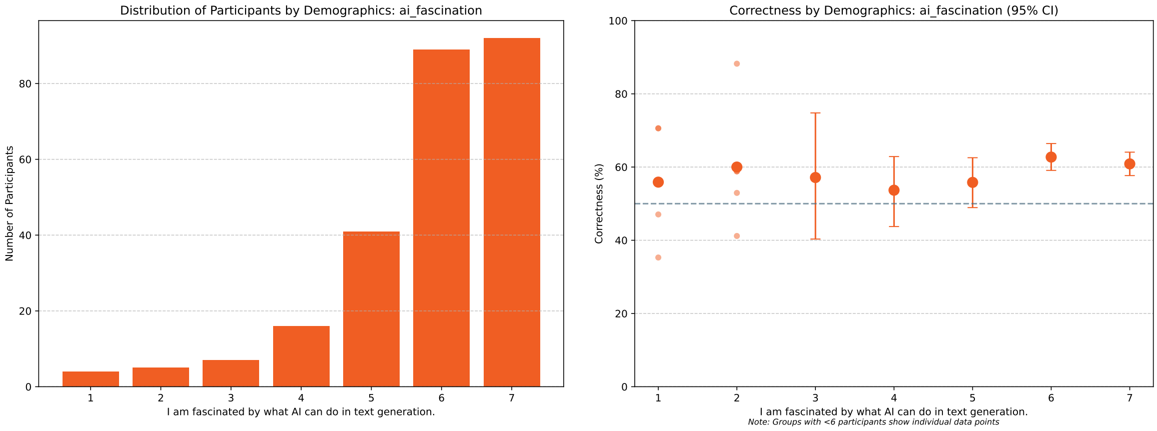

Figure 58: Histogram of *ai_fascination* and dependence of Correctness of the answer on *ai_fascination*. Bootstrapped 95% confidence intervals.

9.22 How Often Do You Use Large Language Models? (*ai_usage*)

59

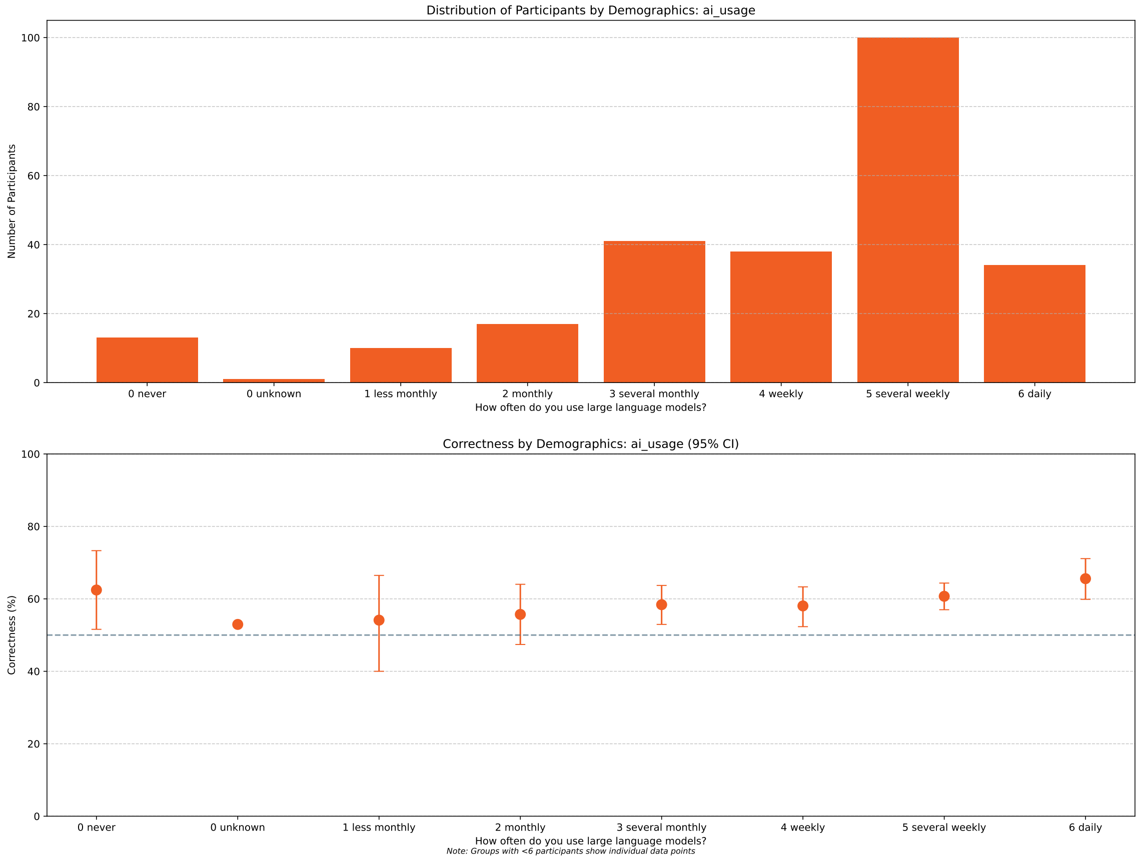

Figure 59: Histogram of AI usage frequency and dependence of Correctness of the answer on AI usage frequency. Bootstrapped 95% confidence intervals.

10 Mixed Effects Logistic Regression

For this analysis, the statistical software Jamovi (under `https://www.jamovi.org/`, last retrieved 29.12.2023) was utilized, using GAMLj package (`https://gamlj.github.io/glmmixed_example1.html`). Its main advantage lies in the self-contained file format, which encapsulates all relevant data and settings (to be found in the `Analysis/mixed models jamovi/jamovi files` directory).

Initially, the data in `results/LABELS_processed.tsv` were imported to Jamovi. The analysis was subsequently launched, focusing on the target variable `Iscorrect` (correctness of the answer on each trial).

Variables:

- *got_feedback*;
- *More readable is human*;
- *Confidence*;
- *Reaction time log*;
- *id* (trial order);
- *GLS Euclidean distance*;
- *GLS[1–4] Human*;
- *GLS[1–4] Difference*;
- *Demographics: age*;
- *Demographics: education_ordinal*;
- *Demographics: ai_usage_ordina*;
- *Demographics: gender_merged*;
- *Demographics: ai_negative_attitude*;
- *Demographics: field_merged*;
- *Demographics: reading_disorder_merged*;

All co-variates were centered.

Analyses were exported to the folder `Scripts/Analysis/mixed models jamovi/outputs`. All reports can be found there, as well as in the appendices of this protocol (specifically D, and E).

11 Generalized Linear Model of Questionnaire Variables

Let us now examine whether the variables described in the questionnaire had an influence. For this purpose, we can use a simple Generalized Linear Model. The results can be seen in `Scripts/Analysis/mixed models jamovi/outputs/Questionaire.omv`.

Details of the output can be found in Appendix F.

12 Declaration on using AI

In the development of certain Python scripts for data analysis, the *GPT-4o* language model by *OpenAI* and *Claude 3.7 Sonnet* by *Anthropic* were consulted. However, all scripts underwent manual review and were, when necessary, corrected or further refined by Jiří Milička. By doing so, Milička assumes full responsibility for the integrity and reliability of these scripts.

In the composition of this article and this accompanying Protocol, both the *GPT-4.5*, *GPT-o3 mini high*, and *GPT-4o* by *OpenAI* and *Claude 3.7 Sonnet* by *Anthropic* were consulted for language styling and review. However, all ideas presented are original to the authors. Similar to the Python scripts, any content generated by the language models was further edited and underwent strict scrutiny.

References

[1] Douglas Biber. *Variation across Speech and Writing.* Cambridge University Press, Cambridge, 1988.

[2] Václav Cvrček, Zuzana Laubeová, David Lukeš, Petra Poukarová, Anna Řehořková, and Adrian Jan Zasina. *Registry v češtině.* NLN, 2020.

[3] David Lukeš. Mdavis. `https://jupyter.korpus.cz/shiny/lukes/mda/`, n.d. Retrieved September 14, 2024.

List of Figures

1 First two dimension . . . 6
2 The distribution of the correctness of the answers. . . . 7
3 The distribution of the readability. . . . 7
4 The distribution of the readability. . . . 8
5 The distribution of the correctness of the answers. . . . 8
6 Dependence of Confidence of the answer on Correctness. Bootstrapped 95% confidence intervals. . . . 9
7 Dependence of Confidence of the answer on Reaction time (log). Bootstrapped 95% confidence intervals. . . . 9
8 Dependence of Trial order of the answer on Confidence. Bootstrapped 95% confidence intervals. . . . 10
9 Dependence of Trial order of the answer on Correctness. Bootstrapped 95% confidence intervals. . . . 11
10 Dependence of Trial order of the answer on Reaction time (log). Bootstrapped 95% confidence intervals. . . . 12
11 Dependence of Reaction time (log) of the answer on Correctness. Bootstrapped 95% confidence intervals. . . . 13
12 Dependence of Euclidean distance of stylometric vectors of the answer on Correctness. Bootstrapped 95% confidence intervals. . . . 14
13 Dependence of Position of human text on 1st stylometric dimension of the answer on Correctness. Bootstrapped 95% confidence intervals. . . . 15
14 Dependence of Position of AI text on 1st stylometric dimension of the answer on Correctness. Bootstrapped 95% confidence intervals. . . . 15
15 Dependence of Difference between AI and human text on 1st stylometric dimension of the answer on Correctness. Bootstrapped 95% confidence intervals. . . . 16
16 Dependence of Distance between AI and human text on 1st stylometric dimension of the answer on Correctness. Bootstrapped 95% confidence intervals. . . . 16
17 Dependence of Position of human text on 2nd stylometric dimension of the answer on Correctness. Bootstrapped 95% confidence intervals. . . . 17
18 Dependence of Position of AI text on 2nd stylometric dimension of the answer on Correctness. Bootstrapped 95% confidence intervals. . . . 18
19 Dependence of Difference between AI and human text on 2nd stylometric dimension of the answer on Correctness. Bootstrapped 95% confidence intervals. . . . 18
20 Dependence of Distance between AI and human text on 2nd stylometric dimension of the answer on Correctness. Bootstrapped 95% confidence intervals. . . . 19
21 Dependence of Position of human text on 3rd stylometric dimension of the answer on Correctness. Bootstrapped 95% confidence intervals. . . . 20
22 Dependence of Position of AI text on 3rd stylometric dimension of the answer on Correctness. Bootstrapped 95% confidence intervals. . . . 20
23 Dependence of Difference between AI and human text on 3rd stylometric dimension of the answer on Correctness. Bootstrapped 95% confidence intervals. . . . 21
24 Dependence of Distance between AI and human text on 3rd stylometric dimension of the answer on Correctness. Bootstrapped 95% confidence intervals. . . . 21
25 Dependence of Position of human text on 4th stylometric dimension of the answer on Correctness. Bootstrapped 95% confidence intervals. . . . 22
26 Dependence of Position of AI text on 4th stylometric dimension of the answer on Correctness. Bootstrapped 95% confidence intervals. . . . 23
27 Dependence of Difference between AI and human text on 4th stylometric dimension of the answer on Correctness. Bootstrapped 95% confidence intervals. . . . 23
28 Dependence of Distance between AI and human text on 4th stylometric dimension of the answer on Correctness. Bootstrapped 95% confidence intervals. . . . 24
29 Dependence of Position of human text on 5th stylometric dimension of the answer on Correctness. Bootstrapped 95% confidence intervals. . . . 25
30 Dependence of Position of AI text on 5th stylometric dimension of the answer on Correctness. Bootstrapped 95% confidence intervals. . . . 25

31 Dependence of Difference between AI and human text on 5th stylometric dimension of the answer on Correctness. Bootstrapped 95% confidence intervals. 26
32 Dependence of Distance between AI and human text on 5th stylometric dimension of the answer on Correctness. Bootstrapped 95% confidence intervals. 26
33 Dependence of Position of human text on 6th stylometric dimension of the answer on Correctness. Bootstrapped 95% confidence intervals. 27
34 Dependence of Position of AI text on 6th stylometric dimension of the answer on Correctness. Bootstrapped 95% confidence intervals. 28
35 Dependence of Difference between AI and human text on 6th stylometric dimension of the answer on Correctness. Bootstrapped 95% confidence intervals. 28
36 Dependence of Distance between AI and human text on 6th stylometric dimension of the answer on Correctness. Bootstrapped 95% confidence intervals. 29
37 Dependence of Position of human text on 7th stylometric dimension of the answer on Correctness. Bootstrapped 95% confidence intervals. 30
38 Dependence of Position of AI text on 7th stylometric dimension of the answer on Correctness. Bootstrapped 95% confidence intervals. 30
39 Dependence of Difference between AI and human text on 7th stylometric dimension of the answer on Correctness. Bootstrapped 95% confidence intervals. 31
40 Dependence of Distance between AI and human text on 7th stylometric dimension of the answer on Correctness. Bootstrapped 95% confidence intervals. 31
41 Dependence of Position of human text on 8th stylometric dimension of the answer on Correctness. Bootstrapped 95% confidence intervals. 32
42 Dependence of Position of AI text on 8th stylometric dimension of the answer on Correctness. Bootstrapped 95% confidence intervals. 33
43 Dependence of Difference between AI and human text on 8th stylometric dimension of the answer on Correctness. Bootstrapped 95% confidence intervals. 33
44 Dependence of Distance between AI and human text on 8th stylometric dimension of the answer on Correctness. Bootstrapped 95% confidence intervals. 34
45 Histogram of Age and dependence of Correctness of the answer on Age. Bootstrapped 95% confidence intervals. 35
46 Histogram of Gender and dependence of Correctness of the answer on Gender. Bootstrapped 95% confidence intervals. 36
47 Histogram of Education and dependence of Correctness of the answer on Education. Bootstrapped 95% confidence intervals. 37
48 Histogram of Field of study and dependence of Correctness of the answer on Field of study. Bootstrapped 95% confidence intervals. 38
49 Histogram of Reading disorder and dependence of Correctness of the answer on Reading disorder. Bootstrapped 95% confidence intervals. 39
50 Histogram of *ai_improve* and dependence of Correctness of the answer on *ai_improve*. Bootstrapped 95% confidence intervals. 40
51 Histogram of *ai_risk* and dependence of Correctness of the answer on *ai_risk*. Bootstrapped 95% confidence intervals. 41
52 Histogram of *ai_quality* and dependence of Correctness of the answer on *ai_quality*. Bootstrapped 95% confidence intervals. 42
53 Histogram of *ai_lacks_creativity* and dependence of Correctness of the answer on *ai_lacks_creativity*. Bootstrapped 95% confidence intervals. 43
54 Histogram of *ai_lacks_understanding* and dependence of Correctness of the answer on *ai_lacks_understanding*. Bootstrapped 95% confidence intervals. 44
55 Histogram of *ai_grammar* and dependence of Correctness of the answer on *ai_grammar*. Bootstrapped 95% confidence intervals. 45
56 Histogram of *ai_avoidance* and dependence of Correctness of the answer on *ai_avoidance*. Bootstrapped 95% confidence intervals. 46
57 Histogram of *ai_dumbing* and dependence of Correctness of the answer on *ai_dumbing*. Bootstrapped 95% confidence intervals. 47
58 Histogram of *ai_fascination* and dependence of Correctness of the answer on *ai_fascination*. Bootstrapped 95% confidence intervals. 48
59 Histogram of AI usage frequency and dependence of Correctness of the answer on AI usage frequency. Bootstrapped 95% confidence intervals. 49

A Ethics Committee Approval

FILOZOFICKÁ FAKULTA
Univerzita Karlova

Komise pro etiku ve výzkumu

V Praze dne 20.prosince 2024
Č. j.: UKFF/2972/2025

POSUDEK PŘEDLOŽENÉHO NÁVRHU PROJEKTU

Komise pro etiku ve výzkumu FF UK posoudila etickou přípustnost předloženého návrhu projektu *Vliv zpětné vazby na schopnost rozpoznat texty tvořené umělou inteligencí* (v rámci projektu GA24-11725S) a to z hlediska jeho celkového zaměření, plánovaných postupů a nástrojů výzkumu, dostatečnosti předpokládané informovanosti účastníků výzkumu a opatření pro ochranu jejich práv a

neshledala žádné rozpory ani nedostatky

vzhledem k požadavkům vyplývajících z právních předpisů České republiky, vnitřních a dalších předpisů univerzity a fakulty a specifických požadavků poskytovatele finančních prostředků a dalších orgánů a institucí, které provedení tohoto posudku nárokují.

Univerzita Karlova * FF UK (5) * Filozofická fakulta

předseda komise
Doc. Mgr. Jiří Lukavský, Ph.D.

člen komise

Komise pro etiku ve výzkumu
Filozofická fakulta Univerzity Karlovy
nám. Jana Palacha 2, 116 38 Praha 1
IČ: 00216208
DIČ: CZ00216208

kev@ff.cuni.cz

http://www.ff.cuni.cz/fakulta/organy-fakulty/komise-ff-uk/komise-pro-etiku-ve-vyzkumu/

B Method proposal for Ethics Committee

Žádost o etické posouzení projektu

Popis projektu

**Název**	Vliv zpětné vazby na schopnost rozpoznat texty tvořené umělou inteligencí
**Financován**	je
**Hlavní řešitel**	Jiří Milička
**Základní součást**	Ústav Českého národního korpusu
**Kontaktní e-mail**	jiri.milicka@ff.cuni.cz
**Telefon**	+420 736 131 551
**Popis** (max. 2000 znaků)	Experiment, který je součástí projektu *Velké jazykové modely prizmatem korpusové lingvistiky* (GA24-11725S), podpořeného Grantovou agenturou České republiky, se zaměří na schopnost mluvčích češtiny a angličtiny rozeznat texty vytvořené umělou inteligencí od textů, jejichž autory jsou lidé. V experimentu budou využity texty z korpusu českých a anglických textů tvořených velkými jazykovými modely, které byly vygenerovány na základě existujících beletristických děl, vědeckých článků a dalších psaných děl s lidskými autory. Experiment bude probíhat online, participantovi se na obrazovce nejprve objeví dva kratší texty, které se oba budou věnovat stejnému tématu, jeden však bude napsaný člověkem a druhý bude sepsaný velkým jazykovým modelem. Participant bude mít po přečtení obou textů za úkol zodpovědět několik otázek. Pod oběma texty se nejprve zobrazí otázka, který ze dvou textů se participantovi lépe četl. Po jejím zodpovězení bude participant odpovídat na to, který z textů je dle něj napsán člověkem, a na Likertově škále následně participant vyznačí, jak jistý si je svou předchozí odpovědí. U některých textů bude participant ještě zodpovídat kontrolní otázku zaměřenou na jejich obsah. Po ohodnocení každé dvojice textů se polovině účastníků zobrazí informace o tom, zda text sepsaný člověkem určili správně, druhé polovině participantů budou informace o správnosti jejich odpovědí zobrazeny až po skončení experimentálního úkolu. V rámci testování budou participanti vyplňovat i vybrané demografické údaje (pohlaví, nejvyšší dosažené vzdělání, zaměření studovaného oboru, frekvence práce s umělou inteligencí a postoje k umělé inteligenci). Odpovědi bude zaznamenávat počítačový program, v němž bude úkol administrován. Experimentu se zúčastní skupina rodilých mluvčích češtiny a rodilých mluvčích angličtiny, popřípadě jiných jazyků, a to především z řad vysokoškolských studentů.

Otázky na etické aspekty projektu

1. Zahrnuje váš výzkum lidské účastníky?

ANO/~~NE~~

Pokud ANO:

a. Účastní se výzkumu na základě vlastního rozhodnutí?

ANO/~~NE~~

Popište, jak jsou účastníci osloveni, jaká jsou kritéria pro jejich zapojení do výzkumu a proces získání informovaného souhlasu.

Skupina česky hovořících participantů bude sestávat především z frekventantů kurzu *Účast na lingvistických a psychologických experimentech v laboratoři LABELS*, participanti budou osloveni prostřednictvím doc. Jana Chromého, Ph.D., který daný kurz vede. Participanti musí být rodilí mluvčí češtiny. Anglicky hovořící participanti budou osloveni především skrze kontaktů na spřátelených anglosaských univerzitách. Experiment bude distribuován také online, pomocí sociálních sítí a přátel a známých organizátorů.

Formulář informovaného souhlasu bude v PDF formě připojen do samotného online experimentu, participanti si jej po kliknutí na odkaz budou moci zobrazit a přečíst. Zaškrtnutím tlačítka poté budou indikovat, že informovaný souhlas přečetli a souhlasí s ním. Pro skupinu anglicky hovořících participantů bude formulář informovaného souhlasu přeložen do angličtiny.

b. Jedná se o účastníky, kteří nemohou poskytnout informovaný souhlas (včetně dětí a mladistvých)?

~~ANO~~/NE

Popište, jak získáte souhlas s účastí ve výzkumu od zákonných zástupců účastníků a souhlas dětí a mladistvých. Jak zajistíte, aby na účastníky nebyl vytvořen nátlak na jejich zapojení do výzkumu? Jaká opatření plánujete na ochranu dětí v průběhu výzkumu? Zdůvodněte zapojení dětí a mladistvých do výzkumu.

c. Patří účastníci mezi zvláště zranitelné osoby nebo skupiny?

~~ANO~~/NE

O jaký druh znevýhodnění/ohrožení se jedná?

Popište, jak jsou účastníci osloveni, jaká jsou kritéria pro jejich zapojení do výzkumu a proces získání informovaného souhlasu.

d. Jsou účastníci pacienty?

~~ANO~~/NE

Jaká je povaha jejich nemoci nebo zdravotního omezení? Popište, jak jsou účastníci osloveni, jaká jsou kritéria pro jejich zapojení do výzkumu a proces získání informovaného souhlasu.

2. Pracuje váš výzkum s osobními daty účastníků? Shromažďuje a zpracovává tato data?

ANO/~~NE~~

Pokud ANO:

a. Jaký je proces sběru dat, jejich uložení a ochrany, předávání a následného vymazání dat? Jak budou data uložena (LAN, cloud, atd.)? Jaká bude struktura získávaných dat a jejich ochrana (kódování, anonymizace, atd.)? Jak budou data zpracována a příp. dále využívána?

V rámci výzkumu budou sbírána následující demografická data: pohlaví, věk, nejvyšší dosažený stupeň vzdělání, zaměření (vy)studovaného oboru, přítomnost potíží se čtením, frekvence práce s umělou inteligencí a postoje k umělé inteligenci. Tato data budou zaznamenána počítačovým programem, v němž bude experiment administrován. Sběr bude provedený tak, že nebude možné spojit identitu konkrétního participanta s odpověďmi. Počítač bude rovněž zaznamenávat experimentální data – ukládány budou odpovědi na jednotlivé doprovodné otázky, a to ve formě volby jednoho z textů a bodu na Likertově škále, zaznamenána bude rovněž správnost odpovědi na otázku zjišťující, který text byl vytvořen člověkem. Takto získaná demografická a experimentální data budou převedena do tabulkového formátu a anonymizována (jednotliví participanti budou identifikováni pomocí alfanumerických kódů) a následně s pomocí statistických programů vyhodnocena. Anonymizované datové soubory pro vyhodnocení experimentů budou uchovány jednak na osobním počítači pod heslem, jednak na cloudovém úložišti iniciativy Open Science Framework (osf.io).

b. Jaká bezpečnostní opatření plánujete k ochraně dat?

Veškerá data budou uložena na počítači chráněném heslem.

c. Obsahují získávaná data citlivé osobní údaje (např. zdravotní stav, sexuální orientaci, etnickou příslušnost, politické názory, náboženské přesvědčení apod.)?

ANO/~~NE~~

Jaká konkrétně?

Zjišťovány budou informace o případných poruchách čtení, o práci s umělou inteligencí a postojích k umělé inteligenci.

d. Zahrnuje výzkum sledování nebo pozorování účastníků? (např. sledování pohybu osob, údaje o poloze, IP adresy apod.)?

~~ANO~~/NE

Popište, jak budou účastníci sledováni nebo pozorováni.

e. Bude váš výzkum zpracovávat již dříve získané osobní údaje?

~~ANO~~/NE

Jaké databáze nebo zdroje dat budete využívat? Jaký bude postup zpracování dat? Jak zabezpečíte data před zneužitím? Potvrďte, že data jsou veřejně dostupná a je možné je využít k dalšímu zpracování, nebo že máte souhlas s jejich využitím. Jak byl souhlas získán? Přiložte potvrzení od vlastníka dat se souhlasem k jejich využití.

3. Budou získaná data poskytnuta dalším subjektům?

ANO/~~NE~~

Pokud ANO:

a. Kterým dalším subjektům v ČR budou data poskytnuta?

Popište důvody sdílení dat s dalším subjektem. Popište proces předávání dat s ohledem na jejich ochranu. Potvrďte, že máte souhlas se sdílením dat s dalším subjektem. Jak byl souhlas získán?

b. Kterým dalším subjektům v zahraničí budou data poskytnuta?

Popište důvody sdílení dat s dalším subjektem. Popište proces předávání dat s ohledem na jejich ochranu. Potvrďte, že máte souhlas se sdílením dat s dalším subjektem. Jak byl souhlas získán?

a. + b. Anonymizovaná experimentální data, výsledky statistických analýz a zdrojový kód daných analýz budou poskytnuty volně k dispozici ve webovém rozhraní iniciativy Open Science Framework (osf.io). Toto rozhodnutí je motivováno dobrou vědeckou praxí a přihlášením se k programu otevřené vědy. Zpřístupnění dat umožní replikovatelnost výzkumu a možnost reanalýzy dat s využitím jiných technik či pro jiné výzkumné otázky. Vzhledem k tomu, že se jedná o open access iniciativu, není možné předem říci, s kým budou data sdílena. Sdíleny budou ovšem pouze anonymizované výsledky experimentů. Informace o zpřístupnění anonymizovaných výsledků je součástí formuláře informovaného souhlasu.

4. Další otázky k doplnění.

C Informed Consent

Informovaný souhlas s účastí ve výzkumu a se zpracováním osobních údajů

Informace o výzkumu:

Tento experiment je součástí projektu Velké jazykové modely prizmatem korpusové lingvistiky, podpořeného Grantovou agenturou České republiky, a zaměřuje se na schopnost rodilých mluvčí češtiny a angličtiny rozpoznat, zda byl text sepsán člověkem, nebo velkým jazykovým modelem. Výzkum provádí tým lingvistů z Filozofické fakulty Univerzity Karlovy, vedoucím týmu je PhDr. Jiří Milička, Ph.D.

Experiment bude probíhat online, vaším úkolem bude přečíst si několik dvojic textů a následně zodpovědět otázky, které se daných dvojic textů budou týkat, před zahájením úkolu ještě bude potřeba vyplnit vybrané demografické údaje (pohlaví, věk, nejvyšší dosažené vzdělání, zaměření studovaného oboru, případné poruchy čtení či jazyka, frekvence práce s umělou inteligencí a postoje k umělé inteligenci), veškeré údaje však budou anonymizovány. Celý experiment by vám měl zabrat cca 20 až 25 minut.

Prohlášení

Potvrzuji, že

a) jsem se seznámil/a s informacemi o cílech a průběhu výše popsaného výzkumu (dále též jen „výzkum");

b) dobrovolně souhlasím s účastí své osoby v tomto výzkumu;

c) rozumím tomu, že se mohu kdykoli rozhodnout ve své účasti na výzkumu nepokračovat;

d) jsem srozuměn/a s tím, že jakékoliv užití a zveřejnění dat a výstupů vzešlých z výzkumu nezakládá můj nárok na jakoukoliv odměnu či náhradu, tzn. že veškerá oprávnění k užití a zveřejnění dat a výstupů vzešlých z výzkumu poskytuji bezúplatně.

Zároveň prohlašuji, že

a) souhlasím se zveřejněním anonymizovaných dat a výstupů vzešlých z výzkumu a s jejich dalším využitím;

b) souhlasím se zpracováním a uchováním osobních a citlivých údajů v rozsahu v tomto informovaném souhlasu uvedených ze strany Univerzity Karlovy, Filozofické fakulty, IČ: 00216208, se sídlem: nám. Jana Palacha 2, 116 38 Praha 1, a to pro účely zpracování dat vzešlých z výzkumu, pro účely případného kontaktování z důvodu zpracování dat vzešlých z výzkumu či z důvodu nabídky účasti na obdobných akcích a pro účely evidence a archivace; a s tím, že tyto osobní údaje mohou být poskytnuty subjektům oprávněným k výkonu kontroly projektu, v jehož rámci výzkum realizován;

c) jsem seznámen/a se svými právy týkajícími se přístupu k informacím a jejich ochraně podle § 12 a § 21 zákona č. 101/2000 Sb., o ochraně osobních údajů a o změně některých zákonů, ve znění pozdějších předpisů, tedy že mohu požádat Univerzitu Karlovu v Praze o informaci o zpracování mých osobních a citlivých údajů a jsem oprávněn/a ji dostat a že mohu požádat Univerzitu Karlovu v Praze o opravu nepřesných osobních údajů, doplnění osobních údajů, jejich blokaci a likvidaci.

Výše uvedená svolení a souhlasy poskytuji dobrovolně na dobu neurčitou až do odvolání a zavazuji se je neodvolat bez závažného důvodu spočívajícího v podstatné změně okolností.

Vše výše uvedené se řídí zákony České republiky, s výjimkou tzv. kolizních norem, a bude v souladu s nimi vykládáno, přičemž případné spory budou řešeny příslušnými soudy v České republice.

D Generalized Mixed Model Report (Correctness, participants that got feedback)

Generalized Mixed Model

Model Info

Info	Value	Comment
Model Type	Logistic	Model for binary y
Call	glm	Is correct ~ 1 + More readable is human + Confidence + Demographics: age + Demographics: education_ordinal + Demographics: ai_usage_ordinal + Demographics: gender merged + Demographics: field_merged + Reaction time log + GLS Euclidean distance + GLS1 difference + GLS2 difference + GLS3 difference + GLS4 difference + id + Demographics: reading_disorder_merged + GLS8 difference + GLS7 difference + GLS6 difference + GLS5 difference + Demographics: ai_improve + Demographics: ai_risk + Demographics: ai_quality + Demographics: ai_lacks_creativity + Demographics: ai_lacks_understanding + Demographics: ai_grammar + Demographics: ai_avoidance + Demographics: ai_dumbing + Demographics: ai_fascination + (0 + More readable is human + Confidence + Reaction time log + GLS Euclidean distance + GLS1 difference + GLS2 difference + GLS3 difference + GLS4 difference + id + GLS8 difference + GLS7 difference + GLS6 difference + GLS5 difference | Participant ID)
Link function	Logit	Log of the odd of y=1 over y=0
Direction	P(y=1)/P(y=0)	P(Is correct = 1) / P(Is correct = 0)
Distribution	Binomial	Dichotomous event distribution of y
LogLikel.	-1173.143	Unconditional Log-Likelihood
-2*LogLikel.	2346.287	Unconditional absolute deviance
Deviance	1705.411	Conditional relative deviance
R-squared	NaN	Marginal
R-squared	NaN	Conditional
AIC	2620.290	Less is better
BIC	3392.588	Less is better
Residual DF	1937.000	
Chi-squared/DF	0.675	Overdispersion indicator
Converged	yes	
Optimizer	bobyqa	

Note. R-squared cannot be computed.

Note. boundary (singular) fit: see ?isSingular

Model Results

Fixed Effect Omnibus tests

	X^2	df	p
More readable is human	19.46880	1.00	< .001
Confidence	2.95358	1.00	0.086
Demographics: age	0.13244	1.00	0.716
Demographics: education_ordinal	0.29586	1.00	0.586
Demographics: ai_usage_ordinal	0.69590	1.00	0.404
Demographics: gender merged	0.15981	1.00	0.689
Demographics: field_merged	10.29144	4.00	0.036
Reaction time log	0.02321	1.00	0.879
GLS Euclidean distance	0.00343	1.00	0.953
GLS1 difference	0.59204	1.00	0.442
GLS2 difference	3.41109	1.00	0.065
GLS3 difference	2.16118	1.00	0.142
GLS4 difference	0.67797	1.00	0.410
id	16.08902	1.00	< .001
Demographics: reading_disorder_merged	1.71524	1.00	0.190
GLS8 difference	0.17682	1.00	0.674
GLS7 difference	0.79916	1.00	0.371
GLS6 difference	4.06814	1.00	0.044
GLS5 difference	0.22886	1.00	0.632
Demographics: ai_improve	1.57010	1.00	0.210
Demographics: ai_risk	0.03050	1.00	0.861
Demographics: ai_quality	2.11651	1.00	0.146
Demographics: ai_lacks_creativity	0.45344	1.00	0.501
Demographics: ai_lacks_understanding	1.15976	1.00	0.282
Demographics: ai_grammar	0.06892	1.00	0.793
Demographics: ai_avoidance	5.27193	1.00	0.022
Demographics: ai_dumbing	0.04171	1.00	0.838
Demographics: ai_fascination	0.03826	1.00	0.845

Fixed Effects Parameter Estimates

				95% Confidence Interval			95% Exp(B) Confidence Interval			
**Names**	**Effect**	**Estimate**	**SE**	**Lower**	**Upper**	**exp(B)**	**Lower**	**Upper**	**z**	**p**
(Intercept)	(Intercept)	1.75347	0.3499	1.06769	2.4393	5.775	2.9087	11.464	5.0114	< .001
More readable is human1	1 - 0	1.22118	0.2768	0.67873	1.7636	3.391	1.9714	5.834	4.4123	< .001
Confidence	Confidence	0.08892	0.0517	-0.01249	0.1903	1.093	0.9876	1.210	1.7186	0.086
Demographics: age	Demographics: age	0.00393	0.0108	-0.01723	0.0251	1.004	0.9829	1.025	0.3639	0.716
Demographics: education_ordinal	Demographics: education_ordinal	-0.07256	0.1334	-0.33404	0.1889	0.930	0.7160	1.208	-0.5439	0.586
Demographics: ai_usage_ordinal	Demographics: ai_usage_ordinal	0.05217	0.0625	-0.07040	0.1747	1.054	0.9320	1.191	0.8342	0.404
Demographics: gender merged1	male - female	-0.08266	0.2068	-0.48794	0.3226	0.921	0.6139	1.381	-0.3998	0.689
Demographics: field_merged1	human - cs	-1.70755	0.8003	-3.27602	-0.1391	0.181	0.0378	0.870	-2.1338	0.033
Demographics: field_merged2	nature - cs	-1.68028	0.8012	-3.25057	-0.1100	0.186	0.0388	0.896	-2.0972	0.036
Demographics: field_merged3	other - cs	-1.27117	0.8850	-3.00583	0.4635	0.281	0.0495	1.590	-1.4363	0.151
Demographics: field_merged4	philology - cs	-1.17388	0.7908	-2.72376	0.3760	0.309	0.0656	1.456	-1.4845	0.138
Reaction time log	Reaction time log	0.01424	0.0935	-0.16893	0.1974	1.014	0.8446	1.218	0.1523	0.879
GLS Euclidean distance	GLS Euclidean distance	-0.00341	0.0581	-0.11732	0.1105	0.997	0.8893	1.117	-0.0586	0.953
GLS1 difference	GLS1 difference	-0.08284	0.1077	-0.29387	0.1282	0.920	0.7454	1.137	-0.7694	0.442
GLS2 difference	GLS2 difference	-0.28056	0.1519	-0.57830	0.0172	0.755	0.5608	1.017	-1.8469	0.065
GLS3 difference	GLS3 difference	0.06608	0.0449	-0.02202	0.1542	1.068	0.9782	1.167	1.4701	0.142
GLS4 difference	GLS4 difference	0.04319	0.0525	-0.05962	0.1460	1.044	0.9421	1.157	0.8234	0.410
id	id	0.05304	0.0132	0.02712	0.0790	1.054	1.0275	1.082	4.0111	< .001
Demographics: reading_disorder_merged1	true - false	0.72202	0.5513	-0.35851	1.8025	2.059	0.6987	6.065	1.3097	0.190
GLS8 difference	GLS8 difference	0.01752	0.0417	-0.06414	0.0992	1.018	0.9379	1.104	0.4205	0.674
GLS7 difference	GLS7 difference	-0.04552	0.0509	-0.14532	0.0543	0.956	0.8647	1.056	-0.8940	0.371
GLS6 difference	GLS6 difference	0.07922	0.0393	0.00224	0.1562	1.082	1.0022	1.169	2.0170	0.044
GLS5 difference	GLS5 difference	-0.03025	0.0632	-0.15419	0.0937	0.970	0.8571	1.098	-0.4784	0.632
Demographics: ai_improve	Demographics: ai_improve	0.09082	0.0725	-0.05124	0.2329	1.095	0.9501	1.262	1.2530	0.210
Demographics: ai_risk	Demographics: ai_risk	0.01045	0.0598	-0.10683	0.1277	1.011	0.8987	1.136	0.1746	0.861
Demographics: ai_quality	Demographics: ai_quality	0.09781	0.0672	-0.03396	0.2296	1.103	0.9666	1.258	1.4548	0.146
Demographics: ai_lacks_creativity	Demographics: ai_lacks_creativity	0.04339	0.0644	-0.08290	0.1697	1.044	0.9204	1.185	0.6734	0.501
Demographics: ai_lacks_understanding	Demographics: ai_lacks_understanding	0.06126	0.0569	-0.05023	0.1728	1.063	0.9510	1.189	1.0769	0.282
Demographics: ai_grammar	Demographics: ai_grammar	-0.01654	0.0630	-0.14001	0.1069	0.984	0.8693	1.113	-0.2625	0.793
Demographics: ai_avoidance	Demographics: ai_avoidance	0.12247	0.0533	0.01793	0.2270	1.130	1.0181	1.255	2.2961	0.022
Demographics: ai_dumbing	Demographics: ai_dumbing	0.01103	0.0540	-0.09478	0.1168	1.011	0.9096	1.124	0.2042	0.838
Demographics: ai_fascination	Demographics: ai_fascination	0.01445	0.0739	-0.13032	0.1592	1.015	0.8778	1.173	0.1956	0.845

Random Components

Groups	Name	SD	Variance	ICC
Participant ID	More readable is human0	0.8707	0.75812	
	More readable is human1	1.6450	2.70616	
	Confidence	0.2783	0.07746	
	Reaction time log	0.2246	0.05044	
	GLS Euclidean distance	0.2031	0.04125	
	GLS1 difference	0.6851	0.46940	
	GLS2 difference	0.6633	0.43995	
	GLS3 difference	0.1631	0.02660	
	GLS4 difference	0.0705	0.00497	
	id	0.0590	0.00348	
	GLS8 difference	0.1607	0.02584	
	GLS7 difference	0.1277	0.01631	
	GLS6 difference	0.1719	0.02954	
	GLS5 difference	0.2500	0.06249	
Residuals		1.0000	1.00000	.

Note. Number of Obs: 2074 , groups: Participant ID 122

Random Parameters correlations

Groups	Param.1	Param.2	Corr.
Participant ID	More readable is human0	More readable is human1	-0.74893
	More readable is human0	Confidence	0.69299
	More readable is human0	Reaction time log	0.14039
	More readable is human0	GLS Euclidean distance	-0.11741
	More readable is human0	GLS1 difference	0.00524
	More readable is human0	GLS2 difference	-0.05658
	More readable is human0	GLS3 difference	0.16093
	More readable is human0	GLS4 difference	0.14308
	More readable is human0	id	0.39597
	More readable is human0	GLS8 difference	-0.05064
	More readable is human0	GLS7 difference	0.10114
	More readable is human0	GLS6 difference	0.32191
	More readable is human0	GLS5 difference	0.30439
	More readable is human1	Confidence	-0.36201
	More readable is human1	Reaction time log	-0.10605
	More readable is human1	GLS Euclidean distance	0.18802
	More readable is human1	GLS1 difference	0.07698
	More readable is human1	GLS2 difference	-0.43645
	More readable is human1	GLS3 difference	-0.04425
	More readable is human1	GLS4 difference	0.28439
	More readable is human1	id	-0.20016
	More readable is human1	GLS8 difference	0.09590
	More readable is human1	GLS7 difference	-0.08440
	More readable is human1	GLS6 difference	-0.14409
	More readable is human1	GLS5 difference	0.22300
	Confidence	Reaction time log	0.62084
	Confidence	GLS Euclidean distance	5.82e-4
	Confidence	GLS1 difference	0.13458
	Confidence	GLS2 difference	-0.32724
	Confidence	GLS3 difference	-0.16975
	Confidence	GLS4 difference	-0.02755
	Confidence	id	0.70806
	Confidence	GLS8 difference	-0.56421
	Confidence	GLS7 difference	0.27740
	Confidence	GLS6 difference	0.70559
	Confidence	GLS5 difference	0.43945
	Reaction time log	GLS Euclidean distance	-0.47991
	Reaction time log	GLS1 difference	0.33138
	Reaction time log	GLS2 difference	-0.31651
	Reaction time log	GLS3 difference	-0.23088
	Reaction time log	GLS4 difference	-0.15295
	Reaction time log	id	0.90143
	Reaction time log	GLS8 difference	-0.85447
	Reaction time log	GLS7 difference	0.29589
	Reaction time log	GLS6 difference	0.86667
	Reaction time log	GLS5 difference	0.18886
	GLS Euclidean distance	GLS1 difference	-0.42465
	GLS Euclidean distance	GLS2 difference	0.31367
	GLS Euclidean distance	GLS3 difference	-0.41959
	GLS Euclidean distance	GLS4 difference	-0.36307
	GLS Euclidean distance	id	-0.63713
	GLS Euclidean distance	GLS8 difference	0.30621
	GLS Euclidean distance	GLS7 difference	0.09862
	GLS Euclidean distance	GLS6 difference	-0.48188
	GLS Euclidean distance	GLS5 difference	0.02764
	GLS1 difference	GLS2 difference	-0.57781
	GLS1 difference	GLS3 difference	0.57626

Random Parameters correlations

Groups	Param.1	Param.2	Corr.
	GLS1 difference	GLS4 difference	0.46697
	GLS1 difference	id	0.29781
	GLS1 difference	GLS8 difference	-0.64415
	GLS1 difference	GLS7 difference	0.52507
	GLS1 difference	GLS6 difference	0.66018
	GLS1 difference	GLS5 difference	0.49995
	GLS2 difference	GLS3 difference	-0.57099
	GLS2 difference	GLS4 difference	-0.81916
	GLS2 difference	id	-0.47639
	GLS2 difference	GLS8 difference	0.35691
	GLS2 difference	GLS7 difference	0.09307
	GLS2 difference	GLS6 difference	-0.49190
	GLS2 difference	GLS5 difference	-0.48264
	GLS3 difference	GLS4 difference	0.85285
	GLS3 difference	id	-0.00918
	GLS3 difference	GLS8 difference	0.03398
	GLS3 difference	GLS7 difference	-0.26463
	GLS3 difference	GLS6 difference	0.03420
	GLS3 difference	GLS5 difference	0.04830
	GLS4 difference	id	0.13398
	GLS4 difference	GLS8 difference	0.10999
	GLS4 difference	GLS7 difference	-0.30214
	GLS4 difference	GLS6 difference	0.09777
	GLS4 difference	GLS5 difference	0.33781
	id	GLS8 difference	-0.67682
	id	GLS7 difference	0.09342
	id	GLS6 difference	0.84847
	id	GLS5 difference	0.25491
	GLS8 difference	GLS7 difference	-0.51185
	GLS8 difference	GLS6 difference	-0.88612
	GLS8 difference	GLS5 difference	-0.25024
	GLS7 difference	GLS6 difference	0.54724
	GLS7 difference	GLS5 difference	0.66339
	GLS6 difference	GLS5 difference	0.53784

E Generalized Mixed Model Report (Correctness, participants that did not get feedback)

Generalized Mixed Model

Model Info

Info	Value	Comment
Model Type	Logistic	Model for binary y
Call	glm	Is correct ~ 1 + More readable is human + Confidence + Demographics: age + Demographics: education_ordinal + Demographics: ai_usage_ordinal + Demographics: gender merged + Demographics: field_merged + Reaction time log + GLS Euclidean distance + GLS1 difference + GLS2 difference + GLS3 difference + GLS4 difference + id + Demographics: reading_disorder_merged + GLS8 difference + GLS7 difference + GLS6 difference + GLS5 difference + Demographics: ai_improve + Demographics: ai_risk + Demographics: ai_quality + Demographics: ai_lacks_creativity + Demographics: ai_lacks_understanding + Demographics: ai_grammar + Demographics: ai_avoidance + Demographics: ai_dumbing + Demographics: ai_fascination + (0 + More readable is human + Confidence + Reaction time log + GLS Euclidean distance + GLS1 difference + GLS2 difference + GLS3 difference + GLS4 difference + id + GLS8 difference + GLS7 difference + GLS6 difference + GLS5 difference | Participant ID)
Link function	Logit	Log of the odd of y=1 over y=0
Direction	P(y=1)/P(y=0)	P(Is correct = 1) / P(Is correct = 0)
Distribution	Binomial	Dichotomous event distribution of y
LogLikel.	-1287.871	Unconditional Log-Likelihood
-2*LogLikel.	2575.743	Unconditional absolute deviance
Deviance	1994.258	Conditional relative deviance
R-squared	NaN	Marginal
R-squared	NaN	Conditional
AIC	2851.740	Less is better
BIC	3640.553	Less is better
Residual DF	2106.000	
Chi-squared/DF	0.738	Overdispersion indicator
Converged	yes	
Optimizer	bobyqa	

Note. R-squared cannot be computed.

Note. boundary (singular) fit: see ?isSingular

Model Results

Fixed Effect Omnibus tests

	X^2	df	p
More readable is human	89.7178	1.00	< .001
Confidence	8.0146	1.00	0.005
Demographics: age	1.8030	1.00	0.179
Demographics: education_ordinal	0.4735	1.00	0.491
Demographics: ai_usage_ordinal	0.0771	1.00	0.781
Demographics: gender merged	0.7445	2.00	0.689
Demographics: field_merged	1.7027	4.00	0.790
Reaction time log	1.1086	1.00	0.292
GLS Euclidean distance	3.7821	1.00	0.052
GLS1 difference	12.4916	1.00	< .001
GLS2 difference	6.2612	1.00	0.012
GLS3 difference	5.0625	1.00	0.024
GLS4 difference	5.7322	1.00	0.017
id	1.1919	1.00	0.275
Demographics: reading_disorder_merged	1.0745	1.00	0.300
GLS8 difference	1.4838	1.00	0.223
GLS7 difference	0.6111	1.00	0.434
GLS6 difference	0.0648	1.00	0.799
GLS5 difference	2.5921	1.00	0.107
Demographics: ai_improve	1.2544	1.00	0.263
Demographics: ai_risk	0.1927	1.00	0.661
Demographics: ai_quality	0.0296	1.00	0.863
Demographics: ai_lacks_creativity	0.0253	1.00	0.874
Demographics: ai_lacks_understanding	1.2504	1.00	0.263
Demographics: ai_grammar	12.8382	1.00	< .001
Demographics: ai_avoidance	1.7876	1.00	0.181
Demographics: ai_dumbing	1.0169	1.00	0.313
Demographics: ai_fascination	0.0266	1.00	0.871

Fixed Effects Parameter Estimates

				95% Confidence Interval			95% Exp(B) Confidence Interval			
Names	Effect	Estimate	SE	Lower	Upper	exp(B)	Lower	Upper	z	p
(Intercept)	(Intercept)	1.07430	0.3503	0.38767	1.76092	2.928	1.474	5.818	3.0666	0.002
More readable is human1	1 - 0	2.06307	0.2178	1.63617	2.48996	7.870	5.135	12.061	9.4719	< .001
Confidence	Confidence	-0.13657	0.0482	-0.23113	-0.04202	0.872	0.794	0.959	-2.8310	0.005
Demographics: age	Demographics: age	0.01868	0.0139	-0.00859	0.04594	1.019	0.991	1.047	1.3428	0.179
Demographics: education_ordinal	Demographics: education_ordinal	0.08890	0.1292	-0.16431	0.34210	1.093	0.848	1.408	0.6881	0.491
Demographics: ai_usage_ordinal	Demographics: ai_usage_ordinal	0.01728	0.0623	-0.10473	0.13929	1.017	0.901	1.149	0.2777	0.781
Demographics: gender merged1	male - female	-0.01249	0.1846	-0.37436	0.34938	0.988	0.688	1.418	-0.0676	0.946
Demographics: gender merged2	other - female	-0.44541	0.5175	-1.45971	0.56890	0.641	0.232	1.766	-0.8607	0.389
Demographics: field_merged1	human - cs	-0.38585	0.3722	-1.11542	0.34372	0.680	0.328	1.410	-1.0366	0.300
Demographics: field_merged2	nature - cs	-0.29247	0.3946	-1.06583	0.48089	0.746	0.344	1.618	-0.7412	0.459
Demographics: field_merged3	other - cs	-0.36696	0.4746	-1.29717	0.56326	0.693	0.273	1.756	-0.7732	0.439
Demographics: field_merged4	philology - cs	-0.51098	0.4184	-1.33099	0.30904	0.600	0.264	1.362	-1.2213	0.222
Reaction time log	Reaction time log	-0.08757	0.0832	-0.25057	0.07544	0.916	0.778	1.078	-1.0529	0.292
GLS Euclidean distance	GLS Euclidean distance	0.09811	0.0504	-7.66e−4	0.19699	1.103	0.999	1.218	1.9448	0.052
GLS1 difference	GLS1 difference	-0.30027	0.0850	-0.46679	-0.13376	0.741	0.627	0.875	-3.5344	< .001
GLS2 difference	GLS2 difference	-0.33332	0.1332	-0.59441	-0.07224	0.717	0.552	0.930	-2.5022	0.012
GLS3 difference	GLS3 difference	0.09330	0.0415	0.01203	0.17458	1.098	1.012	1.191	2.2500	0.024
GLS4 difference	GLS4 difference	0.11974	0.0500	0.02172	0.21776	1.127	1.022	1.243	2.3942	0.017
id	id	-0.01233	0.0113	-0.03447	0.00981	0.988	0.966	1.010	-1.0917	0.275
Demographics: reading_disorder_merged1	true - false	0.71990	0.6945	-0.64126	2.08105	2.054	0.527	8.013	1.0366	0.300
GLS8 difference	GLS8 difference	-0.04195	0.0344	-0.10945	0.02555	0.959	0.896	1.026	-1.2181	0.223
GLS7 difference	GLS7 difference	0.03550	0.0454	-0.05350	0.12450	1.036	0.948	1.133	0.7817	0.434
GLS6 difference	GLS6 difference	0.00881	0.0346	-0.05902	0.07664	1.009	0.943	1.080	0.2545	0.799
GLS5 difference	GLS5 difference	-0.08746	0.0543	-0.19392	0.01901	0.916	0.824	1.019	-1.6100	0.107
Demographics: ai_improve	Demographics: ai_improve	0.08242	0.0736	-0.06181	0.22666	1.086	0.940	1.254	1.1200	0.263
Demographics: ai_risk	Demographics: ai_risk	-0.02545	0.0580	-0.13910	0.08820	0.975	0.870	1.092	-0.4389	0.661
Demographics: ai_quality	Demographics: ai_quality	-0.01152	0.0670	-0.14286	0.11981	0.989	0.867	1.127	-0.1720	0.863
Demographics: ai_lacks_creativity	Demographics: ai_lacks_creativity	-0.01039	0.0654	-0.13853	0.11774	0.990	0.871	1.125	-0.1590	0.874
Demographics: ai_lacks_understanding	Demographics: ai_lacks_understanding	0.07057	0.0631	-0.05313	0.19427	1.073	0.948	1.214	1.1182	0.263
Demographics: ai_grammar	Demographics: ai_grammar	0.23112	0.0645	0.10469	0.35754	1.260	1.110	1.430	3.5830	< .001
Demographics: ai_avoidance	Demographics: ai_avoidance	-0.07768	0.0581	-0.19155	0.03619	0.925	0.826	1.037	-1.3370	0.181
Demographics: ai_dumbing	Demographics: ai_dumbing	0.05286	0.0524	-0.04988	0.15559	1.054	0.951	1.168	1.0084	0.313
Demographics: ai_fascination	Demographics: ai_fascination	-0.01330	0.0816	-0.17320	0.14661	0.987	0.841	1.158	-0.1630	0.871

Random Components

Groups	Name	SD	Variance	ICC
Participant ID	More readable is human0	0.9265	0.85845	
	More readable is human1	1.0687	1.14205	
	Confidence	0.3027	0.09162	
	Reaction time log	0.1454	0.02113	
	GLS Euclidean distance	0.1909	0.03645	
	GLS1 difference	0.4111	0.16902	
	GLS2 difference	0.6810	0.46382	
	GLS3 difference	0.1320	0.01743	
	GLS4 difference	0.1857	0.03448	
	id	0.0494	0.00244	
	GLS8 difference	0.0338	0.00114	
	GLS7 difference	0.1193	0.01423	
	GLS6 difference	0.1179	0.01389	
	GLS5 difference	0.1486	0.02208	
Residuals		1.0000	1.00000	.

Note. Number of Obs: 2244 , groups: Participant ID 132

Random Parameters correlations

Groups	Param.1	Param.2	Corr.
Participant ID	More readable is human0	More readable is human1	-0.1357
	More readable is human0	Confidence	0.7366
	More readable is human0	Reaction time log	-0.5014
	More readable is human0	GLS Euclidean distance	-0.3042
	More readable is human0	GLS1 difference	0.4502
	More readable is human0	GLS2 difference	-0.7730
	More readable is human0	GLS3 difference	-0.1628
	More readable is human0	GLS4 difference	0.1273
	More readable is human0	id	0.8072
	More readable is human0	GLS8 difference	0.6546
	More readable is human0	GLS7 difference	0.5294
	More readable is human0	GLS6 difference	0.0181
	More readable is human0	GLS5 difference	0.1757
	More readable is human1	Confidence	-0.6185
	More readable is human1	Reaction time log	-0.1658
	More readable is human1	GLS Euclidean distance	-0.2715
	More readable is human1	GLS1 difference	-0.3666
	More readable is human1	GLS2 difference	0.1657
	More readable is human1	GLS3 difference	0.1708
	More readable is human1	GLS4 difference	-0.2312
	More readable is human1	id	-0.0489
	More readable is human1	GLS8 difference	0.0591
	More readable is human1	GLS7 difference	-0.2807
	More readable is human1	GLS6 difference	-0.0932
	More readable is human1	GLS5 difference	-0.8651
	Confidence	Reaction time log	-0.1095
	Confidence	GLS Euclidean distance	-0.0626
	Confidence	GLS1 difference	0.6796
	Confidence	GLS2 difference	-0.8108
	Confidence	GLS3 difference	-0.2743
	Confidence	GLS4 difference	0.1229
	Confidence	id	0.5153
	Confidence	GLS8 difference	0.4863
	Confidence	GLS7 difference	0.2745
	Confidence	GLS6 difference	-0.1273
	Confidence	GLS5 difference	0.6892
	Reaction time log	GLS Euclidean distance	0.7467
	Reaction time log	GLS1 difference	0.0868
	Reaction time log	GLS2 difference	-0.0835
	Reaction time log	GLS3 difference	0.4857
	Reaction time log	GLS4 difference	0.5059
	Reaction time log	id	-0.0521
	Reaction time log	GLS8 difference	-0.4404
	Reaction time log	GLS7 difference	-0.6837
	Reaction time log	GLS6 difference	-0.7138
	Reaction time log	GLS5 difference	0.1175
	GLS Euclidean distance	GLS1 difference	-0.0488
	GLS Euclidean distance	GLS2 difference	-0.0866
	GLS Euclidean distance	GLS3 difference	0.6903
	GLS Euclidean distance	GLS4 difference	0.7521
	GLS Euclidean distance	id	0.1383
	GLS Euclidean distance	GLS8 difference	-0.1221
	GLS Euclidean distance	GLS7 difference	-0.4406
	GLS Euclidean distance	GLS6 difference	-0.6644
	GLS Euclidean distance	GLS5 difference	-0.0733
	GLS1 difference	GLS2 difference	-0.6408
	GLS1 difference	GLS3 difference	-0.5700
	GLS1 difference	GLS4 difference	0.3898

Random Parameters correlations

Groups	Param.1	Param.2	Corr.
	GLS1 difference	id	0.1957
	GLS1 difference	GLS8 difference	0.6171
	GLS1 difference	GLS7 difference	-0.2782
	GLS1 difference	GLS6 difference	0.0360
	GLS1 difference	GLS5 difference	0.3820
	GLS2 difference	GLS3 difference	-0.0440
	GLS2 difference	GLS4 difference	-0.3499
	GLS2 difference	id	-0.7701
	GLS2 difference	GLS8 difference	-0.5650
	GLS2 difference	GLS7 difference	0.0128
	GLS2 difference	GLS6 difference	0.5049
	GLS2 difference	GLS5 difference	-0.2486
	GLS3 difference	GLS4 difference	0.3614
	GLS3 difference	id	0.3990
	GLS3 difference	GLS8 difference	-0.3698
	GLS3 difference	GLS7 difference	-0.0685
	GLS3 difference	GLS6 difference	-0.7594
	GLS3 difference	GLS5 difference	-0.3239
	GLS4 difference	id	0.4316
	GLS4 difference	GLS8 difference	0.2738
	GLS4 difference	GLS7 difference	-0.3431
	GLS4 difference	GLS6 difference	-0.4114
	GLS4 difference	GLS5 difference	-0.1346
	id	GLS8 difference	0.3300
	id	GLS7 difference	0.3650
	id	GLS6 difference	-0.4468
	id	GLS5 difference	0.0170
	GLS8 difference	GLS7 difference	-0.0369
	GLS8 difference	GLS6 difference	0.1233
	GLS8 difference	GLS5 difference	-0.1925
	GLS7 difference	GLS6 difference	0.4333
	GLS7 difference	GLS5 difference	0.3075
	GLS6 difference	GLS5 difference	0.0860

F Generalized Linear Model Report (Questionaire data)

Generalized Linear Model

Model Info

Info	Value	Comment
Model Type	Linear	Classical Regression/ANOVA
Call	glm	Correctness ~ 1 + `Demographics: ai_improve` + `Demographics: ai_risk` + `Demographics: ai_quality` + `Demographics: ai_lacks_creativity` + `Demographics: ai_lacks_understanding` + `Demographics: ai_grammar` + `Demographics: ai_avoidance` + `Demographics: ai_dumbing` + `Demographics: ai_fascination`
Link function	Identity	Coefficients in the same scale of y
Distribution	Gaussian	Normal distribution of residual
R-squared	0.0634	Proportion of reduction of error
AIC	1308.2390	Less is better
BIC	1347.1930	Less is better
Deviance	2315.6847	Less is better
Residual DF	245	
Chi-squared/DF	9.4518	Overdispersion indicator
Converged	yes	Whether the estimation found a solution

Model Results

Loglikelihood ratio tests

	X^2	df	p
Demographics: ai_improve	3.72321	1	0.054
Demographics: ai_risk	0.00116	1	0.973
Demographics: ai_quality	0.57658	1	0.448
Demographics: ai_lacks_creativity	0.01647	1	0.898
Demographics: ai_lacks_understanding	0.01334	1	0.908
Demographics: ai_grammar	6.92022	1	0.009
Demographics: ai_avoidance	0.18220	1	0.669
Demographics: ai_dumbing	0.01316	1	0.909
Demographics: ai_fascination	0.00153	1	0.969

Parameter Estimates

Names	Estimate	SE	95% Confidence Interval Lower	95% Confidence Interval Upper	exp(B)	95% Exp(B) Confidence Interval Lower	95% Exp(B) Confidence Interval Upper	z	p
(Intercept)	10.21176	0.193	9.83442	10.589	27221.563	18665.331	39699.99	53.0413	< .001
Demographics: ai_improve	0.30956	0.160	-0.00488	0.624	1.363	0.995	1.87	1.9296	0.055
Demographics: ai_risk	-0.00452	0.133	-0.26511	0.256	0.995	0.767	1.29	-0.0340	0.973
Demographics: ai_quality	0.11452	0.151	-0.18107	0.410	1.121	0.834	1.51	0.7593	0.448
Demographics: ai_lacks_creativity	0.01817	0.142	-0.25926	0.296	1.018	0.772	1.34	0.1283	0.898
Demographics: ai_lacks_understanding	0.01553	0.134	-0.24795	0.279	1.016	0.780	1.32	0.1155	0.908
Demographics: ai_grammar	0.37848	0.144	0.09649	0.660	1.460	1.101	1.94	2.6306	0.009
Demographics: ai_avoidance	0.05114	0.120	-0.18368	0.286	1.052	0.832	1.33	0.4268	0.670
Demographics: ai_dumbing	-0.01414	0.123	-0.25573	0.227	0.986	0.774	1.26	-0.1147	0.909
Demographics: ai_fascination	-0.00679	0.173	-0.34668	0.333	0.993	0.707	1.40	-0.0392	0.969
